# Access to Nitrogen–nitrogen Bond-Containing Heterocycles Through Substrate Promiscuity of Piperazate Synthases

**DOI:** 10.1021/acscatal.5c01237

**Published:** 2025-05-12

**Authors:** Yongxin Li, Angelina Osipyan, Niels A.W. de Kok, Simon Schröder, Maria Founti, Peter Fodran, Ronald van Merkerk, Artur Maier, Dirk Tischler, Sandy Schmidt

**Affiliations:** 1 Department of Chemical and Pharmaceutical Biology, Groningen Research Institute of Pharmacy, 3647 University of Groningen, Antonius Deusinglaan 1, Groningen 9713 AV, The Netherlands; 2 Faculty of Biology and Biotechnology, Microbial Biotechnology, 9142 Ruhr University Bochum, Universitätsstraße 150, Bochum 44780, Germany

**Keywords:** biocatalysis, *N*-heterocycles, diamines, *N*-hydroxylating monooxygenases, piperazate synthases, nitrogen−nitrogen bond-forming enzymes

## Abstract

The nitrogen–nitrogen (N–N) bond motif comprises an important class of compounds for drug discovery. Synthetic methods are primarily based on the modification of N–N or N=N precursors, whereas selective methods for direct N–N coupling offer advantages in terms of atom economy and yield. In this context, enzymes such as piperazate synthases (PZSs), which naturally catalyze the N–N cyclization of l-*N* ^5^-hydroxyornithine to the cyclic hydrazine l-piperazate, may allow an expansion of the current narrow range of chemical approaches for N–N coupling. In this study, we demonstrate that PZSs are able to catalyze the conversion of various *N*-hydroxylated diamines, which are different from the natural substrate. The *N*-hydroxylated diamines were obtained *in situ* using *N*-hydroxylating monooxygenases (NMOs), allowing subsequent cyclization by PZS, ultimately forming the N–N bond to yield various N–N bond-containing heterocycles. Using bioinformatic tools, we identified NMO and PZS homologues that exhibit distinct activity and stereoselectivity profiles. The screened panel yielded 17 hydroxylated diamines and more promiscuous NMOs, thereby expanding the substrate range of NMOs, resulting in the formation of previously poorly accessible *N*-hydroxylated products as substrates for PZS. The investigated PZSs led to a series of 5- and 6-membered cyclic hydrazines, and the most promiscuous catalysts were used to scale up and optimize the synthesis, yielding the desired N–N bond-containing heterocycles with up to 45% isolated yield. Overall, our data provides essential insights into the substrate promiscuity and activity of NMOs and PZSs, further enhancing the potential of these biocatalysts for an expanded range of N–N coupling reactions.

## Graphical abstract

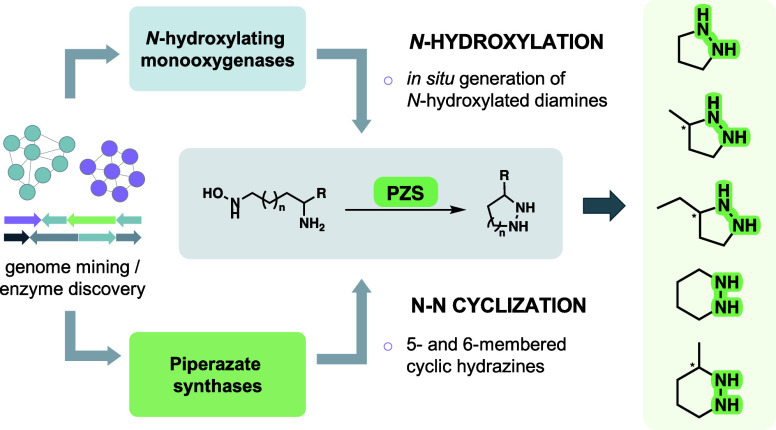

## Introduction

The nitrogen–nitrogen (N–N) bond is present in a wide variety of building blocks and is a highly valuable motif in the pharmaceutical and fine chemical industries. In particular, N–N bond-containing heterocycles have shown remarkable biological activity and are used as anti-inflammatory drugs (e.g., phenylbutazone **1**),^1^ protease and kinase inhibitors for the treatment of a variety of diseases associated with the targeted proteases and kinases (e.g., DB7461 **2**),^2^ clinical reagents (e.g., Levosimendan **3**),^3^ and as angiotensin-converting enzyme inhibitors (e.g., Cilazapril **4**)^4^ (Figure 1A). More than 300 natural metabolites containing N–N bonds have been isolated from a variety of organisms and have potential as therapeutic agents and precursors for the synthesis of biologically active molecules.^5–8^

**Figure 1:**
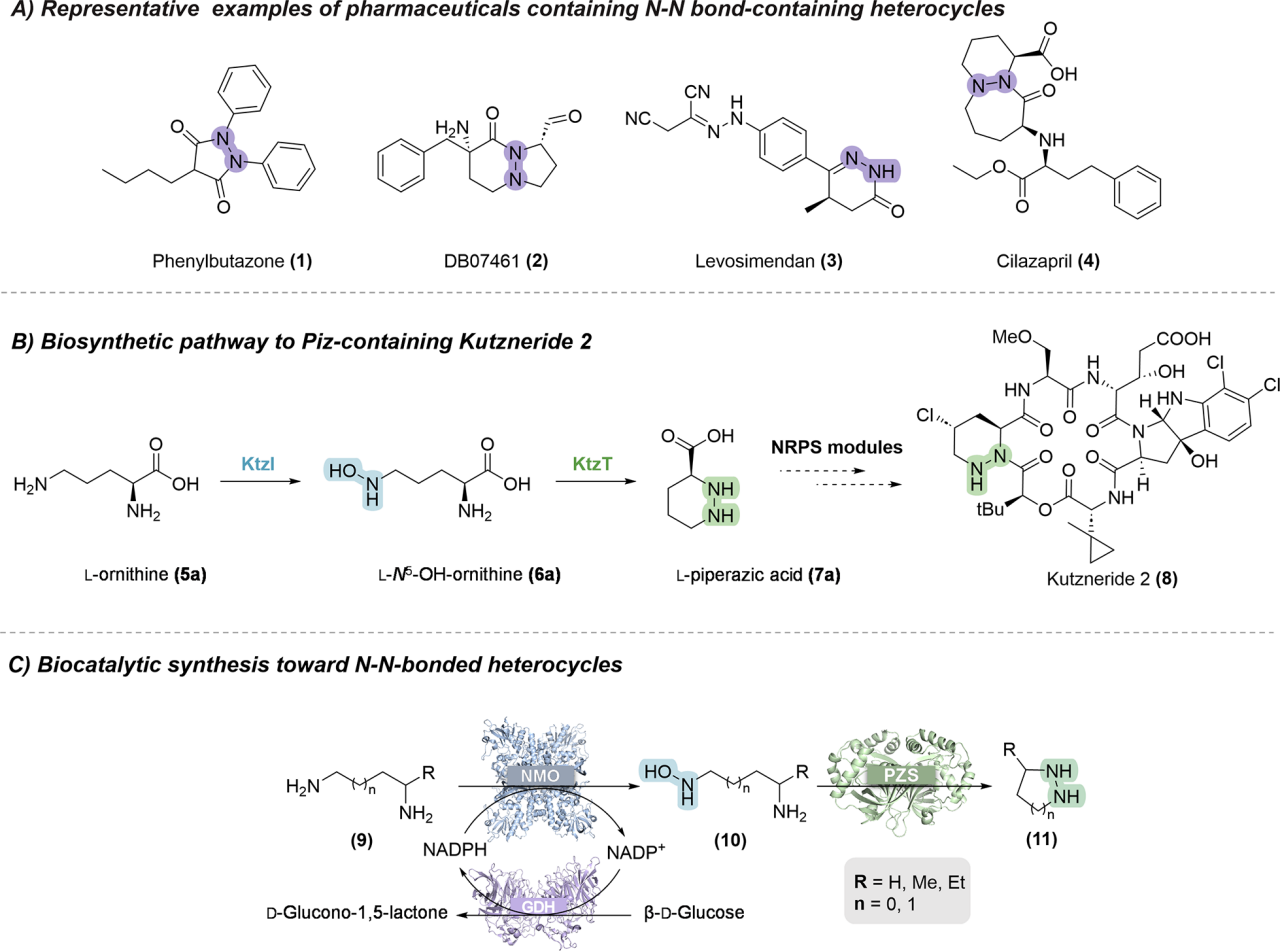
(A) Representative examples of N–N bond-containing pharmaceuticals. (B) Biosynthesis of l-piperazic acid in *Kutzneria* sp. 744 catalyzed by the *N*-hydroxylating monooxygenase (NMO) KtzI and the piperazate synthase (PZS) KtzT. (C) Overview of the biocatalytic synthesis toward N–N-bonded heterocycles, comprising *N*-hydroxylation catalyzed by an *N*-hydroxylating monooxygenase (NMO) and N–N bond formation catalyzed by a piperazate synthase (PZS). Glucose dehydrogenase (GDH) is used to regenerate the cosubstrate NADPH.

Despite advances in organic chemistry, the accessibility of N–N bond-containing compounds remains a significant challenge, and synthetic methods are mainly based on the modification of N–N or N = N precursors such as hydrazine and diazo compounds.^9–12^ Direct N–N coupling may provide a more convergent synthesis strategy, allowing greater retrosynthetic flexibility. However, direct N–N coupling remains difficult. For example, the conventional synthesis of cyclic hydrazines, such as piperazic acid (systematic name: (*S*)-hexahydropyridazine-3-carboxylic acid; abbreviated as Piz) requires at least nine steps,^13,14^ group protection and deprotection, ultimately resulting in low overall yields (around 20%).^15–17^ Moreover, N–N coupling often requires activation of nitrogen-containing molecules. One of the methods used by nature is the *N*-hydroxylation of amine groups. *N*-Hydroxylation of amino acids and diamines are crucial for the synthesis of value-added metabolites and nitrogen-containing compounds but remains challenging due to low yields and instability.^18–20^ Enzymes as environmentally friendly catalysts could expand the current narrow range of chemical approaches to N–N coupling without the need for metal catalysts or harsh reaction conditions. However, the biocatalysts capable of forming N–N bond-containing molecules, such as hydrazines, *N–*nitroso- and diazo-compounds, have only recently been elucidated.^21–31^ Among them are the enzymes involved in the biosynthesis of Piz **7a** in *Kutzneria* sp. 744, which allow the hydroxylation of the *N* ^5^ nitrogen in ornithine **5a**, catalyzed by a flavin-dependent *N*-hydroxylating monooxygenase (NMO), namely KtzI, to l-*N* ^5^-OH-ornithine (OH-Orn) **6a**. Subsequently, a heme-dependent enzyme, the piperazate synthase (PZS) KtzT, catalyzes the N–N cyclization of l-*N* ^5^-OH-Orn to the cyclic hydrazine Piz **7a** (Figure 1B and Figure 2).^32,33^ This compound is then incorporated into nonribosomal peptide synthetase (NRPS) or NRPS-polyketide synthase (PKS) hybrid pathways.^21^ Piz or its congeners such as 5-hydroxy-, 5-chloro-, and dehydro-piperazic acid can be integrated into more complex structures, e.g., kutznerides (e.g., **8,** Figure 1B), piperazimycins, padanamides, himastatins, or monamycines.^34,35^

**Figure 2:**
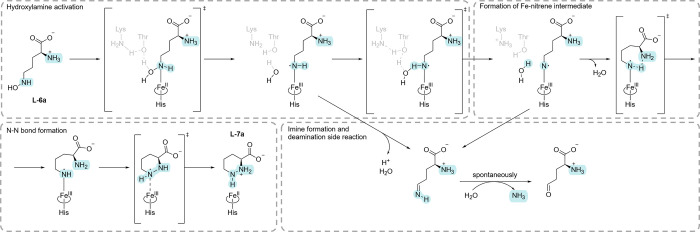
Mechanism of PZS-catalyzed conversion of l-*N* ^5^-hydroxy-l-ornithine (l **-6a**) to l-piperazic acid (l **-7a**) and potential deamination side reaction.^33^ Figure adapted from Ref.^31^.

NMOs catalyze the *N-*hydroxylation of diamines or diamino acids such as **5a**, generating the reactive intermediates for the formation of diverse N–O or N–N linkages. In this context, KtzT^21^ and its identified PZS homologues^36^ are promising candidates for the coupled synthesis of various N–N bond-containing compounds together with NMOs. Recent studies have shown that PZS-catalyzed N–N coupling is more likely to proceed via divergent pathways that may originate from a common nitrenoid intermediate that reverses the nucleophilicity of the hydroxylamine nitrogen in **6a** (Figure 2).^33,37^ Interestingly, a structurally different hydroxylamine (*N*-benzylhydroxylamine) was shown to undergo a deamination reaction instead of N–N bond formation compared to the natural substrate **6a**. Initial attempts were made to understand the substrate scope of PZS by studying derivatives of the natural substrate **6a** with shorter or longer chain lengths (l-*N* ^4^-OH-diaminobutyric acid (DABA) and l-*N* ^6^-hydroxylysine)^21,33^ or substrates with a terminal hydroxy group instead of an amino group (e.g., 2-amino-5-hydroxyvaleric acid).^38^ Notably, the substrate range appears to be limited to the formation of 5- and 6-membered α-hydrazino acids. In addition, mechanistic studies have revealed a side deamination reaction and spontaneous C–N bond formation.^33^ Recent studies also provide evidence for the feasibility of Piz production through the use of a chimeric NMO-PZS enzyme in engineered actinobacteria, supporting the potential utility of NMO-coupled reactions in the synthesis of valuable N–N bond-containing heterocycles.^39^

In this study, we demonstrate that PZSs can catalyze the conversion of various *N*-hydroxylated diamines that are different from the natural KtzT substrate **6a**. The *N*-hydroxylated diamines were obtained *in situ* using NMOs, allowing subsequent cyclization by PZSs, ultimately forming the N–N bond to yield various N–N bond-containing heterocycles (Figure 1C). Using bioinformatic tools, we identified novel NMO and PZS homologues that exhibit different activity and stereoselectivity profiles. The screened panel yielded 17 hydroxylated diamines and a new promiscuous NMO (*Sg*NMO), thereby expanding the substrate scope of poorly accessible hydroxylated products. We tested them against the panel of PZSs and identified 5 commonly accepted substrates. The most promiscuous catalysts, KtzT, *S*spMPZS and *A*spPZS, were used to scale up and optimize the synthesis, yielding the desired N–N bond-containing heterocycles with up to 45% isolated yield. The studied enzymes also exhibited an inverse enantiomeric preference, making them promising candidates for future enzyme engineering efforts. Overall, our data provides essential insights into the substrate promiscuity and activity of NMOs and PZSs, further enhancing the potential of these biocatalysts for an expanded range of N–N coupling reactions.

## Results and Discussion

### Enzyme Selection

All known piperazic acid-containing natural products identified to date, such as padanamides, matlystatins, and himastatin, predominantly originate from actinomycete bacteria.^40^ To investigate the promiscuity of PZSs, we initiated a sequence similarity network (SSN) analysis for this protein family using the characterized KtzT as a starting point (Uniprot ID: A8CF72, protein family number PF04299, Figure 3), which was constructed with 12,801 sequences. To reduce the number of entries, the threshold for protein length was set at 200 to 300 amino acids. In most cases, NMOs and PZSs are observed to colocalize in gene clusters containing NRPS assembly genes. In the past, putative PZS genes have often been overlooked in databases due to their annotation as transcriptional regulators and flavin-binding proteins.^21^ Hence, we identified three SSN clusters representing PZSs along with their putative NMOs based on genome neighborhood analysis (EFI-GNT, Figure S3). From the identified clusters, 18 genes encoding 8 putative NMOs and 10 putative PZSs from organisms known to synthesize piperazate-containing metabolites were selected to investigate their N–N bond-forming capabilities (Table S1). Consequently, the selected genes of NMOs and PZSs were cloned into an expression vector and heterologously expressed in *Escherichia coli* strain BL21 (DE3) with an N-terminal or C-terminal histidine tag, respectively, and purified with the exception of two putative NMOs (Uniprot ID: A0A1U9K2D1 and A0A5S4H6S6) and two putative PZSs (Uniprot IDs: A0A557ZX56, A0A2S8QH93), which could not be produced in soluble form in *E. coli* (Figure S2). In addition, one putative NMO was found to be unstable under the experimental conditions (Uniprot ID: A0A2T5KZR9).

**Figure 3:**
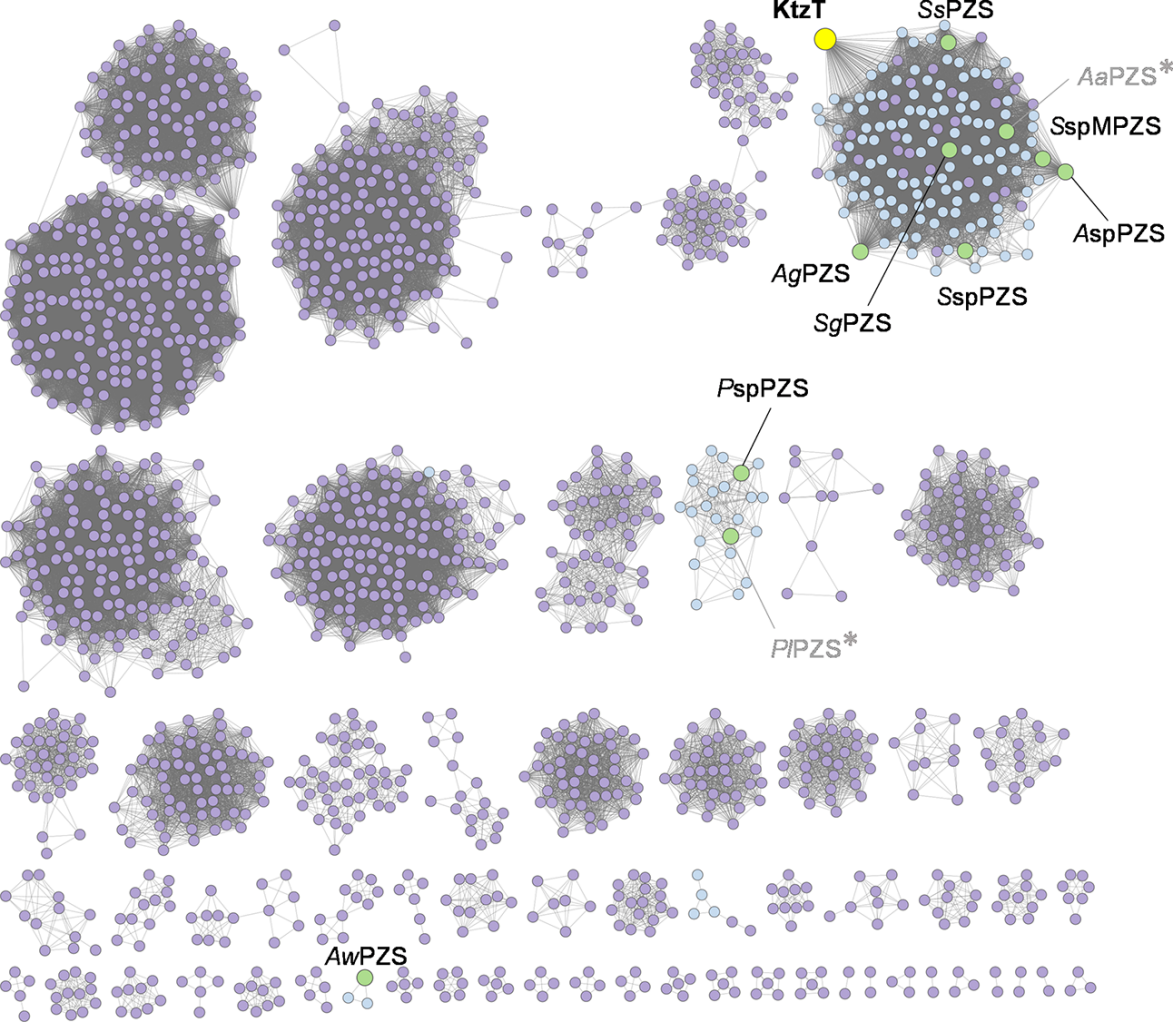
Sequence similarity network (SSN) of proteins assigned to the piperazate synthase (PZS) family. Nodes colored in green represent the selected putative PZSs. Blue nodes represent the enzymes that contain a putative monooxygenase (PF13434) in the gene neighborhood. The SSN was constructed using the EFI-EST Web server, employing an *E* value of 5 and alignment score of 52. The SSN was visualized using Cytoscape (Version 3.10.3). *Proteins did not express in the soluble fraction.

### Substrate Scope of the Hydroxylation Reaction

We then started exploring the promiscuity of NMOs by investigating a panel of substrates that included diamines, diamino acids and their derivatives (Figure 4A). Initially, the selected purified NMOs were screened for cosubstrate depletion in 96-well microplates in the presence of NADH and NADPH, respectively. Notably, NAD­(P)H depletion was also observed in the absence of a substrate or when the substrate was not converted (Figure S4), indicating the presence of nonproductive reactions, also referred to as oxygen uncoupling.^41^ Therefore, catalase was added to the reactions to prevent the accumulation of H_2_O_2_. Reactions containing enzymes and substrates that showed increased NAD­(P)H depletion compared to controls in the absence of substrates were then subjected to liquid chromatography–mass spectrometry (LC-MS) analysis to identify the corresponding product formed. To determine the amount of *N-*hydroxylated product by the selected NMOs, the Csáky assay^42^ was used to assess the degree of *N-*hydroxylation against a standard (hydroxylamine). NMOs are generally known to be highly substrate-specific, either for hydroxylating the amino group of diamines or amino acids. Furthermore, reported NMOs are mostly NADPH-specific, and we thus only used NADPH as a cosubstrate in further experiments.^43–45^ We found that two NMO homologues from *Streptomyces griseochromogenes* (*Sg*NMO) and *Streptomyces spongiae* (*Ss*NMO) accept the native substrate of KtzI **5a**, and *Sg*NMO is also capable of catalyzing the *N-*hydroxylation of several diamines (Figure 4B). In particular, *Sg*NMO is active on a variety of diamines with different carbon chain lengths, such as **9b** (3%), **9e** (15%) and **9h** (23%), compared to its natural substrate **5a** (82%). To our surprise, *Ss*NMO was not active against l-lysine (**5b**), but showed activity on lysine derivatives (**5d**, 42%) and ornithine methyl ester (**5c**, 5%). We thus decided to also include a known NMO, namely GorA, which was previously characterized by Esuola et al.,^44^ and the variant L237R in our substrate scope screening. In addition to the known substrates (**9a** and **9d**) that were previously reported to be converted by GorA, we identified several additional substrates to be accepted by GorA or its variant (**9b, 9c, 9g, 9j – 9n**) (Figure 4B, Table S2). Also in line with our expectations, no double hydroxylated products were detected by LC-MS for any of the NMOs tested. Overall, most of the diamines and diamino acids investigated, except for l-lysine (**5b**), were accepted by the selected NMO panel.

**Figure 4:**
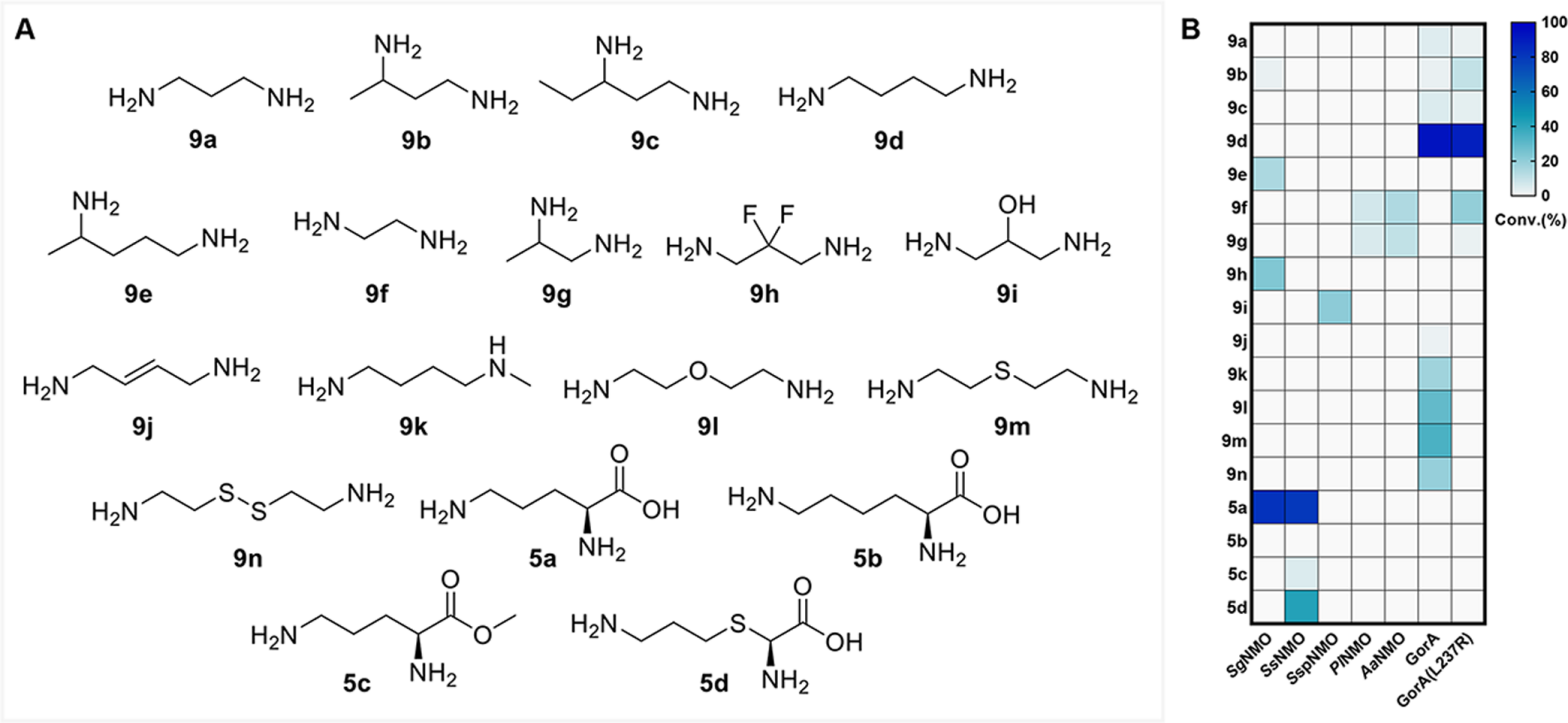
(A) Panel of substrates for promiscuity screening. Diamines **9a–9n** and diamino acids **5a–5d** vary in the carbon chain length; diamine analogs (**9b, 9c, 9e, 9g–9n**) contain different substitutions; natural substrates **9a**, **9d**, and **9f** of GorA; derivatives of diamino acids (**5c** and **5d**). (B) Csáky assay heatmap to determine *N*-hydroxylation of various NMOs toward all substrates. Reaction conditions: 1 mM substrate, 50 mM NaPi buffer pH 8.0, 10 U/mL glucose dehydrogenase (GDH), 10 mM glucose, 1 mM NADP^+^, 1 mg/mL catalase, 30 μM NMO, 100 μL reaction volume, incubation at 25 °C, 1 h. Data were obtained from triplicate measurements by means of Csáky assay results using hydroxylamine as a standard for a calibration curve.

### Substrate Scope of the Cyclization Reaction

We then sought to investigate the intramolecular N–N bond formation by coupled enzymatic cyclization of the activated substrate. To study this, we individually coupled the NMO-substrate pairs with the highest observed conversion for each substrate with each PZS. The substrate scope of PZS comprises the acceptance of **6a** and unsubstituted diamine derivatives **10a – 10e** (Figure 5). However, no activities were found against substrates **10f – 10n** and hydroxylated **5b – 5d** for any of the investigated homologues. KtzT appears to be the most promiscuous catalyst, and the most optimal for the synthesis of **7a**, **11a**, **11c**. For the formation of **11b** and **11e**, *S*spMPZS showed the highest activity and was selected together with KtzT for the enantiomeric ratio (e.r.) analysis. The other PZS homologues have a narrower substrate range or lower activity compared to KtzT and *S*spMPZS. For example, *Aw*PZS and *P*spPZS are unable to accept **6a**, which may be due to lower sequence identity to KtzT (34% and 25%, respectively). *Ss*pPZS belongs to the same cluster but displayed a rather limited substrate scope compared to KtzT. *As*pPZS, however, showed the highest activity for the formation of **11d** (Table S3). It is noteworthy that PZSs have been described to catalyze a deamination reaction rather than N–N bond formation,^33^ which should lead to the production of an imine (with subsequent hydrolysis to aldehyde) as a byproduct in the context of non-natural substrates. The formation of the proposed five-membered byproducts was only observed with substrates **9d** and **9e**, consistent with previous results (Figure S6),^33^ but the expected masses corresponding to aldehydes and four-membered imines were not detected for substrates **9a – 9c**, likely due to their inherent instability or low ionization efficiency under the given analytical conditions.

**Figure 5:**
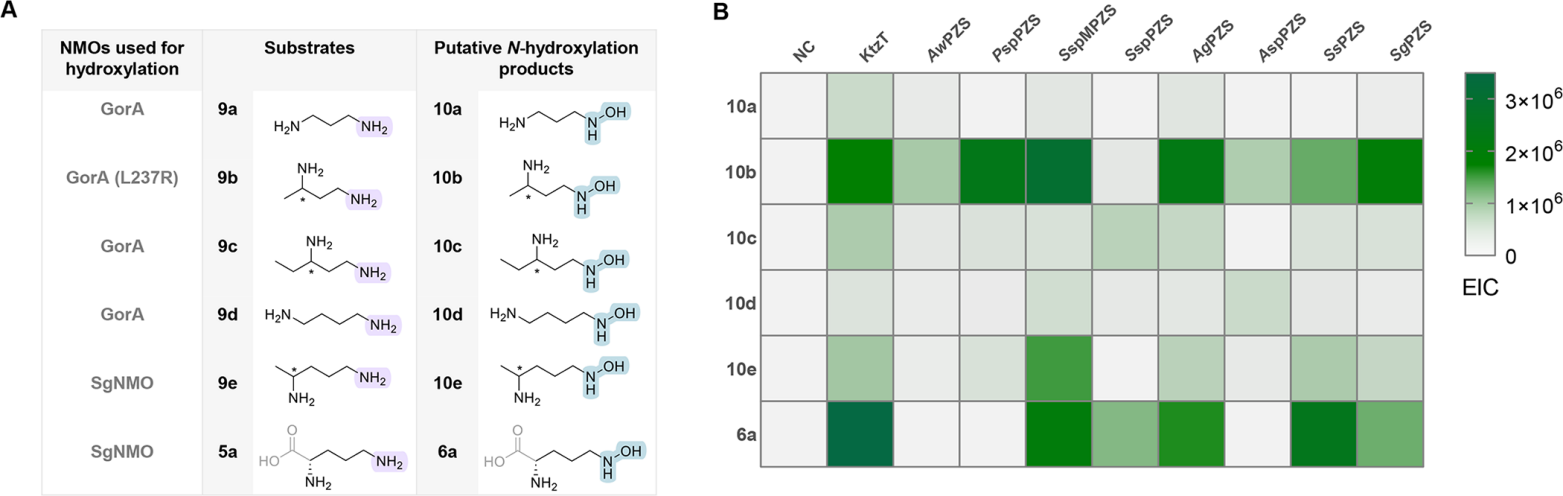
(A) Overview of selected NMOs for generating the *N*-hydroxylated substrates for PZSs. **The preparation of compound **7a** has only been carried out on an analytical scale (B) Heatmap indicating N–N bond-forming activity of tested PZSs. Reaction conditions: 20 mM NH_4_HCO_3_ pH 8.5, 1 mM substrate (**9a–9e**, **5a**), 1 mM NADP^+^, 0.05 mM FAD, 10 mM glucose, 10 U/mL GDH, 1 mg/mL catalase, 30 μM NMOs and 5 μM heme-containing PZS, 100 μL reaction volume, incubation at 25 °C, 1 h. Extracted ion chromatogram (EIC) of expected mono/bis-Fmoc were integrated (**11a**–**11e** and **7a**), assuming the ionization of all compounds are the same. NC is the control without the addition of PZS.

The evaluation of the coupled reaction mixture was performed through a comparison of the enzymatic cascade with the chemically synthesized references and the control reactions without PZSs. For the linear substrates **9a** and **9d**, the new products demonstrated the same retention time and identical mass (*m*/*z* 517.2122, *m*/*z* 531.2278) as the synthetic reference compounds **11a** and **11d** (Figure 6A, D). This confirms that the PZS homologues can form the N–N bond-containing products even in the absence of the carboxyl group present in their natural substrate **6a**. However, when substrate **9j** was used in the GorA-PZSs coupled cascade, no product with the expected mass was identified. Instead, the presence of the double bond in the carbon chain favors deamination of the substrate with subsequent formation of 1*H*-pyrrole (Figure S7). The use of branched propylenediamines (**9b**, **9c**, **9e**) in this cascade resulted in the formation of the corresponding products **11b** (*m*/*z* 531.2278, Figure 6B), **11c** (*m*/*z* 545.2435, Figure 6C) and **11e** (*m*/*z* 545.2435, Figure 6E), which possess a chiral center. To determine the stereoselectivity of PZSs, we performed the full cascade reaction with either the racemic substrate or an available pure enantiomer of **9b, 9c** and **9e** using KtzT and *S*spMPZS, respectively, together with GorA L237R, GorA and *Sg*NMO. As a result, the (*S*)-enantiomer of **11e** was obtained enzymatically using (*S*)-**9e** as a substrate, demonstrating a conservation of the stereocenter in the *Sg*NMO-KtzT-coupled cascade (Figure S17, entry 4). Moreover, the experiment performed with the substrate **9e** demonstrated a slight preference of KtzT toward the (*S*)*-*enantiomer, while *S*spMPZS yielded a racemic product (Figure S17, entries 2 and 3). Interestingly, the reaction with racemic **9b** using *Ss*pMPZS resulted predominantly in the formation of (*R*)-**11b**, whereas the reaction with KtzT yielded mainly the (*S*)-**11b** (Figure S15). Consequently, it is concluded that KtzT shows a preference for accepting the (*S*)-configured substrates, which is consistent with the results of the previous studies.^21^ In contrast, *S*spMPZS shows a substrate preference for the (*R*)*-*configuration in the conversion of **9b** and a lack of stereoselectivity in the conversion of **9c** and **9e** (Figure S15, entry 3; Figure S16, entry 3; Figure S17, entry 2).

**Figure 6:**
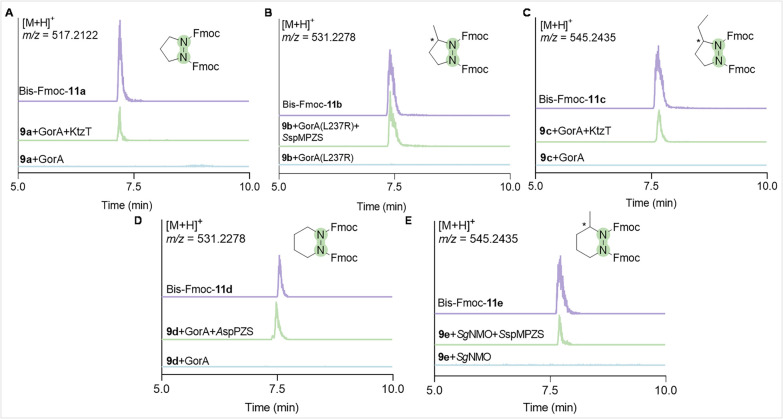
Extracted ion chromatogram (EIC) from LC-MS analysis of synthesized cyclic hydrazines in coupled NMO-PZS reactions, corresponding to Bis-Fmoc-**11a** (A), Bis-Fmoc-**11b** (B), Bis-Fmoc-**11c** (C), Bis-Fmoc-**11d** (D), and Bis-Fmoc-**11e** (E). Reaction conditions: 20 mM pH 8.5 NH_4_HCO_3_, 1 mM substrate (**9a**–**9e**), 1 mM NADP^+^, 0.05 mM FAD, 10 mM glucose, 10 U/mL GDH, 1 mg/mL catalase, 30 μM NMO (GorA for **9a**, **9c**, and **9d**, GorA L237R for **9b**, and *Sg*NMO for **9e**) and 5 μM heme-containing KtzT/*S*spMPZS/*A*spPZS, 100 μL reaction volume, incubation at 25 °C, 1 h. The reference compounds are indicated in purple, the controls in the absence of PZS are represented in blue. The *m*/*z* values are theoretical. The entire cascade is shown in green. All assays were conducted in duplicates.

We were intrigued by the apparent difference in enantiomeric preference between KtzT and *S*spMPZS in the conversion of chiral substrates **9b** and **9e**, and thus attempted to identify potential structural explanations by comparing AlphaFold models of both enzymes and performing substrate docking (Figure S18). Alignment of the structures revealed complete conservation among residues lining the active site, thus not explaining enantiomeric preference within the first shell. While the active site appears very large and exposed, the overall shape of the cavity is modeled to be slightly different for both enzymes, suggesting that remote variations could have an impact on molecular dynamics and substrate preference. Due to the large size and good accessibility of the active site, substrate docking experiments were also inconclusive. The diamine substrates appear to prearrange in a flatter conformation compared to the diamino acids (**6a**), possibly promoting the ring closure and compensating for the weaker coordination by the enzyme (Figure S19).

### Optimization of the Reaction Conditions and Upscale

To optimize the catalytic system for the synthesis of the cyclic hydrazines, we determined the optimal conditions for the formation of **11a** using the GorA-KtzT coupled reaction, taking into account the effects of temperature, buffer type, buffer concentration and pH. The reaction achieves highest substrate conversion at moderate temperature and buffer concentration, specifically around 25 °C and 20 mM (Figure 7A, B). The highest activity was observed at the pH of 8.5 (Figure 7C), suggesting that the deprotonation of the *N*-hydroxylated substrate facilitates nucleophilic attack. Furthermore, the highest amount of **11a** observed in the presence of NH_4_HCO_3_ buffer compared to the other buffers investigated (Figure 7C). Notably, the formation of **11a** was significantly reduced in the presence of additional NaCl (Figure 7D). This observation is consistent with the results of previous research indicating that KtzT has a preference for lower salt concentration.^32^ In the time-course experiment, the highest amount of **11a** was formed after incubating the reaction for 3 h, presumably due to the instability of the product in aqueous phase (Figure S8).

**Figure 7:**
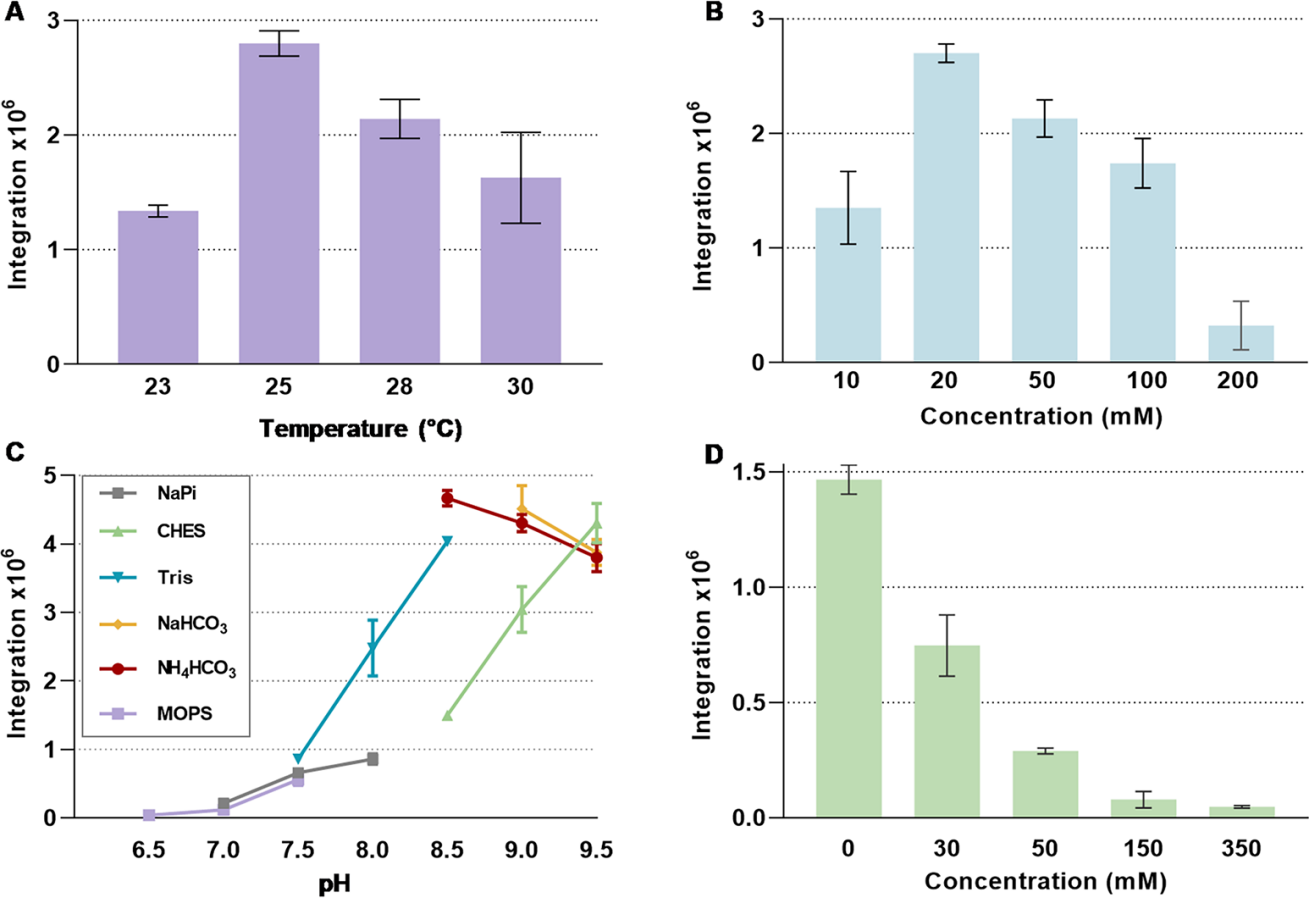
Temperature, buffer concentration, buffer and pH optimization, and salt tolerance (NaCl) for the biocatalytic synthesis of N–N bond-containing heterocycles using NMO and PZS. (A) Temperature optimization. (B). 10–200 mM NH_4_HCO_3_ buffer pH 8.5 was used for analysis of optimal buffer concentration. (C) The following buffers were investigated: 50 mM NaPi (pH 6.5–8.0), MOPS (pH 6.5–7.5), Tris (pH 7.5–8.5), NH_4_HCO_3_ (pH 8.5–9.5), NaHCO_3_ (pH 9.0–9.5), and CHES (pH 8.5–9.5). No NaCl was added. (D) Salt tolerance of the cascade comparing NaCl concentrations (from 20 to 350 mM). The assay mixtures for above experiments contained 20 mM NH_4_HCO_3_ (except for panel B and C), no NaCl (except for panel D), 1 mM NADP^+^, 0.05 mM FAD, 1 mg/mL catalase, 10 mM glucose, 10 U/mL GDH, and 1 mM **9a**; the reaction was initiated by adding 30 μM GorA and 2 μM KtzT, and was incubated for 3 h at 25 °C (except for panel A) in a total reaction volume of 100 μL. Samples were analyzed by HPLC by integrating the area for Bis-Fmoc-**11a**. Results are derived from triplicate measurements.

In order to verify the structure of the products formed, the reactions were carried out on a mg scale under the optimal conditions determined. The reaction was performed using 2 mM substrate **9a – 9e** in a total volume of 15 mL. To achieve full conversion, the NMO concentration was increased to 50 μM. Despite the significant formation of deamination products, *S*spMPZS and *A*spPZS exhibited the highest product yields for **11d** and **11e** and were therefore selected as catalysts for preparative scale synthesis (Figure S6). Extraction of the cyclic hydrazines proved to be a significant challenge, resulting in low isolated yields due to their high solubility in the aqueous phase. To overcome this problem, the reaction products were derivatized with Fmoc prior to extraction with ethyl acetate. Subsequently, the derivatized products were isolated and the structures were confirmed by NMR and HRMS with comparison to chemically synthesized standards. The isolated yields for five-membered products **11a – 11c** ranged from 33% to 45% (Figure 8). Notably, six-membered products **11d** and **11e** were obtained with significantly lower yields (9 **–** 13%), likely due to predominant side reactions of the corresponding hydroxylamine intermediates.

**Figure 8:**
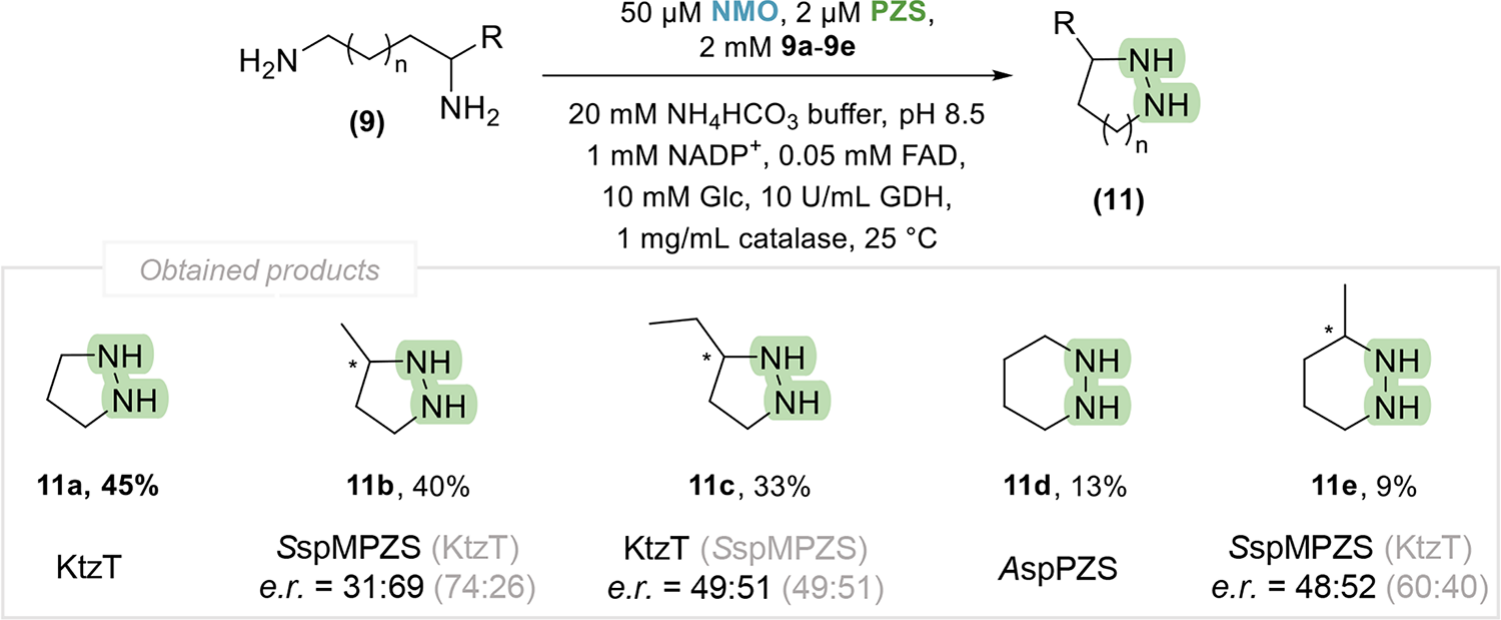
Product scope obtained with the NMO-PZS catalysis approach toward N–N bond-containing heterocycles. R = H, Me, Et; *n* = 0, 1. Reaction conditions: 20 mM NH_4_HCO_3_ buffer pH 8.5, 2 mM substrate (**9a**–**9e**), 1 mM NADP^+^ and 0.05 mM FAD, 10 mM glucose, 10 U/mL GDH, 1 mg/mL catalase, 50 μM NMO (GorA for **11a**, **11c**, and **11d**; GorA L237R for **11b**; and *Sg*NMO for **11e**), 2 μM PZS, 15 mL reaction volume, incubation at 25 °C, 5 h. The enantiomeric ratio (*e.r*.) was calculated by (*S*)- to (*R*)-enantiomer. The isolated yields are reported for the enzymes, showing the highest activity according to the heatmap.

## Conclusions

N–N bond-containing heterocyclic scaffolds have high synthetic value for the preparation of more complex functionalized molecules. In this study, we established the biocatalytic synthesis of cyclic hydrazines by exploiting the substrate promiscuity of both NMOs and PZSs. Our genome-mining strategy led to the discovery of a novel NMO (*SgNMO*) with the ability to accept both diamines and diamino acids, expanding the known NMO substrate scope. In addition, *S*spMPZS was shown to exhibit the opposite stereoselectivity to KtzT. However, the achievable product scope of wild-type PZSs appears to be limited to five- to six-membered heterocycles, which currently appears to be the main bottleneck in the diversity of cyclic hydrazines that can be formed using nonengineered PZSs. To identify the optimal reaction conditions, we investigated the influence of various reaction parameters, including buffer type, pH, salt concentration, and temperature. To further increase the yield of cyclic hydrazines, protein engineering to suppress the unwanted deamination reaction or *in situ* product removal strategies could be explored. In addition, the detection of N–N bond-containing heterocycles is currently dependent on LC-MS due to the low stability and high polarities of the resulting molecules. In light of these observations, the development of a suitable high-throughput spectroscopy-based detection method would facilitate further research into the formation of N–N bond-containing molecules using PZS. In summary, the range of compounds accessible by PZS is expanding, allowing access to more complex N–N bond-containing molecules. It is expected that this biocatalytic synthesis will be further extended in the future to enable the biocatalytic synthesis of a wider range of cyclic hydrazines with different structural diversity using engineered NMO/PZS couples.

## Supplementary Material



## Notes

Additional tables and figures demonstrating enzyme activity, NMR spectra, and HPLC data (PDF)
