# Supplementary material for: Access to Nitrogen–nitrogen Bond-Containing Heterocycles Through Substrate Promiscuity of Piperazate Synthases
Source: ACS Catal. 2025 May 12;15(11):8846–54. doi: 10.1021/acscatal.5c01237 (PMC12150314; doi:10.1021/acscatal.5c01237)
Supplement: Supplementary file 1 [file cs5c01237_si_001.pdf]

Supporting information for

Access to nitrogen-nitrogen bond-containing heterocycles through substrate promiscuity of piperazate synthases

Yongxin Li,[1] Angelina Osipyan,[1] Niels de Kok,[1] Simon Schröder,[1], Maria Founti,[1] Peter Fodran,[1] Ronald van Merkerk,[1] Artur Maier,[2] Dirk Tischler,[2] Sandy Schmidt[1,*]

[1] *Department of Chemical and Pharmaceutical Biology, Groningen Research Institute of Pharmacy, University of Groningen, Antonius Deusinglaan 1, 9713 AV Groningen, The Netherlands*

[2] *Ruhr University Bochum, Faculty of Biology and Biotechnology, Microbial Biotechnology, Universitätsstraße 150, 44780 Bochum, Germany*

*Corresponding author: s.schmidt@rug.nl

Contents

1. EXPERIMENTAL SECTION **3**

2. DNA SEQUENCES OF NMOS AND PZSS **6**

3. SUPPORTING FIGURES AND TABLES **10**

GENOMIC CONTEXT OF SELECTED GENES 12

4. EVALUATION OF SUBSTRATE PROMISCUITY OF NMOS **13**

6. HPLC TRACES OF ALL COMPOUNDS **21**

SEMI-PREPARATIVE HPLC DATA 21
CHIRAL HPLC DATA 24

7. SYNTHETIC PROCEDURES AND NMR SPECTRA OF COMPOUNDS **28**

8. HRMS DATA OF ALL COMPOUNDS **43**

9. REFERENCES **45**

1. Experimental Section

1.1. General Procedures

All reagents were purchased from commercial sources, such as Sigma-Aldrich, TCI, BLDpharm, Amdeed and Biosynth etc., and were used without further purification. The heme assay kit (ab272534) for heme determination was purchased from Abcam (Cambridge, UK). NMR spectra was recorded on a Bruker DRX 500 MHz or 600 MHz spectrometer, with *δ* in parts per million (ppm) and *J* in Hertz (Hz) and referenced to the residual solvent peak at 7.26 ppm (^{1}H) or 77.16 ppm (^{13}C) for deuterated chloroform ($CDCl_3$). The multiplicity of ^{1}H NMR signal was reported as singlet (s), doublet (d), triplet (t), quartet (q), and multiplet (m). High-resolution mass spectrometry (HRMS) was performed using a Shimadzu LC20 XR series with a Thermo Scientific Q exactive Plus mass detector. HPLC analyses were performed on a Shimadzu LC-10AT equipped with a photodiode array detector. HPLC coupled mass spectrometry (LC-MS) analysis was performed on a Waters Acquity Arc equipped with a 2998 PDA and an Acquity QDa quadrupole mass detector. Semi preparative HPLC was performed on a Shimadzu LC-8A equipped with a photodiode array detector to separate the reaction mixture. Substrate screening of NMOs was performed using a microplate spectrophotometer (SPECTROstar Microplate Reader).

1.2. Bioinformatic selection of NMO and PZS homologs for screening

To select candidates for functional screening, the Pfam identifier referring to the PF04299 protein family was used as input to create a sequence similarity network (SSN) via the EFI-EST web server (http://efi.igb.illinois.edu/efi-est/).[1, 2] The network was adjusted by deleting edges with low alignments scores of 45 until the PZS was separated into an isofunctional cluster, which was filtered at an alignment score value of 52 (corresponds to a pairwise sequence identity ~50%). The EFI-GNT tool (http://efi.igb.illinois.edu/efignt/) provided the genome neighborhood plots for each individual cluster in the input SSN. Visualization was performed using Cytoscape Version 3.10.3. NMOs and PZSs from selected clusters were cloned, expressed, purified, and screened for activity on a panel of selected substrates.

1.3. *Expression, purification and analysis of recombinant proteins*

Codon optimized homologs were cloned into pET28a from Twist Bioscience (USA). *E. coli* BL21 (DE3) (Invitrogen) was used for expression of all NMO and PZS homologs. Expression and purification of His-tagged NMOs and PZSs were performed by first preparing preculture in 50 mL LB liquid medium supplemented with 50 μg/mL kanamycin in 250 mL baffled Erlenmeyer flasks. From the overnight culture, 1 L LB medium was inoculated into a 3 L baffled flask to an initial cell density of OD_{600} 0.1 and incubated at 37°C, 120 rpm. Gene expression was induced at a cell density of ~0.8 by adding 100 μM isopropyl β-D-1-thiogalactopyranoside (IPTG), with additional supplementation of 1 mM 5-aminolevulinic acid (5-ALA), and 1 mM $(NH_4)_2Fe\ (SO_4)_2$, respectively. After the cells were grown at 20 °C for 20 hours, the cells were harvested and resuspended in lysis buffer (20 mM NH_4HCO_3, 10 mM imidazole, pH 8.0, 10% glycerol) and disrupted by sonication (70% duty cycle, power control of 7, 5 cycles of 30 s ON/30 s OFF). The soluble fraction of the cell lysate was recovered after centrifugation (13,000 × *g* for 40 min). Nickel-nitrilotriacetic acid (Ni-NTA) resin (GE Healthcare, USA) was used to isolate His-tagged proteins. Cell free extracts (CFEs) were incubated with 2 mL (1 CV) Ni-sepharose for 1 hour at 4 °C. They were then washed using 5 CVs washing buffer (20 mM NH_4HCO_3, 30 mM imidazole, pH 8.0, 10% glycerol). Elution was performed by adding three times 2 CVs elution buffer (20 mM NH_4HCO_3, 300 mM imidazole, pH 8.0, 10% glycerol). Purified protein-containing fractions were confirmed by SDS-PAGE and then desalted using a PD-10 column according the manufacturer's instruction (Cytiva, USA) with storage buffer (20 mM NH_4HCO_3, pH 8.0, 10% glycerol). The desalted protein was concentrated, snap frozen in liquid nitrogen and stored at −80 °C for further use. Protein concentration was determined by the Bradford protein assay (Bio-Rad) using bovine serum albumin (BSA) to generate a standard curve.

1.4. In vitro biochemical assays and Csáky assay to determine N-hydroxylation

A preliminary screening of NMOs was performed by monitoring NADH/NADPH depletion (ε340nm = 6.2 mM^{-1} cm^{-1}). The reaction mixture contained 1 mM substrate, 50 mM NaPi buffer pH 8.0, 10 U/mL glucose dehydrogenase (GDH), 10 mM glucose, 1 mM NAD(P)H, 1 mg/mL catalase, 30 µM NMO, 100 µL reaction volume, incubation at 25 °C. Hits were further confirmed by derivatization with Fmoc-Cl followed by LC-MS analysis. The amount of hydroxylated product formed by NMOs from different substrates (the same as those used in the NAD(P)H oxidation assay above except the addition of 1 mM $NADP^+$) was determined separately using the Csáky iodine oxidation reaction described previously.[3] Briefly, the assay mixture (100 µL per sample) was quenched with 40 µL of 1.5 M perchloric acid and centrifuged at 13,000 × *g* for 10 minutes. Subsequently, 40 µL of the supernatant was added to 96-well plates and neutralized with 50 µL of 10% (w/v) sodium acetate solution. To each well, 50 µL of 1% (w/v) sulfanilic acid in 25% (v/v) acetic acid, and 20 µL of 0.5% (w/v) iodine in glacial acetic acid were added and the reaction was allowed to incubate at room temperature for 15 minutes. Excess iodine was reduced with 20 µL of 0.1 M sodium thiosulfate and the color was developed by adding 20 µL of 0.6% (w/v) α-naphthylamine in 30% (w/v) acetic acid. The reaction was monitored at 562 nm. Hydroxylamine hydrochloride was used to generate a standard curve to extrapolate the amount of hydroxylated product formation.

1.5. In vitro biochemical assays and products analysis

The reaction mixture (100 µL) for the biochemical assay for the NMO-PZS-coupled reaction contained 30 µM NMOs, 5 µM heme-containing PZS, 1 mM substrate **9a – 9n** and **5a – 5d**, 1 mM $NADP^+$ and 0.05 mM FAD, 10 mM glucose, 10 U/mL GDH, 1 mg/mL catalase in 20 mM Tris–HCl buffer (pH 8.0) and was incubated at 25 °C for 1 hour. For LC–MS analysis, 100 µL of the reaction mixtures were quenched with 2 volumes acetonitrile and centrifuged at 13,000 x *g* for 5 minutes. For derivatization, 150 µL of the supernatant was taken and mixed with 25 µL borate buffer (0.2 M, pH 8.3) and 20 µL of 20 mM Fmoc-Cl and incubated for 10 minutes. After centrifugation at 13,000 x *g* for 5 minutes, 20 µL of the resulting supernatant was subjected to LC–MS analysis. Samples were separated on a XBridge BEH C18 column (2.1 × 50 mm, 3.5 µm) with a linear gradient of water and acetonitrile ((v/v) both containing 0.1% (v/v) formic acid: 80:20 to 10:90, 0 to 7min; 10:90, 7 to 9 min) at a flow rate of 0.5 mL/min.

1.6. One-pot cascade optimization

Starting from the screening of experimental conditions (100 µL containing 2 mM **9a**, 1 mM $NADP^+$ and 0.05 mM FAD, 10 mM glucose, 10 U/mL GDH, 1 mg/mL catalase), the enzymatic reaction conditions were optimized with GorA-KtzT towards **9a**. The reaction mixture was derivatized with Fmoc-Cl and subjected to reversed-phase HPLC using a Phenomenex Luna C18 column (4.6 × 250 mm, 5 µm). Elution was performed at 1 mL/min with a linear gradient of water and acetonitrile ((v/v): 30:70 to 10:90, 0 to 20 min; 10:90, 20 to 30 min), both containing 0.1% (v/v) trifluoroacetic acid (detection wavelength: 254 nm). The extracted chromatograms for the formation of **11a** were integrated and normalized by comparison with a reference compound.

1.7. Semi-preparative biocatalytic reaction

Enzymatic reactions were performed in 250 mL glass Erlenmeyer flasks with a total volume of 15 mL to facilitate aeration. The reaction mixtures consisted of 20 mM NH_4HCO_3 buffer pH 8.5, 2 mM substrate (**9a – 9e**), 1 mM $NADP^+$ and 0.05 mM FAD, 10 mM glucose, 10 U/mL GDH, 1 mg/mL catalase. The reaction was started by adding NMOs (50 µM) and 2 µM KtzT/SspMPZS/AspPZS. The reaction mixture was incubated at 100 rpm at 25°C for 5 h. To facilitate product isolation and subsequent analysis, 4 equivalents of Fmoc-Cl (31 mg) were added to the mixture and stirred at 300 rpm for 2 h at room temperature under a nitrogen atmosphere to stabilize the non-derivatized enzymatic products. The derivatized reaction components were then extracted with 2 × 15 mL EtOAc. After vacuum

concentration, the resulting crude extract was redissolved in acetonitrile and subjected to semi-preparative HPLC for product isolation. By comparing the retention time and absorbance spectra of the resulting peaks with reference compounds, the fractions containing Bis-Fmoc-N-N bond-containing products were collected. Preparative-HPLC was performed on a Nucleodur® C18 column (10 × 250 mm, 5 μm) and compounds were eluted with an isocratic mixture of 70% acetonitrile and 30% water (detection wavelength 210 nm). Fractions of interest were analyzed by LC-MS to assess purity and confirm the presence of the expected mass. The NMR spectra was recorded on a Bruker DRX spectrometer at 500 MHz or 600 MHz using dimethyl sulfoxide-d_6 (DMSO-d_6) as the solvent. The enantiomeric ratio of isolated products was determined by reverse phase HPLC using a CHIRALPAK® OD-RH column (4.6 × 150 mm, 5 μm, Daicel) with isocratic 90% acetonitrile and 10% water, 1 mL/min, 40 °C, detection wavelength at 220 nm.

2. DNA sequences of NMOs and PZSs

> A8CF85, KtzT, *Kutzneria* sp. (strain 744)

atgtttgttccaggtccatatcatgcaccagaagatcgttggcttgttgatcttgttcgtggtcatccacttgcacaacttgcatctaatggtgcaggtggtgcagcacc
acatattactcatgttccaattattgttgatccagaacttgatggtccagttgatcgtcttgttggtattactctttggggtcatatgaatcgtgcaaatccacattgggca
gcacttggtggtgcagcaaatgttgttgcaacttttgcaggtccaaatgcatatgtttctccagcagtttatcgtactgcaccagcagcaccaacttggaattttacttc
tgttcaagttcgtggtgaacttcgtaaagttgaatctgcagatgatactcttgcaactgttcgtgcaactgttgcagcacttgaatctcgttttggtgcaggttgggatat
gactggttctcttgattattttcgtcgtattcttccaggtgttggtgcatttcgtcttcgtgttgcagaagcagatggtatgtttaaactttctcaagaacaacaaccagca
attcgtcgtcgtgttcgtcattcttttggtggttgtgaagcaactcgtgcagttgcaggtcttatggatcgtcttccaactgaa

> A0A1B1AU19, *Sg*NMO, *Streptomyces griseochromogenes*

atgagtgatcgtgaacatgaaacctatgacgtggttggcattgggtttggtcctagcaacctgtcactcgccatagcactggaagagtatcgtgcgaatggtccg
gaaaatgagatcagcgccttatttctggagcgtcaatcagcgttcggctggcatcgcaacatgctgttgccgtcaacgaccatgcaaatttccttcctcaaagatct
ggttacttttcgcaatccgacgtcgagttttagctttatcgcttacttgcacgcgtcggggcgtctaccgcagttcgtcaacaaccaggattttttcccacgcgtcagg
agtttcaccagtacttggaatgggctcaggcacgtgtggccgatcgcgtcgcgtacggttctgaagtgacttctattcgtctgcctccgggtgccgatccggaacg
gtcggaccgtctgcgattagaagtggcggatgcaacagggcgcaatggccgcgtggtcgaagcacgtaacgtggtgatctcgaccggcctggtcccgagca
tgccagtgggaaccgaacgcgatgaacgtgtttggcacagttcagagtttctggaaaaatatcgccgtatgaatcctgcggaactgcgtcgggtggcggtggtt
ggggcgggacagagcgccgccgagattaccagatttctgtacgatgaacttccgcatgcagaggtttgcgctgttatcccgagctatggctattccgtagcggat
gacaccccattcgctaatcagattttcgaccccggggccgttgatgactattacttcggcacagagcaaacgcgggaagcgttctggcgttaccatcgcaatac
gaactactccgtagtagacgatgaggtgattcgggatctttatcgcaggtcttacgatgacgaagtacggggtgtgcgtcgccttcagtttttaaacctgacgaga
gtaaccagtgtgaagcgcgtcggcgccgaaacacgcgtctccctgcaggtgggcccggatgatgaagtgcgcgaacttgatttcgatgcattggtttgtgcgac
cggttatagcacgatggaaccaactgacctgttgggcgatctcgatcgtcattgtctgcgcgacgaggcgggccgatatagagttgaaagggactatcgcattg
ttacagctccagaaatgcgctgcggtatttatttacaaggtggtactgagcatacccacggactgacctcctctctgctgagtaatatcgctgtacgaagcggtga
gatagccgactcgatcgtcgcaggtcgtgcgggacgcaacgcagaacgtgcactcttagccgaagtcggcggcgatacccgctaa

> A0A1B1AU73, *Sg*PZS, *Streptomyces griseochromogenes*

atgtttgtaccaagccattaccgagaaccggacgtttcatggatggtcgatttaatgcgcggtaacccgctggcactgatggcctccaatggaactcccgctgac
ggccctttcgcgacgcatttgccggtcatcaccgatccccagtgggagggcagccctaccgcggacctggcgggcatgccgctgctgggtcacatgaaccgt
gccaatccgcactgggcggcgctggaaaccggttcggcgatcctgttgacgtttaccggtccgcacgcatacgtttccccaacagtctatgacgtcacgcctgc
cgccccgacatggaactttacttcggtgcatgtgcatggagttgtggaaaaaattgagtctacggaagagaccttagatgttgtacaggctacagtgcaagcatt
cgaaggggaatttggcgatagttgggatatgagcgaatctgttgattattttcgcaaaattgtgaccggcgtcggcgctttccgtgttcgggtgactaaggcagag
ggtatgttcaaactcagtcaagaacagcgtccagaaattcgcgagcgcgtggtgcagtcatttgccgggcgcgaatgcaccagacatgtacagacggcggat
cttatgaatcgtctaccg

> A0A5N8XG21, *Ss*NMO, *Streptomyces spongiae*

atggaaaaacatagcgatgtacatgaggtagttggcattggttttggcccttccaatctgtccctggccattgcactggaagagcacgaagccaagcgtgacgg
tcacccgatttcagctgcgtttttgaaaaacagccaacgttaggctggcatcgcaacatgctgctgccgagcgcgaccatgcaaatttctttttaaaagacctgg
ctacctttcgtaatccggcttctcaattcagttttgttaattatctgcacgcggcaggtcgcttggcccagtttgttaacaatcagagtttcttcccgacacgtgaggaatt
tcacgattatcttgagtgggccgagtccagcttctcggatcgagtcagctacagcagtgaagtgacttcagtgcggttaccccaggggggcgggccagtggac
cacgtcgaagttgcggtccgcgggacgggtccgcgcggtgccacacgcgtggttaaagcccataacattgtggtgtcgactggtttggtgccgcgcatgcctgc
aggtatagatcgcgaccagcgcatctggcacagctcggaattcctcgaaaaatttcgtaaattgacacaggattctgtgaagagggtggcagttatcggcgctg
gacagagcgctgcagaaatcgttcgctttgtctacgattctattcccggcgccactgtctccgcgattatgccatcttatgggtattcaattgcggatgataccccttttc
gccaacaaaatcttcgatgcatcagcggtggacgactactacaattgtggtgagcgttcgaaagacgccttctggcgttatcacaaaaacaccaactatggcgt
cgtagacgatgaagtaatacgtgacctacatcaacgtgcgtatgatgatgaagttcgcggcaccccaagactggattttgtgaatctgtcgaaggtcgatagcg
cgaaacagacaggcgacaaagtgcgtctgaccctgcatagtctgcgcagcgatgagacgagtgatttggatgtcgatgtgcttatctgcgcgacgggctatga
ttcaatggagccgagcgaacttcttggcgaactggaaccgcattgcctgcgcgacgaacaaggccgttttcgggtggaacgggactatcgtctcgcaaccgg
accggaagtaacttgtggtgtttacttacaaggaggtaccgagcatacccatggactggcgtcctcactgctctccaacattgccgtacgtagtggggaaatcgc
agattcgatcgctgaaagacgaacgaatcggcagtacagcacggcgtaa

> A0A5N8XFV2, *Ss*PZS, *Streptomyces spongiae*

Atgttcgtaccggcaccataccgaacaccagccacttcatggatggtggatattctgcgcggcaatccgctggcgttaatgacatccaacggcccagcgaccg
cgggtccctgggcaactcatctgccggtaatccaggaccctacgatgacggaagattggtcagaggacctcagcggtgcgaagttactaggccacatgaatc
gtgcaaacccgcattggaaagatttggggatggggccgtggttctgctgaccttcaccggacctcacggttatgttagtccgacggtatatgaaaaaacacca
gctgcaccgacctgggacttcacctcggtgcatgtccatggcacgttgactaaaatcgaaccggaagaagcaggtgaagaatcgctggaactggtgtgcagc
acggtccgtgcctttgaaggagatttcggcgctggctgggatatgcgggagtctatagactactttcgtaaaattcttcctgccgtgggtgcctttcgcgttgatgtga
ccggtgcggaggggatgtttaaactcagccaagaacaggatccggacgtccgcgagcgtgttcgtgagtcttttgccggcagagactccacccgtcaccgcg
aagcgagtcgccttatggatcggctgccccgcccggatattggctgtccgcatgcggct

> U5YL02, *S*spNMO, *Streptomyces* sp. RJA2928

atgacagatagcgcaccagaggatcgcaccgttgacgtcacgggaatcggtttcggtccgtcgaatctggcgcttgcgaccgcgctcgccgagccctctgcaa
ccggtcctggcaggccgttggaagcggtctactttgaacgcaaaaaccggttttcctggcacggcggaatgctgttggatggcgctaccatgcagatttccttttg
aaggatctggtgacgctgcgcgatccgcgaagtccgtacagctttctgagctatcttcatcatgccggccgcttatctgattttattaatcacaaactgctgttcccgt
cgcgtatagagtttcatgattatctggagtgggttgccggctttttcgaagagcaggtcgtatatggttccgaggttgtggatgtgcgtccggtagctcgcgaagacg
cggttgaacacatggacgtcgtggtcaggcaacgcactgcagcaggggaacgtaccgtggtgcaacgcacccgcgacttagtagtggccaccggcttaga
accgtctctgcctccggggacggtttgttctgaccgcgtatggcacagctccgaactgttgtatcgtgtggagcgtcttcctcctacgccgcgccgtatcgtcgtggtt
ggtgccggacaaagcgcggctgaagcggcagagttcctgcacagccggttcccatcgacggacatttgcgcggtgttttcccgctacggctacagtccctcag
atgatagcccgttcgccaatcgtatttttgatccggcggctgttgatgactattgcgcggctgccccagaaaccagacgcatgctcctcgattatcatcgtaatacta
actattcggtggtcgatccggaactgattgacgaactgtatcgccgtgtttaccaggaaaaagttcgtggtcgtcccagactcaacatcttaggcgcatcgcgatt
gatggcggcggaaccggctggagacggtgtagatgtggtggtggaatcactggtcaccggtgaacgcactcctatgcgtgccgactgtgtggtctacgcgacc
ggttaccggccgacggatgcgcgcggcctgctgggcagcatggcgggtctgtgcaaagccgatgaacttggccgcctagaagccgatcgtcgttatcgcgtta
ttacagaaggggatgttcgttgtgcgatttatttacagggcgcaaccgaacatagtcatggtatcagctcaagtctgctgagtaacacagcagtacgtgctgggg
agatcgcggatgcaatccgggctgacgcagtacgtgccggggcccgggccactacgcgatcacagccacagccacagacataa

> U5YN79, *S*spPZS, *Streptomyces* sp. RJA2928

atgtttgtacctcagcactatcgtacggatgatcggcggtggccggtgcgtattgttcaagataatccgcttgcactgctgatgtcaacccgcgacggtcgtgcccc
gtttgccagccatgttccggtgattgtcctgccgcgtcagcgtgaagaattggaacgcactggccgctggcagggtgcggtgctgcacggccacatgaatcgcg
caaatccgcattggaagtccctggccgatgggcagccggcaggtttagttttccagggtcctgcgggctatgtctcccccgctgtctataacacttcgcctgcagtg
ccgacctggaactttaccgcggttcatgtgcagggaagactgaaattagtagcggatgaagaagctacgctgggcgttgtgagtgccaccgcccgccaattgg
aggagcgctttggggcgcgttggacagttgagccatcggtggaccattttcgtcagatcctcccaggcgtaggtgcattcgaactgcgtgtcgaagaatgcgact
caatgttcaaactatctcaagaaaaagagcacgaagtacgacatgctgtcatggattggtgtgcgcgcagcccacgaggaaggagcaacgatcttgccgcg
gtgatgcgcgactactacccgcccacgaccacatggccaagt

> A0A1U9K2D1, *S*spMNMO, Streptomyces sp. M41

atgggcataactggtagacgcagccaggagatttatgatgtggtcggtatcgggttcggcccgtcaaacctgagtttagcgattgcattggaggaacatggtgct
agcgctccggaacatccggtcacgtcccatttttcgagcgtcagccttcgtttggttggcaccgtaatatgctgctgccgtcgacgaccatgcaaatttcctttctca
aagacttggcgacctttaggaatcctatgagtcggtttagttttatctcatatttacatgccgctaatcgtctggtgcaatttgtgaacaaccaagatttttcccgaccc
gccaggagtttcaccagtacctggagtgggcggccgcgagcttaggcgaccgtgtaacatacggcgcggaagttaccgcgattcgtccaggaacggaaggt
ggggtggcccgtccagatctcgtcgaagtagaagtacgcgcgggtgatggcactcttagccgtgtgactgcccgcaatgtggccatttccacggcctggttcc
gcgtctgcctgaaggggtgagcgcggacgaacgtgtttggcatagctctcaattcctgggtcgcttcaatgaacaggatccggcaggcctaaaaagcgttgctg
ttgtgggcgccgggcagagcgctgctgaaatcacacgcttcctccacgacgcgctgccggacgcacaggtcagcgcggtcatcccatcatacggctactccg
tggccgatgataccccccttcgcaaaccaggtctttgaccccggagcggtagatgaatattattttggcaacgagcgtgcacaagatgcgttctggcgctatcacc
gcaataccaactactctgtggttgacgccgatgttatccagtcactctatcagcgactgtacgatgaacaagtgcgcggcagtcgtcgtctgcacttccgcaactt
aaccagagtcgcggaggtgaagcgagcagggaatgaaacgcgagttctgcttcggtctcttctggacgattcgacggaagaactggcagtagacgcgttggt
gttcgccactggatatgatgggctggacccggcccggctgctaggagattttgaccggcattttcagcgtgatgcggccggccgccaccgggtggaacgcgatt
atcgccttgttcccgcttctgatctgacctgcggtatctatctgcaaggcggcaccgaacatagtcatggtctgtcttcagcgctgttgtcgaacattgcagtgcgca
gtggtgaaattgccgattcgattgtattaggtcgcacagagcgtgaattggagcagggttgtccggttcaggcagaaccatccgcagtctaa

> A0A1V9K2B3, *S*spMPZS, *Streptomyces* sp. M41

atgtttgtgccaagcagttaccgtgaaccggatagttcttggatggtggatttaatccgcgccaatcctctggcactagcggcggccaacggtcgtccagaagac
ggcccgtttgccacccatctgccggtaatttttgatccagacacatctggggaatggacgggcgaactgcccggtgccactctcttgggacacatgaatcgggc
gaaccccccattgggcggcattggaatcgggtagcgtactgctgctgacgttcaccggtccgcactcctatgtctcaccgaccgtttatgaagttaccccggcagc
gccgacgtggaacttcacggctgtccatgtgcgtggtgtggtggaaaaaatcgattcggtgcaggaaaccctgggcgtcgttgaatccactgtacgcgccttcg
agggtcgttttggcgacggctgggatatgactgcatcacttggctattttcgcaaaattgcgcctgcagtgggagcgttccgctttacagttaccggggccgaaggt
atgttcaaactgagtcaggagcagcctggcgaagttcgtgagcgagtcagagagtcgtttgggcaaagcgcttgcacctacaagcgcgagacggctggctta
atgagccggcttccg

> A0A2S8QH87, *Pl*NMO, *Photorhabdus luminescens*

atgaaaaactcttcatcaatttatgatatcctcggtattggttttgggccggccaacatcgccctgtctattgcgctggaggaactggccccgcacttttcagtgcggtt
tattgagcgtcagcaaagcgcatgttggcagccaggaatgatgttgccgggctctgatattcaaaatcatccgctgcgtgatttggttacgccacgcaaccccaa
atcccactactcgttcaccaacttcctgtatgaaactgaccgattatatcagcaccttaatctgccactccattaccctctgcgtctcgaatacgcgcaatatatcag
ctgggcggcgaatttttcaacgaccaggtggattacgattgtgaggcgacagagatctttccggttctggacggccaaaacgaatacatcgaccattattgtgtg
aataccgtaaaaggagatatctatcgcgcgcgcagtattgtccttgctcccggccgtacgcctttcataccggaaccgtttaaccaactcaaagatgcgcgtgtg
gtgcatcttaaccattatttgccgtcactggaccaggccaggaaatacagtaactctctgagcgtggcggttattggcggcagtcagagcgcagtagaaattcta
ctacacgccagtacccaaaacgatatcaaaagactcgttggcgtcacccgcaattttgggttccgccagaaagatacgagccctttcagcgacgaggtgtattt
cccggaatttgtcgatacattctatgaagctacgccggaaaataaggcacgcctgcgtcgcgaactggtacacacgaactattcgtcggcagatattgacgttct
gaaccagctgtatattaagcagtatgaaaataaactgagccatcaaaattccatgcagatcttaacttgcaatgaaatcgttgattgccggagtatttcaaacggt
atatttctgaaaagtcgaaactttattaatagcaatgaatttgaagagtgctttgatttagtcgtactggccactggattccttgatttaggcaccggcgaacgccagg
agttttgcccaaaattattactgcccattaagaaactgctgaccaatcaggggggatatcctgtccataggccgggactaccgtgtgcagtacctgacgaagggg

gactttaccccgatttatctgaatggtttgtgtgaatcgacccatggtatgggtgatgctggttccttcagcttgcttgcattgcgttctaaaacaatcgtcgactcgctta
gccagtacctgtcctaa

> A0A2S8QH93, *Pl*PZS, *Photorhabdus luminescens*

atgattgaactgtctattgagtccaaaatatatagcgtgaagagtgaaaacatcattcaaatcgactttgaagttgataaaataattcagcgcatcatcaacattat
gaaaactttcccgtttcactatcatgaattcatttgcaaaaacgtagagcttttagaacgttttattgacgcgtacccgctggcactgatcacccgttacatgagcaat
ggtttcatcaccagccacattcctctgtttcaaggtgtggataaaatgctttttgggcatacagatcgtcgtaacccgatggtgaatgacctcgtgtcgtttgaagcgc
atatcgtcttcatggaccctccagctatatcccgccggaagcatataagaatcgccagctgcccacgtggaactatacggccgtgcacatggatgccagcatt
gacatcatcgtcgaatcttcaatgaatctaaacattttgctcaaaaccagtgagcggttggcgactggcaaaggctcgttccgaccaaataaggaagatcctcg
cgtcgttaagaatttaccggggattctgggcctgaaagttgtaccgaaatacattgaaggtcgctttaaattgtctcaggataaaccattcgttgaccatgagtcag
ctatgcagtggctgttagataacaatccatcggagtttgattccctgattaaaagtctgatcaacggc

> A0A557ZX73, *Aa*NMO, *Amycolatopsis acidiphila*

atgaatgaataccgcgatatcgtcggtgttggttttggacctgccaacctggcactgacggtggcacttgacgaagtgccgggcaacggcaccgataggccga
gtgcgctttttttgaacgtcagccagagtttggttggcaccgaaacatgctgttaccgagcgcgaagatgcaggtggcatttgtgaaagatctcgtcaccttccgc
aatccgggttcggcatacactttcgtttcatatcttcaccatgtgggacgtttggccagattcgtcaataatcaggactttttccgacccggctggaattccacgatta
cctggagtgggtggcgagccattttaccgatcgtattcgctacggagccgaagtgcgcgctattcggccagctccagctgacggttctcccggctcgctagaagt
tgccgtccgctatcgtgatcgtccaggcagtcaggtcgtctcggccgacaacgtagtgatttccactgggctcgtcccccgcgtcccggatggtaccgtactgggt
gaacgtgtttggcatagctcacaatttctcggccgcttccgggcacgtgactggtccggtgcgcgtcgcgtggccgttgtcggggcgggtcagagtgcggctgag
ttaaccgcgttttgctatgacaatacaacgaatgcggaaataatctccgttgttccgtcttatgggtactcaatcgcggatgacactccgttcgcgaacgaagtgttc
gatgggcaagcagtggatgattactatttcggtacgaaagagtcccaggatgcatattggcgctaccatcgcaacaccaactattccgttgtagatgacgacctg
ttgagagagttatatcgccgcagctacgatgatgaattcacaggtgcccgtcgtctgaatttccagcgtttgagccgtatcgatggagtagaagaaaccgcggat
ggcgcacgtattagcgtgcgctctttaagtacgggacaacgtaatcactttgacgtggactttgttgtatttgggactgggtatcatcctatggaccctggtccgctgc
tggccgaagtggatcaataccttctgcgtgatgagaacggccgctatcgggtggatcggcatcaccgtctgctgacgcgcgacggctttggcggcggtatttatc
tgcagggcggcaccgagcagacacatgggttgagtgcgtcactgctgtcgaacatggctgttcgcagcggcgaaattgtaggctcgatcctaggccgagcca
aagatggttggccgtggacggatagccgagctgcggatggcagcgaacccgcagcggccgtttcttaa

> A0A557ZX56, *Aa*PZS, *Amycolatopsis acidiphila*

atgttgttcgttcctagacagtatcgtccacctgatgtaagctggatgctgcgccttatgcgcggtaacccgttggccctcatggcaaccaatggcgatccaggcc
gggcgccacttgcgacacacttaccggttatccttgacccggaatccccgctgccagatagtgactcgctcgccggactaggtttattagctcatctgaaccgcg
cgaatccgcagtggatccaggtgaggccgggaatggaagttctgttttcctttaccggcccgcattcatacgtctcgcctgtggtctatggcctgccgaccgccgc
acctacgtgggattttaccgcagtgcaggtgcgcggtgaaattgtagagcggtttgaatctcccgaggatacgttgcgtgtggtcactgccactgtacgtgcgtat
gaacgtgatttcggcgcaggctgggacatgacgggtagcattccgtacttccgccaaattctgcccggcgttggtgccttccgcgttcgcgcgctgagtgtggag
ggtatgtttaagctgagccaggaacaaccggaagatgtgcgcgagaaagtccgtgatcacttcgcacatcgtgggcagggccgtgacctggaactggcggg
gctgatggctaacccgcgagcggcgtcagcctgcccctgtgctggtcaatttgaagaactgaaacacggggtg

> A0A2T5KZR9, *P*spNMO, *Pseudomonas* sp. GV085

atgagcactcgtggaaagatcgttgatgtactgggtattggatttggcccagcaaatatcgcgctggcaattgccctggaagaattggcgccaggcatgtccgtg
caatttcttgaaaaacgcctgagcccgctgtggcagccgaacatgctgctgccgggctcggatatccagaatcatccgctgcgagatctggtgaccccgcgca
atccgcgtagcagatacagctttacgaacttcctgttcgaacaagatcgcctgtacgagcatctgaaccttccgttgcatcatcccctgcgtgcggaatatgccca
atacgtttcttgggttgcaaatttctttgaagacatcgttgcctatggttgtgaagctgcgtcggtggaagcagtgaatccatcaaatggcgaaggcgcgcactaca
aagtgtgttgtgtcgatggcggtgtttttcttgctcgttctcttgtgctcgccccaggccgtacgcctcacattcctgcgccgttcaccgggatcaaagatcgccgcgta
cgccatttaaacgactatttgcctgccctggctgaagctttagcagccacagatgggcgctgccgcattgctgtaatcggtggcagtcagagcgcggtagagatt
ttgctgcacgcaagtgatcagactggggtaagagaggttgttggtttcacacgtaattttggttatcgccagaaagatacgagcccgttttccgacgaggtctacttt
ccgtcctttgtcgacacattccacaacgcgtctgcagaacacaaggcgcggttacgtcaggaactggtgcataccaattattcgtctgccgacatcgatgtccta
aaccagctgtatgttcgtaaatatgagcaaatgctgcaaggtgaaagtcggttgcaactgcgaactagtgaggacatggtcggttgcatacctaccccgaacg
gtgtgcagataaaaagtcggcagtatcttcaaggaagcattcatagccagtcgttcgatttagtgattctggccacgggttttctcgatctcggcaccggagatcgt
caggagccctttccgtccttactagccccaattgcccgtgaaaatgaaattgacacgctgaccattgcgcgcgattatcgcgtcgacttgctcaacggcttgccca
cctacctcaacgggttatgcgagtcttcacacggcatgggggacgcgggctcgttctccctgctggcgttacgttcacagtcaatcgtggaatcactgaccagca
acctggaagcaagggtgcagcgccatgct

> A0A2T5L023, *P*spPZS, *Pseudomonas* sp. GV085

atgccggcctgccccttttcattatcctcagtatcgttccactgaccagggtatgatcgaccgttttattgatgtgtttccgctggcaatgatcacatcgcatcgggatgg
acaatttctttgtagccacattccgctgtggcgtcagccggatgggtcgttgttcggtcatgttgatggcaataatagtcagttcaagggcgaacagtatctgagtgc
ccaaatcgtcttcatgggcccatcggcttacattcctccacaggcgtacgtaagccgccaactcccgacttggaattacctggccgttcacatgcaggcgaacat
tacggtggtggatgccccccaccagaaattggaaatcctggaacaaaccgcgaaacgattatcagagcagccgtctgactatcaggtcgacgaacgcgatc
ctagagtaataagcaacctgccgcatattcttggtttggtgattcagccggaaaataccgaaggccgttttaaactctcccaagacaaaacgtctccagatagca
gtgcggcgctgcgctggttactagataaccgcaagcacgatcatgcagagttcctgatggagctgctgcacgctgcaaccaaaccgcattcaaac

> A0A4R2JQS9, *Aw*PZS, *Actinocrispum wychmicini*

gtgaacacgtatctgaccgagcggtacgcgacccgcgacccggacaggatcctggcgttcgtcgcggagttcccgttcgccacgctgatcccgcagggcga
ggtgcgcggtgccctgccgtcactcccggtgttgttggacgtgcgagccggctcgcggatcgtgttctacggccacctggaccgcgagaacccgttcgcggtgc
ggttggaccgcagccgggtgaccgcgctcttccaggggccgaacggctacatctcgccgcgggactacgtctcacggcagttcccgacctggaactacgcg
gtggcccaggtgaccggcgtctgccggctcgtcgacgagcccgaccggaagctggcctacatgatccggatggtcgaggacctggagcggcacaacggtt
cggactaccggctggacggatccgacaaccgggtccgccggcagatcgacctggtgaccttcttccagctcgaggtcgactcggtgcacggcacgttcaagtt
cgcccaggagaagaccgtcgaggaccgcgtccgagcacgcgacagactcattgacaagttgcacagcggccagagccgggcgatacccgtgctcgccg
acctggacccaggagagcggccatga

> A0A5S4H6S6, *Ag*NMO, *Actinomadura geliboluensis*

atgtatgatgtgattgtcattggtgcacgttgcgcgggttcgccggcggccatgttgtttgcaagacaaggttatcgcgttctgttactggaaaagcgcgttttcctc
aagatacgctgtccagccattacattcaccagccgggcgtggcattacttgccagatgggatctgcttggcgaattacgccgcgcgggctgtcggccgatcgac
catgagagctatgatgggccaggcgttcgcctggacggctttagtctgccggttgatggtcatcgtactacgtacgctccacgccgttttgttttagacccgattctgg
ccggcggtgcagtcgctgccggggcggagtttcaggagagttgtgctgttaccgatctgctgcacgaaggcggccgtgtagccggtgtacgttatacgactccg
ggtggcgccgaagcgaccgaaagggctcgtctggtcgtcggcgcggatggcatgcgttctctggtggcgcgcaaaacccgagcgccgaacgttattgaaca
tccgcggatgacctgcgtctattatagctactggtccggtgtacctgcgcacatggaactgtacgagcgacccgggcgctgggtagtcgccatcccaaccaacg
atgacctcacactgataatgacatatttcccgcaggacgaatttgccaaggtacgtaccgcggttgaaccgagttacctcggagcattacgcactacggcgcct
gagctgtacgatcggatgtcggcaggcgcgcgcgtggaacagatgtatggcactggtcatcaggagaacttctttcgcaaagcacatggtcccggatgggtgc
tgcttggggacgcgctgcaccataaggactcaatcaccgcccgcggtatcaccgatgccttccttcaggcccagtcgttaacagaccatatcgggcagggtctg
cacgacgatgcagctctcgatgcggctctgaatcgcttcgaaaacgacctgaatgatgacttcttggatctatatcagggagtgctcaatgtggcagagttgaaa
ccagaagggcgtaccgaaatgttgcggaaactggtgggacaccaagaactgattgatcgttacttttcaacggtgtctggcgcttgcagctttgatgatttctataa
tgccgaacttttgacggtgctagatcaaagctaa

> A0A5S4H7I3, *Ag*PZS, *Actinomadura geliboluensis*

atgttcgtccccagtcactaccgcgaaccggatgtctcatggatggttgatctgatgcgcgggaacccgttggcactgatggccagcaatggtactccagctgat
ggtccgtttgcaacacatttaccggttattactgacccacaatgggagggctcaccaacggcggatctggcgggcatgcctctgctgggtcacatgaaccgtgc
caatccgcattgggcggcgttagaaaccgggtctgcgatcctgctgacgtttaccggtccgcatgcctacgtctcccccacagtctatgatgttacgcctgccgcc
ccgacatggaactttacctcggtgcatgtgcacggagtggttgagaaaatcgaatcgacggaggaaaccttggatgttgtacaggcgaccgtgcaagcattcg
aaggcgaattcggcgactcctgggacatgagcgaatctgttgactattttcgcaagattgtgactggcgtcggcgcttttcgtgtacgagtgaccaaagcagagg
gaatgtttaaactcagccaggaacagcgtcctgaaattcgcgagagagtggtgcagagtttcgcgggtcgcgaatgcacccggcatgtacagacggctgatct
aatgaatcgtcttccg

> A0A372GFN1, AspPZS, *Actinomadura* sp. LHW52907

atgtttgttcctcatgcataccgcgaaccagatgaatcatggatgcatgacctggtccatggtaatccgctggctcaattggttagtaacggcaaaggtccggaac
cgccttggatgactcatgtcccgatcattgttgatccgcacgcgagccgtcccccgggcgatctgagtggacttacgttgtggggccacatgaatagagagaac
ccgcattggagcgcactgggcacggccgctcccgtggttgtagcgtttaccggtccgcatgcttatgtgtcgccagccgtgtacggatctacgccaaccgcgcc
cacctgggattttaccgcagtccacgcgcacggcactctgcataaagtagacagcgcggatgaaacgctggcgacggttctagagaccgtacgtacattcga
aagccggtttggggccggctggtccatgactgatagtctcgggtatttccgtcgcatcctgcctggtgtgggtgcatttcgggtggcggtcacccgtgccgacggta
tgttcaaattaagccaggaacagtctccgcagattcgcgggcgtgtgcgtgacgcgttcgctcaacgagcgtgcacccaccatcgcgcaattgcgtcgctgatg
gatcgcttagagccaaaggccggcgatctcgccgaagcctgttcctcccctgcaccgccgtcacttccgcgcacagac

> A0A222TEN0, GorA, *Gordonia rubripertincta CWB2*

atgggccatcatcatcatcatcatcatcatcatcacagcagcggccatatcgaaggtcgtcatatgacccgcgttgaaaacgtggatgtactggctatcggctgtg
gtccgtttaacctgggtttggctgcgctggcgagtaccgtcgatgatctcgacgtgctcgttgtggacagccgcgaagaatttcgctggcatcctggcctgatgtttg
acgaagcacgtttacaagtaggctttctgagcgatctggtgacgctggttgatcccactcatccgatgtcattcctgaattacatggcggacaccgaccggatgta
tcgcttcctggtccgtgaaaacttctacccgacacggattgagtatgaggcttatctgcattggtgcatcgatcgcttggattcgctccgctggggtaccaccgtgac
ggaggttagctgggatgacaacgcagacgcgtttgcagtaaccgtagtcacaggaggtaccccgtcaaccgtgattgcccgccatgtggtagtgggcgttggg
acggagccgctggtgcctgaatcgctgagctcagattcgccggctgtggtccactctagtgactatctttatcaccaggacaaagcacatgcggccgatactgtg
accgttatcggtagtggtcaatccggcgctgagattgtgatcgatctgcttgaagccaatctgcgtggcggcccgtccgttcgttggtggacccgcactccatggttt
gcaccgctggattttaccaaaatgagcttggagatgaccaccccggcatacatggactacttccagtccttgcctgaagaagcgcgtgatcgcattcgcccgca
acactggcagttccacaaaggggtcagttcggacacgttggaacgggttcacgaacttatgtatcagcgtcaactgcgtgacaagttaaatcccgttcagttacg
tatcagcacggaagttgacgggattgatactcttgccgatggccgcttaaaagtgcgtggccgtcacctggatactgggacgcaactggcgcatactaccgac
atggtcatcgcgtgcacgggttaccagccacgtccaatgccttttctggccccgattgaatctcagctccatcgtgatagccgcggacgcttagtggtcggtgccg
cacatcaggtcgaaacagaaccagccttagcgaatcgcttgttcgttgccaacgccgaggaacatgcagtcggcgtatctgcgcccaatctggatattggcgc
cgtacgtaacgctcgcattctgaacgcggtcacgggacgcgaagtgtatcgcctgccgaaagatacggcgtttacagcgttcggtgttgatgatctggatgatgt
ggtgggttga

3. Supporting figures and tables

Table S1. Overview of selected NMOs and corresponding PZSs.

NMO (Uniprot ID)	NMO name	PZS (Uniprot ID)	PZS name	Identity to KtzT (%)***	Organism	Piz-containing compound(s)	Ref.
A8CF85	KtzI*	A8CF72	KtzT	-	*Kutzneria* sp. (strain 744)	Kutzneride 2	[4]
A0A1B1AU19	*Sg*NMO	A0A1B1AU73	*Sg*PZS	59	*Streptomyces griseochromogenes*	Unknown	-
A0A5N8XG21	*Ss*NMO	A0A5N8XFV2	*Ss*PZS	56	*Streptomyces spongiae*	Piperidamycins	[5, 6]
U5YL02	*S*spNMO	U5YN79	*S*spPZS	51	*Streptomyces* sp. RJA2928	Padanamides	[7]
A0A1U9K2D1	*S*spMNMO**	A0A1V9K2B3	*S*spMPZS	57	*Streptomyces* sp. M41	Dentigerumycins	[8]
A0A2S8QH87	*Pl*NMO	A0A2S8QH93	*Pl*PZS**	26	*Photorhabdus luminescens*	Unknown	-
A0A557ZX73	*Aa*NMO	A0A557ZX56	*Aa*PZS**	48	*Amycolatopsis acidiphila*	Unknown	-
A0A2T5KZR9	*P*spNMO**	A0A2T5L023	*P*spPZS	25	*Pseudomonas* sp. GV085	Unknown	-
A0A4R2JQT7	*Aw*NMO*	A0A4R2JQS9	*Aw*PZS	34	*Actinocrispum wychmicini*	Unknown	-
A0A5S4H6S6	*Ag*NMO**	A0A5S4H7I3	*Ag*PZS	52	*Actinomadura geliboluensis*	Unknown	-
-	-	A0A372GFN1	*A*spPZS	65	*Actinomadura* sp. LHW52907	Unknown	-
A0A222TEN0	GorA	-	-	-	*Gordonia rubripertincta* CWB2	-	[3]

***PZS sequence identity % to KtzT determined by blastp
**Proteins did not express in the soluble fraction or unstable
*NMO was not used in this work

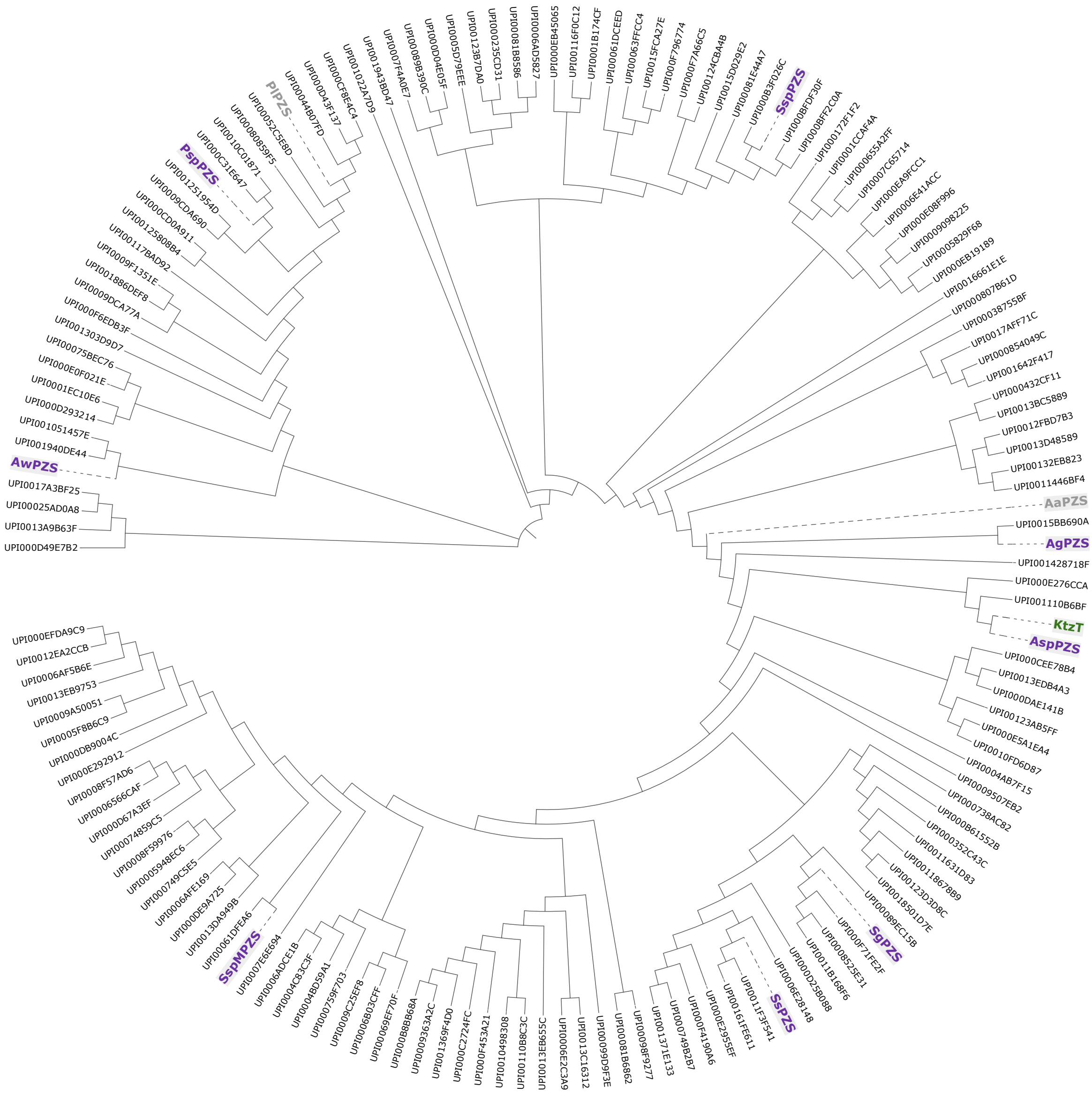

Figure S1. Neighbour-joining phylogenetic tree of 155 PZSs with enzymes used in this study highlighted in bold. The evolutionary history was inferred using the Neighbor-Joining method.[10] The optimal tree with the sum of branch length = 32.830 is shown. The confidence probability (multiplied by 100) that the interior branch length is greater than 0, as estimated using the bootstrap test (100 replicates are shown).[11,12] The evolutionary distances were computed using the JTT matrix-based method[13] and are in the units of the number of amino acid substitutions per site. The analytical procedure encompassed 155 amino acid sequences. The pairwise deletion option was applied to all ambiguous positions for each sequence pair resulting in a final data set comprising 2.342 positions. Evolutionary analyses were conducted in MEGA12[14] utilizing up to 7 parallel computing threads. The tree was visualized using iTOL.

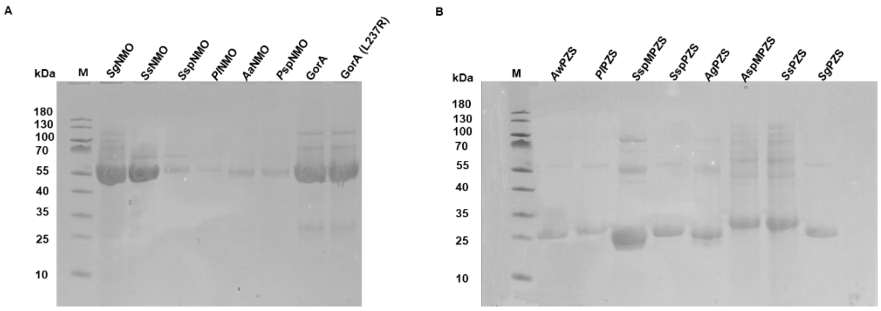

Figure S2. (A) SDS-PAGE of selected NMOs. The molecular weight for each protein: *N*-His6-*Sg*NMO (50.51 kDa), *N*-His6-*Ss*NMO (48.52 kDa), *N*-His6-*S*spNMO (49.39 kDa), *N*-His6-*Pl*NMO (49.08 kDa), *N*-His6-*Aa*NMO (49.62 kDa), *N*-His6-*P*spMO (50.14 kDa), GorA and GorA (L237R) (51.36 kDa). (B) SDS-PAGE of selected PZSs. The molecular weight for each protein: *C*-His6-*Aw*PZS (26.34 kDa), *C*-His6-*Pl*PZS (29.36 kDa), *C*-His6-*S*spPZS (25.46 kDa), *C*-His6-*Ag*PZS (25.83 kDa), *C*-His6-*A*spPZS (27.73 kDa), *C*-His6-*Ss*PZS (27.21 kDa), *C*-His6-*Sg*PZS (25.83 kDa). Electrophoresis was performed for 2 h at 120 V and room temperature. Gels were stained overnight in a colloidal Coomassie staining solution and afterwards washed with water. PageRuler™ Prestained Protein Ladder, 10 – 180 kDa by Thermo Scientific, was used as a size standard marker (M).

Genomic context of selected genes

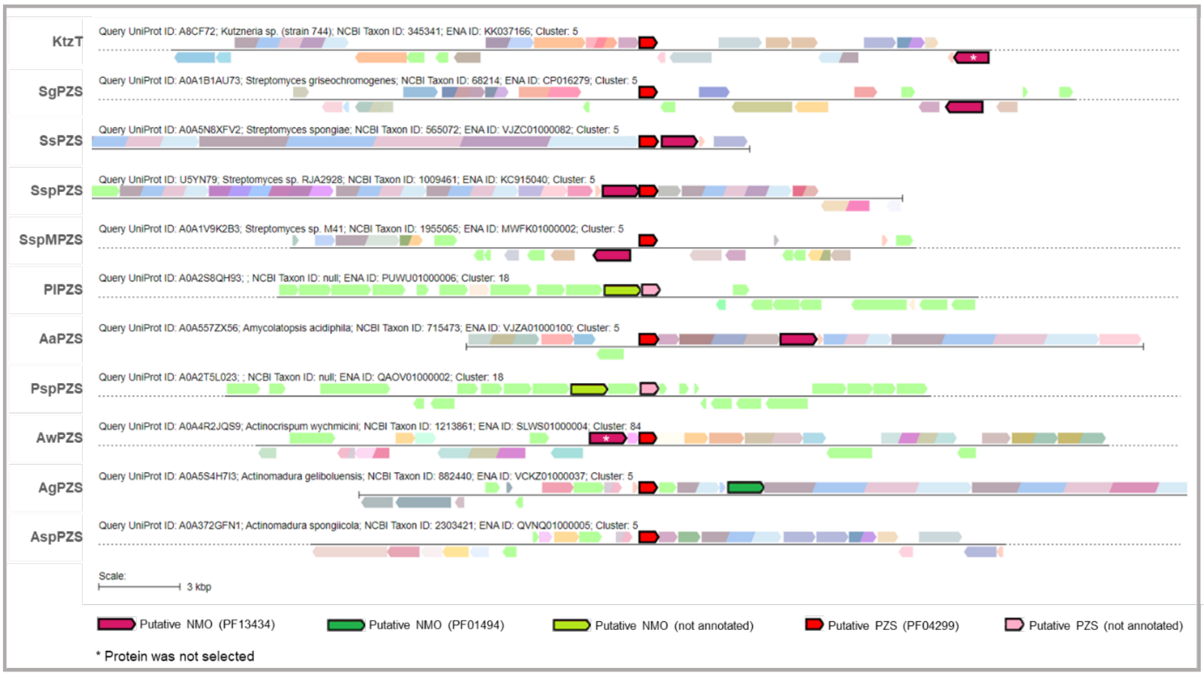

Figure S3. Gene neighborhood plots of representative putative PZSs. NRPKS/PKS and *N*-hydroxylation monooxygenases are commonly neighbors with putative PZSs.

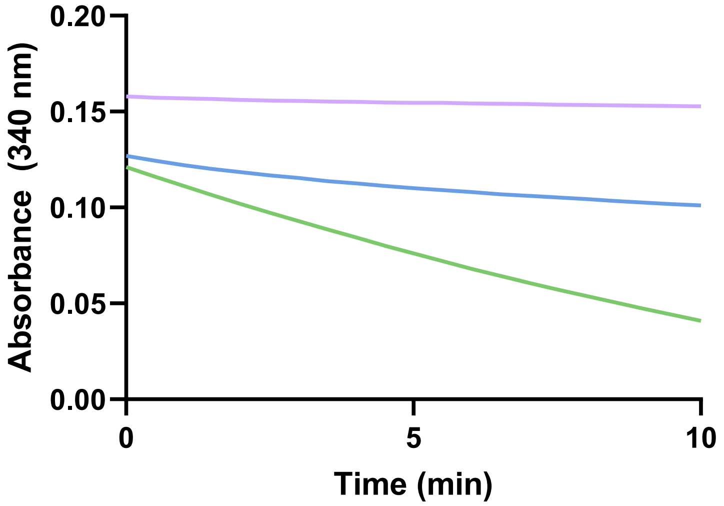

Figure S4. Spectrophotometric-based time course measurement of NADPH to determine O_2 uncoupling in *Ss*NMO-catalyzed transformation. Reaction conditions: 200 μL reaction volume containing 0.5 mM NADPH, 1 mM **5d** and 50 mM NaPi buffer pH 7.4. The reaction was initiated by adding 10 μM *Ss*NMO. The whole reaction is shown in green, and controls in absence of either **5d** (blue), or **5d** and *Ss*NMO (purple).

4. Evaluation of substrate promiscuity of NMOs

The activity of NMOs was assessed under the following conditions: In a 100 μL volume, 30 μM NMO, 10 mM glucose, 1 mM substrate (**9e** – **9r**), 10 U/mL GDH, 1 mM $NADP^+$, 1 mg/mL catalase, 50 mM NaPi buffer pH 7.4 were mixed and incubated at 25 °C for 1 h. The amount of hydroxylated product was determined by the Csáky assay. Data was obtained from triplicate measurements against a calibration curve with hydroxylamine.

Table S2. Conversion of selected NMOs towards the substrate panel **9e – 9n, 5a – 5d**.

#	Substrates (9)	Putative *N*-hydroxylated product (10)	Quantitative -NHOH yield (%)						
			*Sg*NMO	*Ss*NMO	*Ssp*NMO	*Pl*NMO	*Aa*NMO	GorA	GorA (L237R)
**9a**	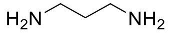	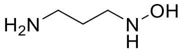	ND	ND	ND	ND	ND	5	2
**9b**	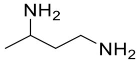	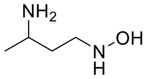	3	ND	ND	ND	ND	3	10
**9c**	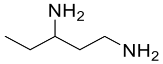	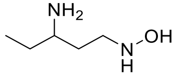	ND	ND	ND	ND	ND	5	4
**9d**	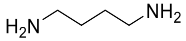	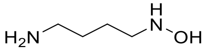	ND	ND	ND	ND	ND	93	89
**9e**	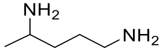	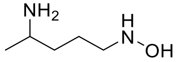	15	ND	ND	ND	ND	ND	ND
**9f**	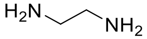	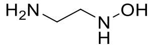	ND	ND	ND	7	14	ND	20
**9g**	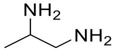	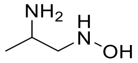	ND	ND	ND	6	10	ND	2
**9h**	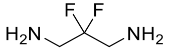	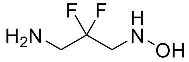	23	ND	ND	ND	ND	ND	ND
**9i**	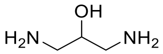	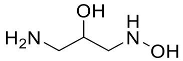	ND	ND	21	ND	ND	ND	ND
**9j**	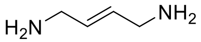	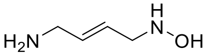	ND	ND	ND	ND	ND	2	ND
**9k**	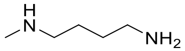	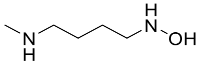	ND	ND	ND	ND	ND	17	ND
**9l**	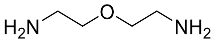	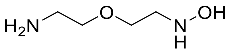	ND	ND	ND	ND	ND	30	ND
**9m**	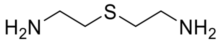	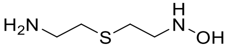	ND	ND	ND	ND	ND	34	ND
**9n**	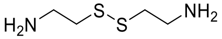	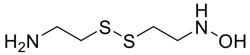	ND	ND	ND	ND	ND	19	ND

**5a**	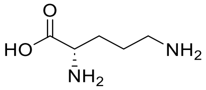	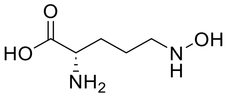	82	80	ND	ND	ND	ND	ND
**5b**	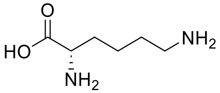	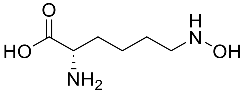	ND	ND	ND	ND	ND	ND	ND
**5c**	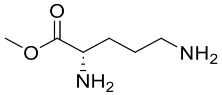	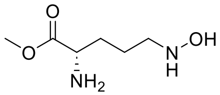	ND	5	ND	ND	ND	ND	ND
**5d**	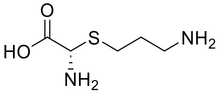	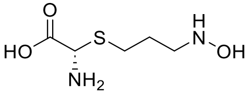	ND	42	ND	ND	ND	ND	ND

ND: not detected.

Table S3. PZS promiscuity against substrate panel obtained from the NMO-catalyzed *N*-hydroxylation using substrate **9a – 9n** and **5a – 5d**. Reaction conditions: 100 µL reaction volume containing 20 mM NH_4HCO_3 pH 8.5, 1 mM substrate (**9a – 9n**, **5a – 5d**), 1 mM $NADP^+$, 0.05 mM FAD, 10 mM glucose, 10 U/mL GDH, 1 mg/mL catalase, 30 µM NMOs and 5 µM heme-containing PZS, 100 µL reaction volume, incubation at 25°C, 1 h. Extract ion chromatography (EIC) of expected mono/bis-Fmoc were integrated, assuming the ionization of all compounds are the same. Neg. Ctrl.: control without the addition of PZS.

#	Substrates (9)		EIC									
		N-N-bonded product (11)	Neg. Ctrl.	KtzT	*Aw*PZS	*Psp*PZS	*Ssp*MPZS	*Ssp*PZS	*Ag*PZS	*Asp*PZS	*Ss*PZS	*Sg*PZS
**9a**	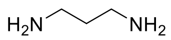	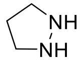	ND	3.3×10^5	4.6×10^4	ND	1.1×10^5	ND	1.3×10^5	ND	ND	9.7×10^3
**9b**	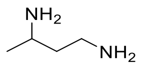	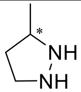	ND	1.8×10^6	5.9×10^5	2.4×10^6	3.0×10^6	9.6×10^4	2.2×10^6	5.4×10^5	1.0×10^6	2.0×10^6
**9c**	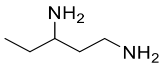	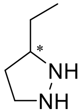	ND	5.6×10^5	9.2×10^4	2.0×10^5	2.1×10^5	4.6×10^5	3.7×10^5	ND	2.0×10^5	2.0×10^5
**9d**	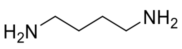	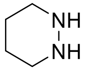	ND	1.7×10^5	2.0×10^4	2.5×10^4	2.8×10^5	7.4×10^4	1.0×10^5	3.4×10^5	8.9×10^4	2.0×10^4
**9e**	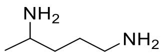	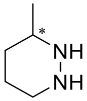	ND	6.2×10^5	1.7×10^4	2.1×10^5	1.3×10^6	ND	4.6×10^5	6.2×10^4	5.7×10^5	3.8×10^5
**9f**	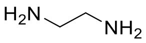	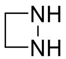	ND	ND	ND	ND	ND	ND	ND	ND	ND	ND
**9g**	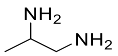	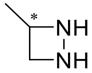	ND	ND	ND	ND	ND	ND	ND	ND	ND	ND
**9h**	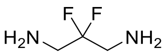	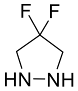	ND	ND	ND	ND	ND	ND	ND	ND	ND	ND
**9i**	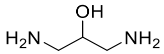	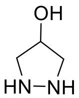	ND	ND	ND	ND	ND	ND	ND	ND	ND	ND
**9j**	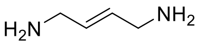	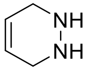	ND	ND	ND	ND	ND	ND	ND	ND	ND	ND

**9k**	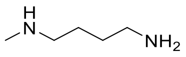	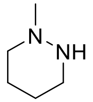	ND	ND	ND	ND	ND	ND	ND	ND	ND	ND
**9l**	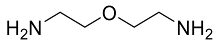	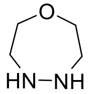	ND	ND	ND	ND	ND	ND	ND	ND	ND	ND
**9m**	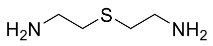	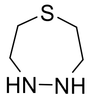	ND	ND	ND	ND	ND	ND	ND	ND	ND	ND
**9n**	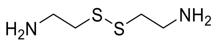	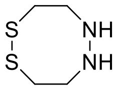	ND	ND	ND	ND	ND	ND	ND	ND	ND	ND
**5a**	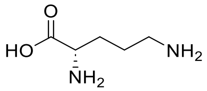	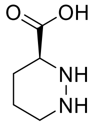	ND	3.5×10^{6}	ND	ND	2.0×10^{6}	8.4×10^{5}	1.5×10^{6}	ND	2.5×10^{6}	9.7×10^{5}
**5b**	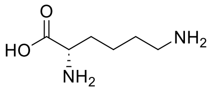	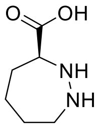	ND	ND	ND	ND	ND	ND	ND	ND	ND	ND
**5c**	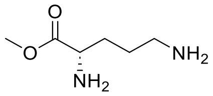	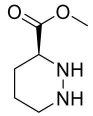	ND	ND	ND	ND	ND	ND	ND	ND	ND	ND
**5d**	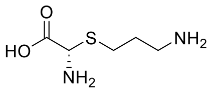	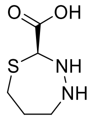	ND	ND	ND	ND	ND	ND	ND	ND	ND	ND

ND: not detecte

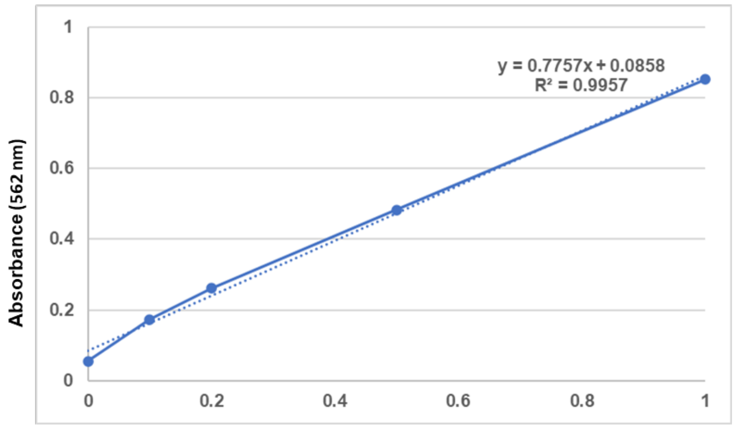

Figure S5. Calibration curve of hydroxylamine phosphate (concentration ranging from 0 – 1 mM) used for the Csáky assay. The data derives from triplicate measurements.

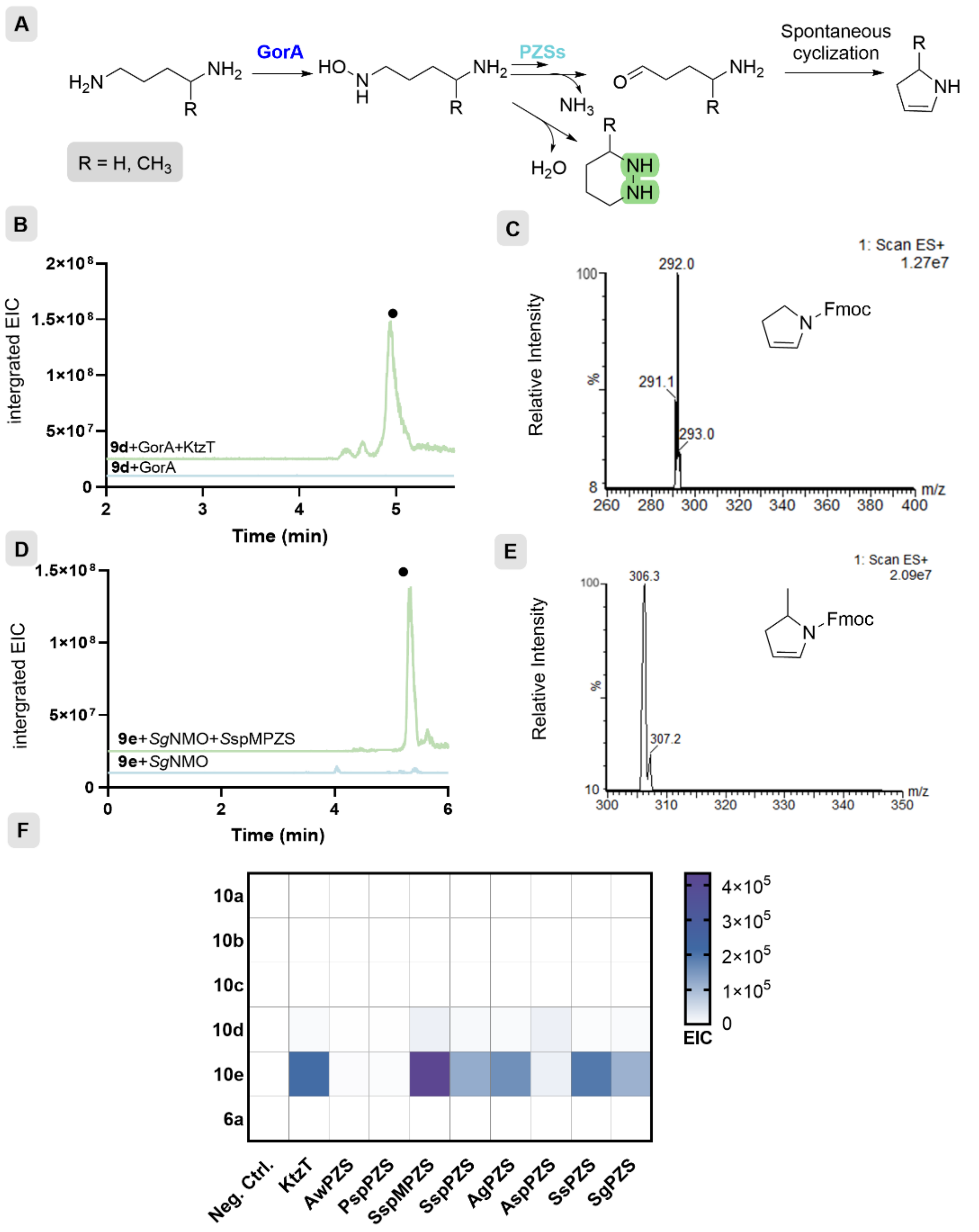

Figure S6. (A) Overview of the proposed side reaction of PZS. (B) The enamine formation occurs alongside the desired N-N-bond-containing product **11d** when KtzT was added to the reaction (green), starting with **9d** as substrate. (C) The corresponding spectrum of the proposed side product with Fmoc-Cl derivatization while forming **11d**, found $[M+H]^+$ m/z = 292.0, theoretical $[M+H]^+$ m/z = 292.1338, calculated for $C_{19}H_{17}NO_2$. (D) The deamination product was formed by adding *S*spMPZS and substrate **9e** (green). (E) The corresponding spectrum of the proposed deamination product with Fmoc-Cl derivatization while forming **11e**, found $[M+H]^+$ m/z = 306.3, theoretical $[M+H]^+$ m/z = 306.1494, calculated for $C_{20}H_{19}NO_2$. (F) Side reaction analysis of PZS homologs against substrate panel **10a** – **10e** and **6a.** Reaction conditions: 20 mM NH_4HCO_3 pH 8.5, 1 mM substrate (**9a** – **9e**, **5a**), 1 mM $NADP^+$, 0.05 mM FAD, 10 mM glucose, 10 U/mL GDH, 1 mg/mL catalase, 30 µM NMOs and 5 µM heme-containing PZS, 100 µL reaction volume, incubation at 25 °C, 1 h. Extract ion chromatography (EIC) of expected mono-Fmoc was integrated. Neg. Ctrl.: control without the addition of PZS.

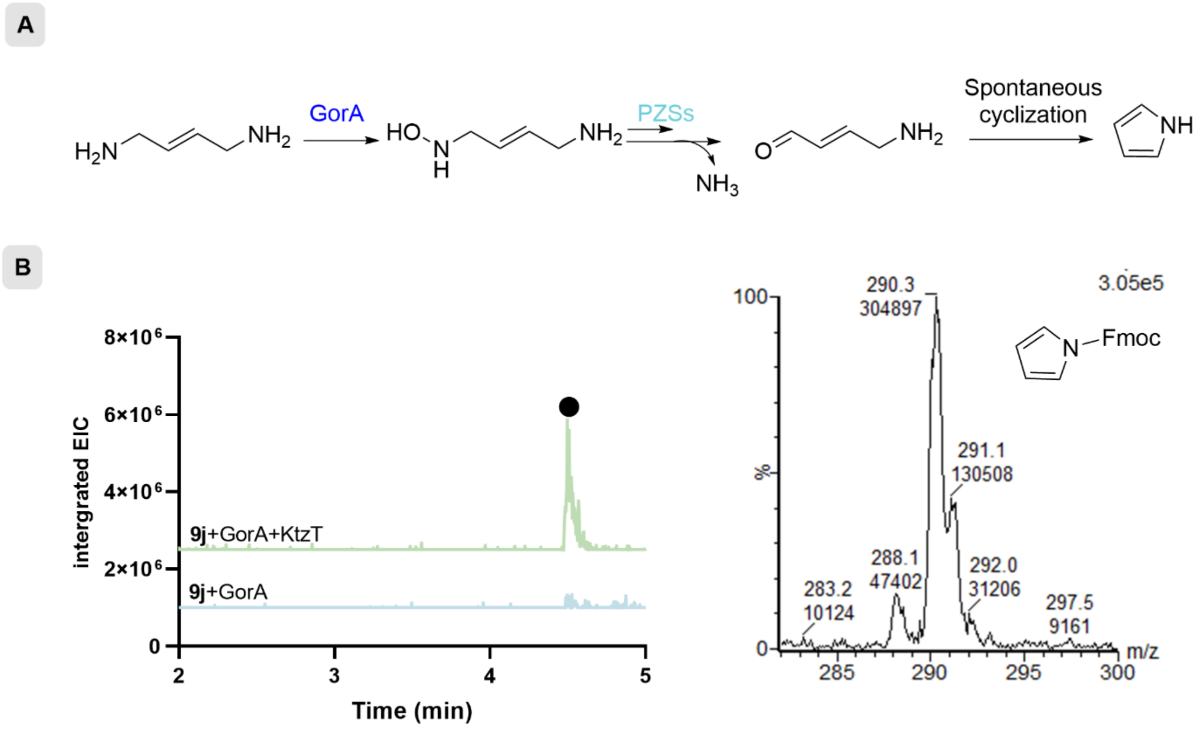

Figure S7. (A) Overview of the proposed side reaction of PZS. (B) The *H*-pyrrole formation occurs when KtzT was added to the reaction (green), starting with **9j** as substrate. (C) The corresponding spectrum of the proposed side product with Fmoc-Cl derivatization, found $[M+H]^+$ m/z = 290.3, theoretical $[M+H]^+$ m/z = 290.1136, calculated for $C_{19}H_{15}NO_2$.

5. Characterization of the coupled cascade

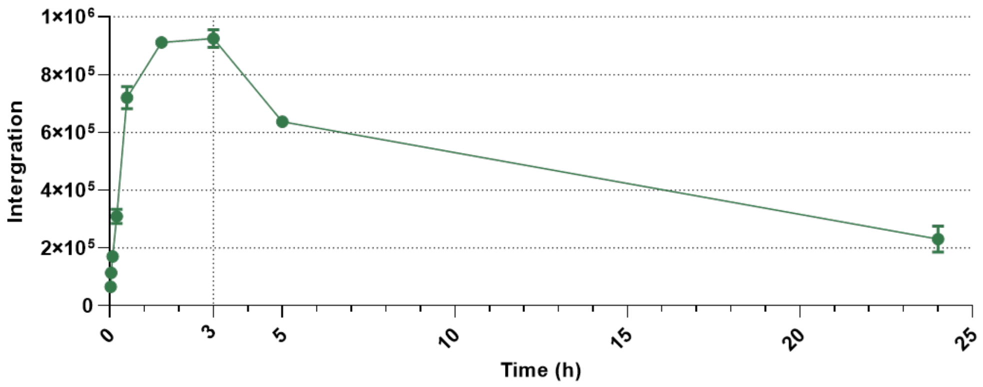

Figure S8. Time course experiment for the formation of **11a** in the dual cascade with KtzT and GorA. The assay mixtures contain 20 mM NH_4HCO_3 buffer pH 8.5, 1 mM $NADP^+$, 0.05 mM FAD, 1 mg/mL catalase, 10 mM glucose, 10 U/mL GDH and 1 mM **9a**, the reaction was initiated by adding 30 μM GorA and 2 μM KtzT. The reaction mix was incubated at 25 °C in a total reaction volume of 100 μL. Samples were analyzed by HPLC by integrating the area for Bis-Fmoc-**11a.** Results are derived from triplicate measurements.

6. HPLC traces of all compounds

Semi-preparative HPLC data

General reaction conditions for the one-pot cascade: in a total volume of 15 mL, 2 mM substrate (**9a – 9e**), 1 mM NADP$^+$, 0.05 mM FAD, 10 mM glucose, 10 U/mL GDH, 1 mg/mL catalase, 50 μM NMOs and 2 μM heme-containing PZSs were mixed. After the reaction was completed, 4 equivalents of Fmoc-Cl (31 mg) were added to the mixture and stirred at 300 rpm for 2 h at room temperature. 20 μL was withdrawn and injected to HPLC.

Column: Phenomenex Luna C18 column (4.6 × 250 mm, 5 μm) at 30 °C. The elution was performed at 1 mL/min with a mobile-phase mixture containing of a linear gradient of water and acetonitrile ((v/v): 30:70 to 10:90, 0 to 20 min; 10:90, 20 to 30 min), both of which contain 0.1% (v/v) trifluoroacetic acid (detection wavelength: 254 nm).

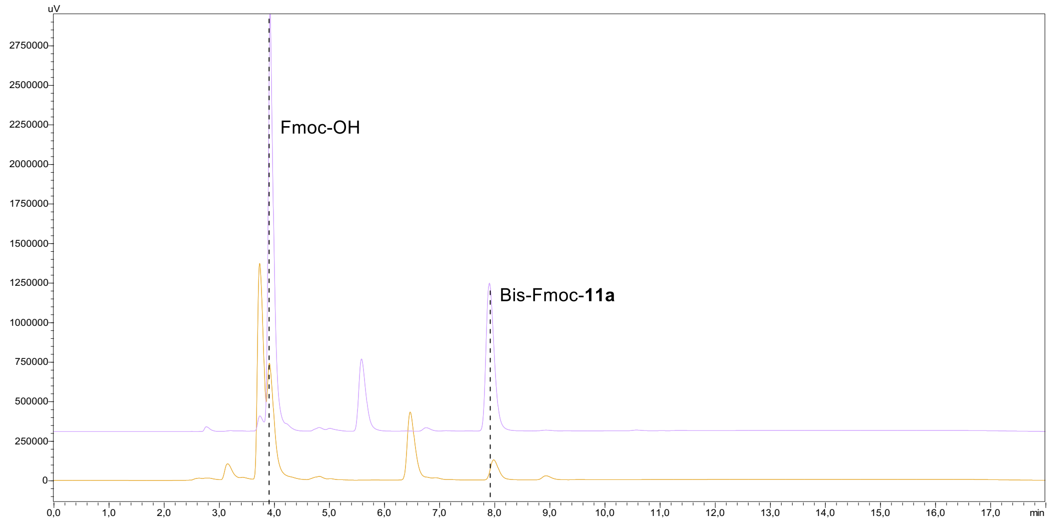

Figure S9. HPLC chromatograms of the trace of Bis-Fmoc-**11a** obtained from the GorA-KtzT coupled reaction with Fmoc-Cl derivatization. Reference compound Bis-Fmoc-**11a** is displayed in purple; the whole cascade is shown in orange. Fmoc-OH is a hydroxylated product of Fmoc-Cl.

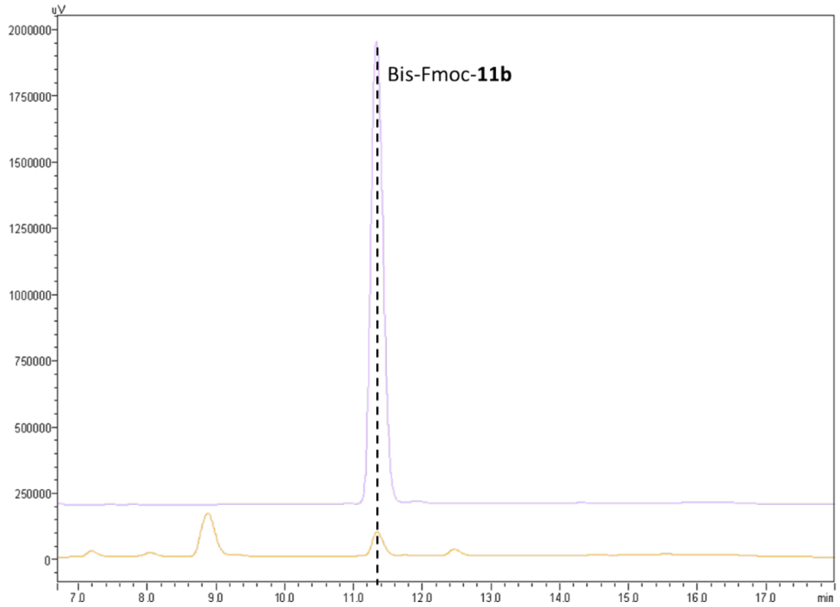

Figure S10. HPLC chromatograms of the trace of Bis-Fmoc-**11b** obtained from the GorA (L237R)-*S*spMPZS coupled reaction with Fmoc-Cl derivatization. Reference compound Bis-Fmoc-**11b** is displayed in purple; the whole cascade is shown in orange.

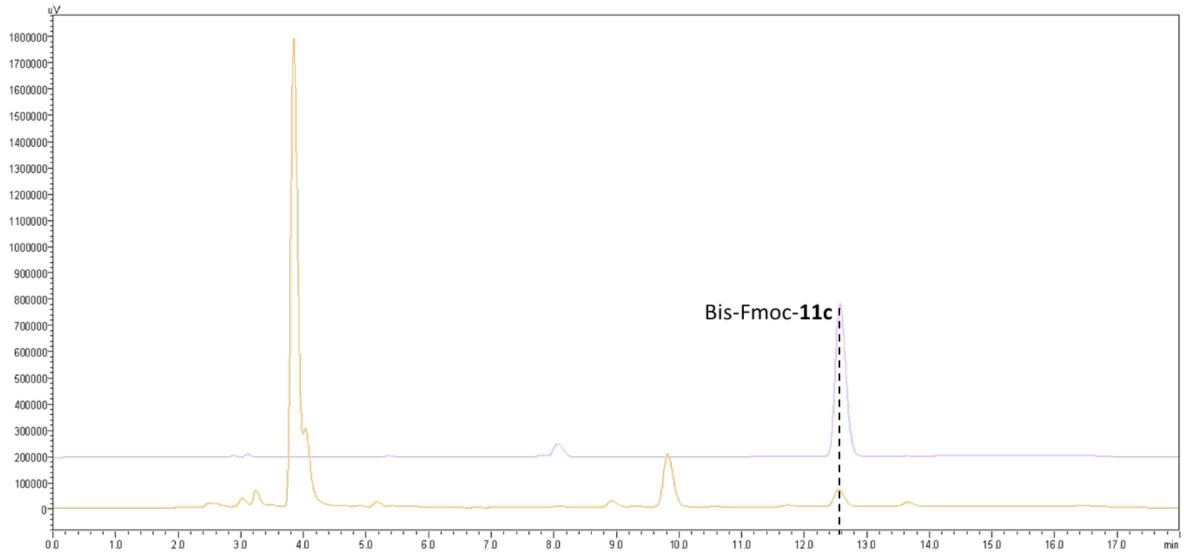

Figure S11. HPLC chromatograms of the trace of Bis-Fmoc-**11c** obtained from the GorA-KtzT coupled reaction with Fmoc-Cl derivatization. Reference compound Bis-Fmoc-**11c** is displayed in purple; the whole cascade is shown in orange.

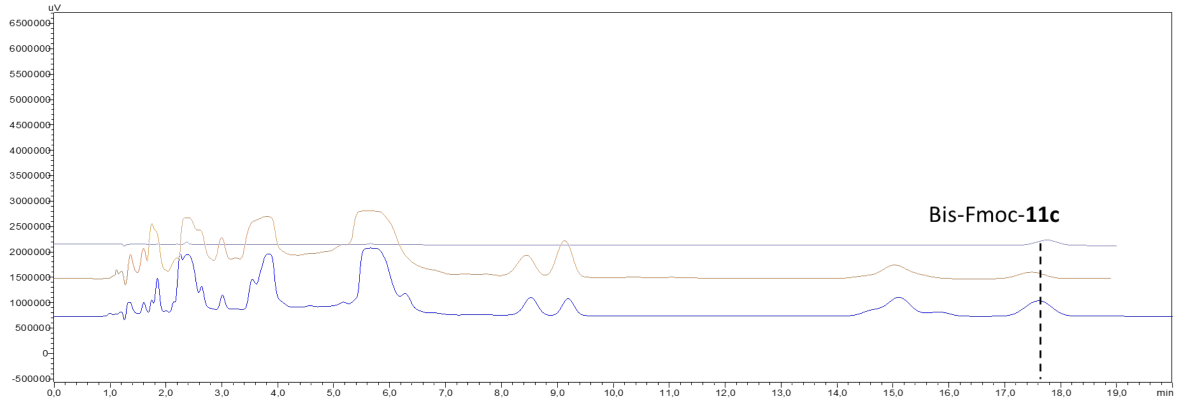

Figure S12. HPLC chromatograms of the trace of Bis-Fmoc-**11c** obtained from the GorA-*S*spMPZS/*S*spPZS coupled reaction with Fmoc-Cl derivatization. Reference compound Bis-Fmoc-**11c** is displayed in purple; the whole cascade GorA-*S*spMPZS is shown in orange. GorA-*S*spPZS cascade is displayed in bule.

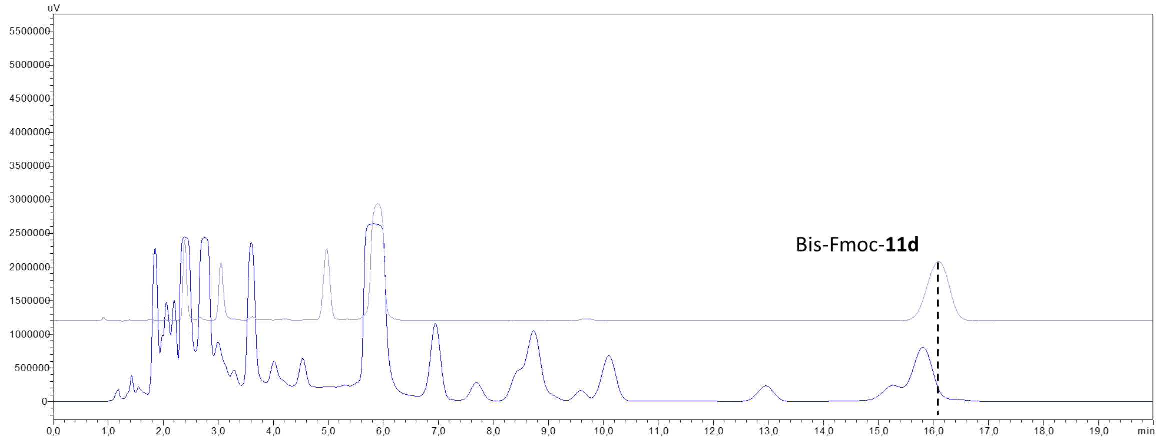

Figure S13. HPLC chromatograms of the trace of Bis-Fmoc-**11d** obtained from the GorA-*A*spPZS coupled reaction with Fmoc-Cl derivatization. Reference compound Bis-Fmoc-**11d is** displayed in purple; the whole cascade is shown in blue.

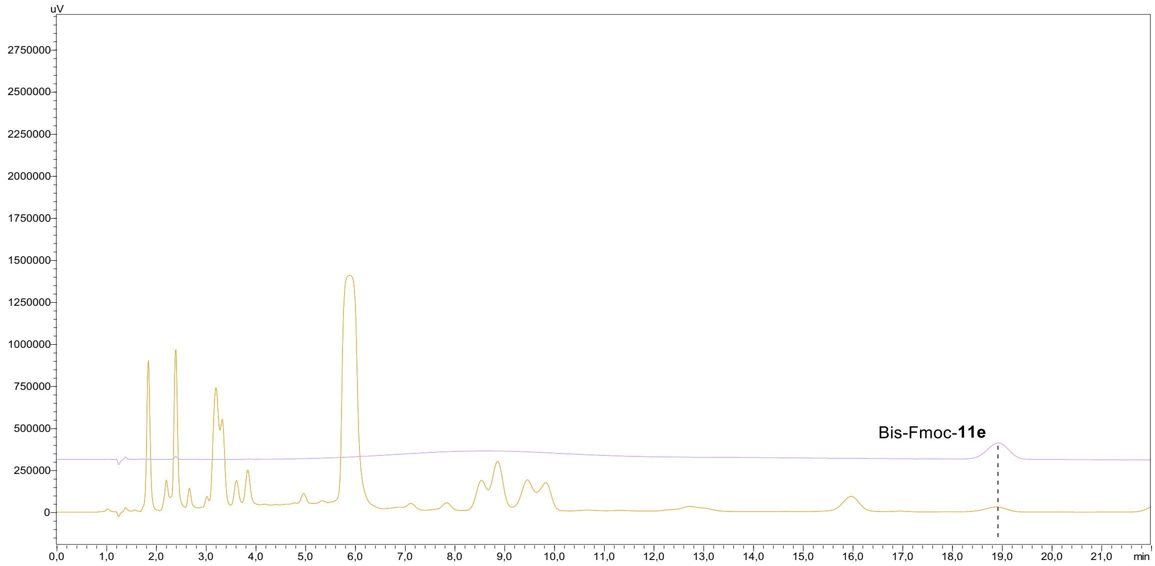

Figure S14. HPLC chromatograms of the trace of Bis-Fmoc-**11e** obtained from the *Sg*NMO-*S*spMPZS coupled reaction with Fmoc-Cl derivatization. Reference compound Bis-Fmoc-**11e** is displayed in purple; the whole cascade is shown in orange.

Chiral HPLC data

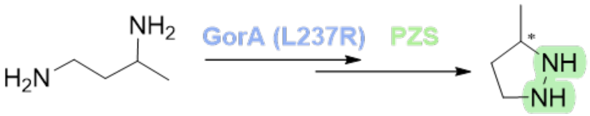

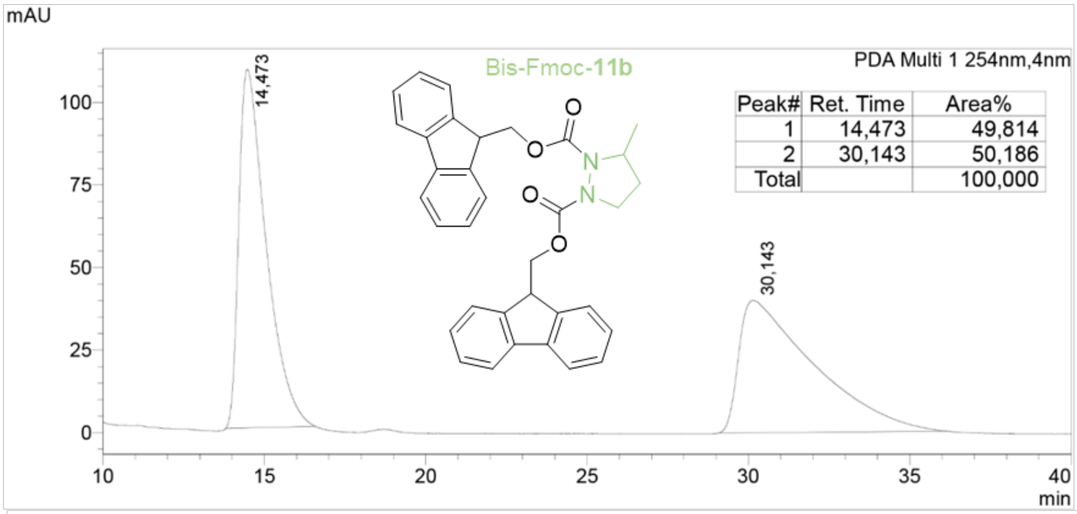

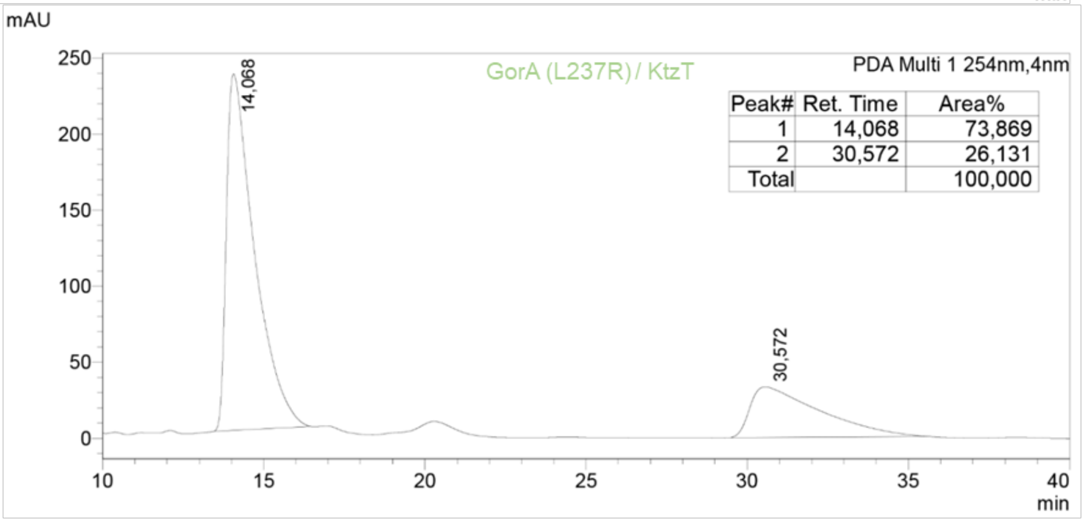

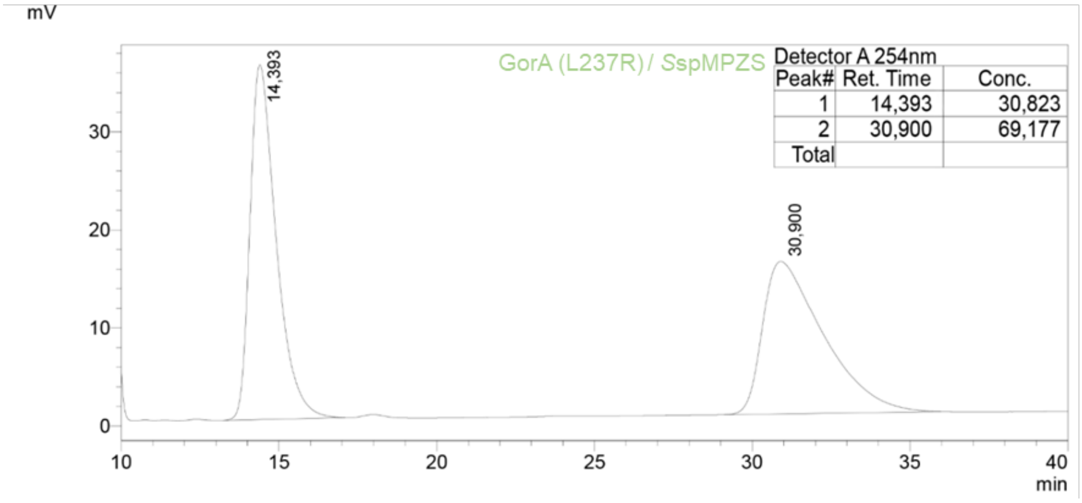

Figure S15. Chiral HPLC chromatogram of racemic reference and enzymatically synthesized Bis-Fmoc-**11b** (from top to bottom) obtained from the coupled reactions of GorA (L237R)/KtzT and GorA (L237R)/*S*spMPZS.

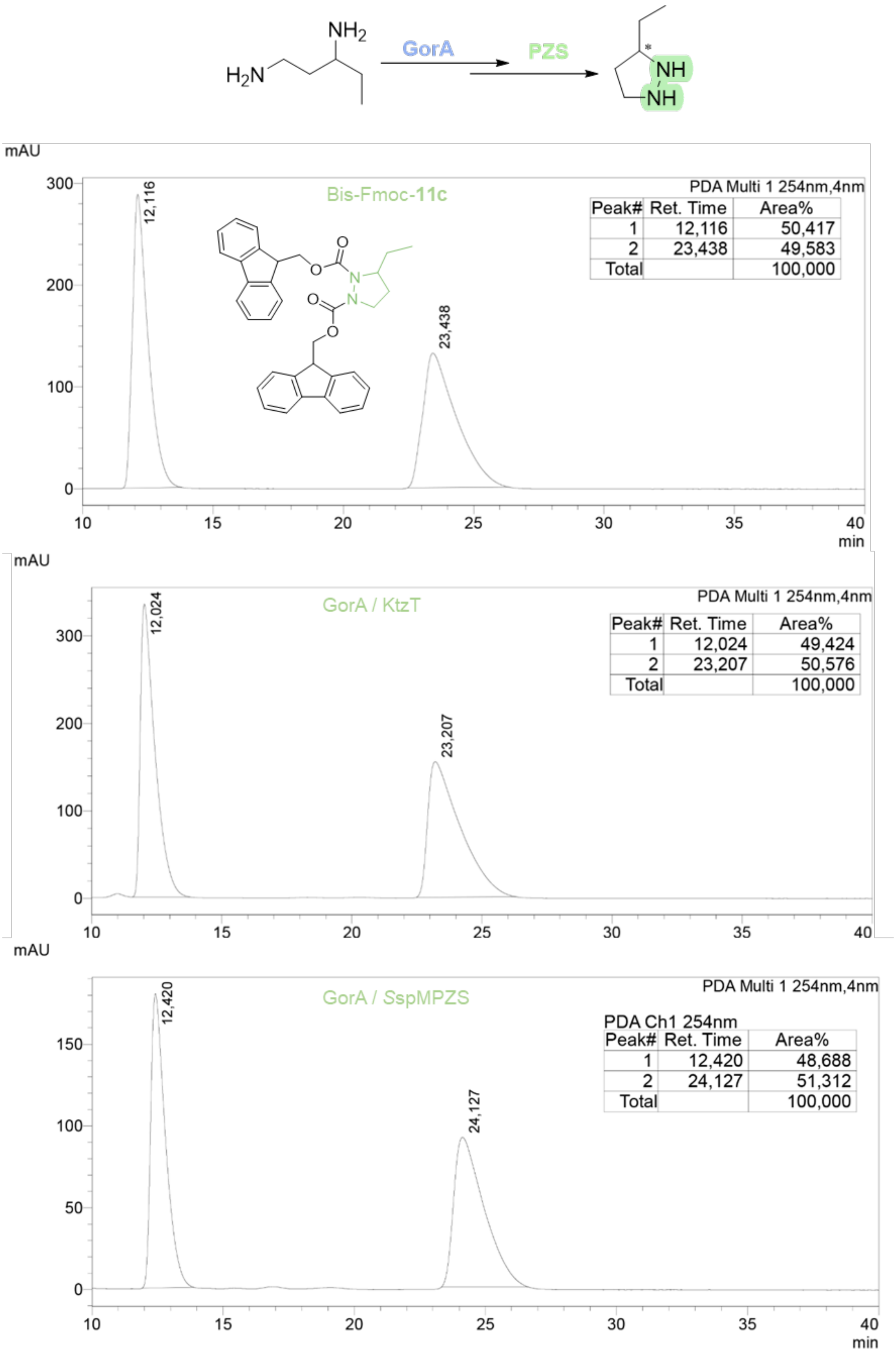

Figure S16. Chiral HPLC chromatogram of racemic reference and enzymatically synthesized Bis-Fmoc-**11c** (from top to bottom) obtained from the coupled reactions of GorA/KtzT, GorA/*S*spMPZS.

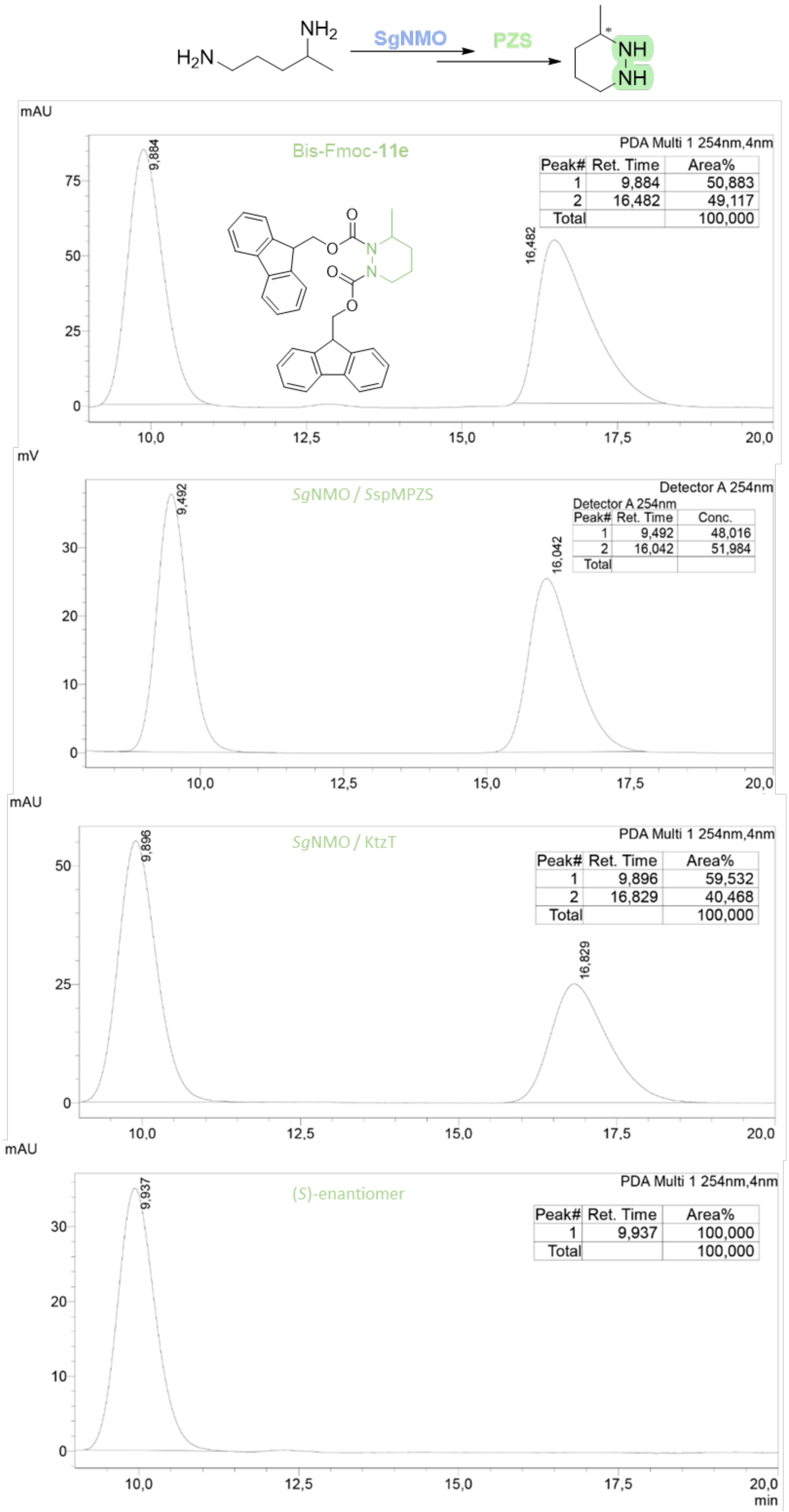

Figure S17. Chiral HPLC chromatogram of racemic reference and enzymatically synthesized Bis-Fmoc-**11e** obtained from the coupled reactions of *Sg*NMO/*S*spMPZS and *Sg*NMO/KtzT, and enzymatically obtained (*S*)-configuration of Bis-Fmoc-**11e** catalyzed by KtzT (from top to bottom).

A

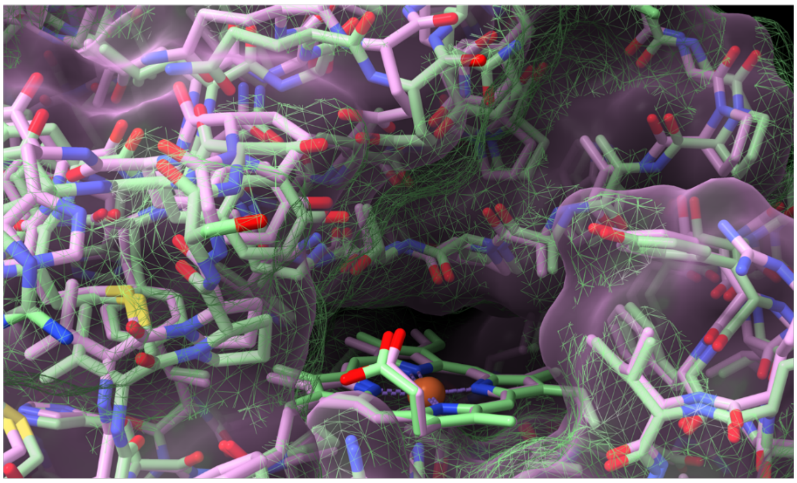

B

```
               1
KtzT           MFVPGPYHAPEDRWLVDLVRGHPLAQLASNGAGGAA--PHITHVPIIVDP
SspMPZS        MFVPSSYREPDSSWMVDLIRANPLALAAANGRPE--DGPFATHLPVIFDP

              51
KtzT           ELDGP-VDRLVGITLWGHMNRANPHWAALGGAANVVATFAGPNAYVSPAV
SspMPZS        DTSGEWTGELPGATLLGHMNRANPHWAALESGSVLLLTFTGPHSYVSPTV

             101
KtzT           YRTAPAAPTWNFTSVQVRGELRKVESADDTLATVRATVAALESRFGAGWD
SspMPZS        YEVTPAAPTWNFTAVHVRGVVEKIDSVQETLGVVESTVRAFEGRFGDGWD

             151
KtzT           MTGSLDYFRRILPGVGAFRLRVAEADGMFKLSQEQQPAIRRRVRHSF--G
SspMPZS        MTASLGYFRKIAPAVGAFRFTVTGAEGMFKLSQEQPGEVRERVRESFGQS

             201
KtzT           GC----EATRAVAGLMDRLPTE
SspMPZS        ACTYKRETAGLMSRLP------
```

Figure S18. (A) Aligned structures of KtzT (pink) and *S*spMPZS (green) models shown with sticks and surface representations. (B) Sequence alignment of KtzT and *S*spMPZS. Residues within 12 Å of the heme-Fe are highlighted green (identical) or red (different, but sidechains not lining the active site)

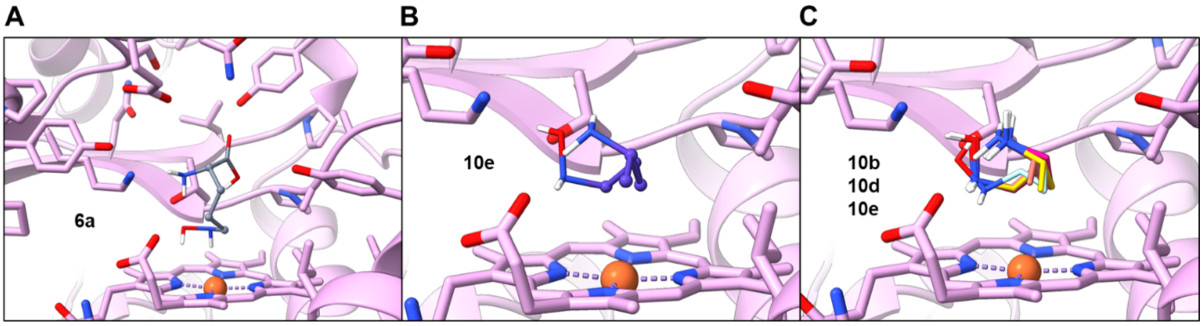

Figure S19. Active site of modeled KtzT with (A) **6a**, (B) **10e** or (C) **10b+10d+10e** docked in the active site illustrating different binding conformations of diamino acids and diamines.

7. Synthetic procedures and NMR spectra of compounds

General procedure A: synthesis of compounds Bis-Fmoc-11a and Bis-Fmoc-11d. Fmoc-Cl (2.2 eq, 569 mg) was added to a solution of **11a** or **11d** (1 mmol) in 9.5 mL THF and 0.5 mL sat. aq. $NaHCO_3$. The reaction mixture was stirred at 50 °C until thin layer chromatography (TLC) indicated that the reaction was completed (around 2 hours). The mixture was acidified with 1N HCl (pH 3 - 4), concentrated under reduced pressure and extracted with diethyl ether (DE, 3 x 20 mL). The combined organic layers were washed with brine, dried with $MgSO_4$ and concentrated. The product was purified using medium-pressure liquid chromatography (MPLC) on silica gel. The eluent gradient used was pentane/DE or heptane/MTBE 0 – 30%.

General procedure B: synthesis of compounds Bis-Fmoc-11c and Bis-Fmoc-11b.

Methyl-(*E*)-pent-2-enoate (for **11c**) or methyl crotonate (for **11b**) (5 mmol) was added dropwise to a solution of hydrazine monohydrate (375 µL, 5 mmol) in ethanol (10 mL). Subsequently, the reaction mixture was refluxed at 80 °C for about 3 hours. Once the reaction had reached completion (the product stains yellow on the TLC with ninhydrin), the solvent was removed under reduced pressure. The crude product was purified *via* MPLC (DCM/methanol, 0 – 50%) to yield a cyclic product as a yellow oil. Then, the obtained product was dissolved in 10 mL of dry tetrahydrofuran (THF), and added dropwise to a solution of 5 mL of 1M $LiAlH_4$ in THF, which had been pre-cooled to -5 °C. The reaction mixture was stirring on ice for 30 minutes, warmed up to room temperature and heated at 60 °C for 4 – 5 hours. After the reaction was completed, it was cooled down to 0 °C, quenched using the Fieser workup method[9] and filtered to remove salts. Subsequently, Fmoc-Cl (4 mmol, 1 g) was added and the reaction was mixed for 2 hours at 50 °C. The mixture was then acidified with 1N HCl (pH 3 - 4), concentrated under reduced pressure and extracted with DE (3 x 20 mL). The combined organic layers were washed with brine, dried with $MgSO_4$ and concentrated. The product was purified using MPLC on silica gel, the eluent gradient used was pentane/DE or heptane/MTBE 0 - 30%.

General procedure C: synthesis of compound Bis-Fmoc-11e. The solution of 1M $LiAlH_4$ (3 mL) in THF was cooled down to -5 °C. Then, a solution of 4,5-dihydro-6-methyl-3(2*H*)-pyridazinone (1.5 mmol, 168 mg) in 3 mL of dry THF was added dropwise. The reaction was mixed on ice for 30 minutes, warmed up to room temperature, and subsequently heated at 60 °C for 4 – 5 hours. Once the reaction was completed, the mixture was cooled down to 0 °C and quenched using the Fieser workup method. The resulting mixture was filtered to remove salts. After that, 2.5 mmol (647 mg) of Fmoc-Cl was added and the reaction was stirred for 2 hours at 50 °C. The mixture was acidified with 1N HCl (pH 3 - 4), concentrated under reduced pressure and extracted with DE (3 x 20 mL). The combined organic layers were washed with brine, dried with $MgSO_4$ and concentrated. The product was purified using MPLC on silica gel, the eluent gradient used was pentane/DE or heptane/MTBE 0 - 30%.

General Procedure D: **biocatalytic microscale reaction.** To a solution of NH_4HCO_3 (pH 8.5, 20 mM) in a 250-mL Erlenmeyer flask, 1 mM $NADP^+$, 10 mM glucose, 0.05 mM FAD, 10 U/mL GDH, 1 mg/mL catalase, 0.03 mmol diamine was successively added to a total volume of 6 x 15 mL. The reaction was started by the addition of 50 µM NMO and 2 µM KtzT/*S*spMPZS/*A*spPZS/*S*spPZS. After derivatization with Fmoc-Cl, the mixture was extracted with EtOAc, dried and purified using preparative HPLC (isocratic mixture of 70% acetonitrile with 0.1% formic acid).

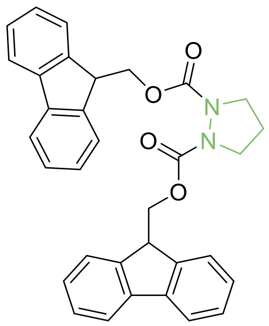

(**Bis-Fmoc-11a**) was synthesized by following general procedure **A** to afford **Bis-Fmoc-11a** (299 mg, 58%) as a white solid. **^{1}H NMR** (600 MHz, DMSO-d_6) δ 7.88 (d, J = 7.6 Hz, 4H), 7.63 (dd, J = 23.2, 7.6 Hz, 4H), 7.40 (q, J = 7.3 Hz, 4H), 7.29 (q, J = 7.3 Hz, 4H), 4.39 (d, J = 6.8 Hz, 4H), 4.23 (t, J = 6.8 Hz, 2H), 3.66 (s, 2H), 3.08 (d, J = 9.0 Hz, 2H), 1.83 (t, J = 7.0 Hz, 2H) ppm. **^{13}C NMR** (151 MHz, DMSO-d_6) δ 143.9, 143.5, 143.5, 140.8, 140.7, 139.4, 128.9, 127.7, 127.6, 127.3, 127.1, 125.1, 121.4, 120.1, 120.1, 120.0, 67.3, 46.5, 46.5, 25.1 ppm. **HRMS** (ESI$^+$), m/z calculated for $C_{33}H_{29}N_2O_4$, $[M+H]^+$ 517.2122, found 517.2116.

***enz*-Bis((9H-fluoren-9-yl)methyl) pyrazolidine-1,2-dicarboxylate (Bis-Fmoc-11a)** was synthesized by following general procedure **D** to afford **Bis-Fmoc-11a** (4 mg, 45%) as a white solid.**^{1}H NMR** (600 MHz, DMSO-d_6) δ 7.87 (d, J = 7.8 Hz, 4H), 7.63 (dd, J = 22.3, 7.6 Hz, 4H), 7.44 – 7.37 (m, 4H), 7.30-7.27 (m, 4H) 4.39 (d, J = 6.6 Hz, 4H), 4.22 (t, J = 6.6 Hz, 2H), 3.65 (s, 2H), 3.07 (s, 2H), 1.82 (s, 2H) ppm. **HRMS** (ESI$^+$), m/z calculated for $C_{33}H_{29}N_2O_4$, $[M+H]^+$ 517.2122, found 517.2122.

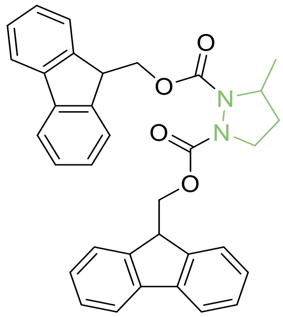

Bis((9H-fluoren-9-yl)methyl) 3-methylpyrazolidine-1,2-dicarboxylate (Bis-Fmoc-11b) was synthesized by following general procedure **B** to afford **Bis-Fmoc-11b** (239 mg, 9% overall) as a white solid. **^{1}H NMR** (600 MHz, DMSO-d_6) δ 7.90 – 7.83 (m, 4H), 7.68 – 7.56 (m, 4H), 7.43 – 7.34 (m, 4H), 7.32 – 7.20 (m, 4H), 4.54 – 4.27 (m, 4H), 4.21 (d, J = 7.2 Hz, 2H), 3.98 (s, 1H), 3.67 (s, 1H), 3.03 (d, J = 10.0 Hz, 1H), 2.04 (s, 1H), 1.41 (s, 1H), 0.93 (d, J = 6.5 Hz, 3H) ppm. **^{13}C NMR** (151 MHz, DMSO-d_6) δ 156.8, 156.0, 143.6, 143.5, 143.5, 142.6, 140.8, 139.4, 137.4, 128.9, 127.7, 127.7, 127.6, 127.6, 127.3, 127.1, 127.0, 125.1, 125.0, 125.0, 121.4, 120.1, 120.1, 120.0, 109.7, 67.1, 67.1, 64.9, 54.9, 46.6, 46.5, 32.9, 20.1 ppm. **HRMS** (ESI$^+$), m/z calculated for $C_{34}H_{31}N_2O_4$, $[M+H]^+$ 531.2278, found 531.2292.

***enz*-Bis((9H-fluoren-9-yl)methyl) 3 methylpyrazolidine-1,2-dicarboxylate (Bis-Fmoc-11b)** was synthesized by following general procedure **D** to afford **Bis-Fmoc-11b** (2.1 mg, 40%) as a white solid. **^{1}H NMR** (600 MHz, DMSO-d_6) δ 7.91 – 7.84 (m, 4H), 7.70 – 7.58 (m, 4H), 7.45 – 7.20 (m, 8H), 4.59 – 4.27 (m, 4H), 4.22 (t, J = 6.8 Hz, 2H), 4.00 (s, 1H), 3.64 (d, J = 45.3 Hz, 1H), 3.12 – 2.98 (m, 1H), 2.06 (s, 1H), 1.43 (s, 1H), 0.94 (d, J = 6.4 Hz, 3H) ppm. **HRMS** (ESI$^+$), m/z calculated for $C_{34}H_{31}N_2O_4$, $[M+H]^+$ 531.2278, found 531.2287.

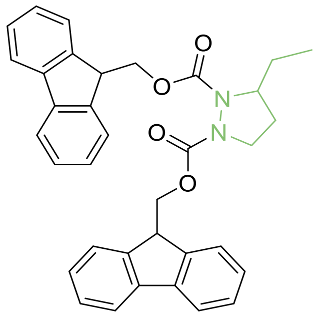

Bis((9H-fluoren-9-yl)methyl) 3-ethylpyrazolidine-1,2-dicarboxylate (Bis-Fmoc-11c) was synthesized by following general procedure **B** to afford **Bis-Fmoc-11c** (218 mg, 13% overall) as a white solid. **^{1}H NMR** (600 MHz, DMSO-d_6) δ 7.89 – 7.83 (m, 4H), 7.64 (t, J = 8.1 Hz, 2H), 7.59 (dd, J = 12.4, 7.5 Hz, 2H), 7.42 – 7.34 (m, 4H), 7.28 (q, J = 6.8 Hz, 3H), 7.22 (t, J = 7.4 Hz, 1H), 4.61 – 4.30 (m, 4H), 4.26 – 4.13 (m, 2H), 3.67 (br s, 2H), 2.99 (d, J = 9.7 Hz, 1H), 1.93 (br s, 1H), 1.42 (br s, 1H), 1.14 (s, 1H), 1.07 (s, 1H), 0.66 (s, 3H) ppm. **^{13}C NMR** (151 MHz, DMSO-d_6) δ 156.5, 143.7, 143.6, 143.5, 143.4, 140.9, 140.8, 140.8, 127.7, 127.7, 127.6, 127.1, 127.0, 127.0, 125.0, 125.0, 120.1, 120.0, 67.1, 66.9, 60.5, 46.7, 46.6, 46.3, 30.6, 26.8, 10.3 ppm. **HRMS** (ESI$^+$), m/z calculated for $C_{35}H_{33}N_2O_4$, $[M+H]^+$ 545.2435, found 545.2437.

***enz*-Bis((9H-fluoren-9-yl)methyl) pyrazolidine-1,2-dicarboxylate (Bis-Fmoc-11c**) was synthesized by following general procedure **D** to afford **Bis-Fmoc-11c** (2.3 mg, 33%) as a white solid. **^{1}H NMR** (600 MHz, DMSO-d_6) δ 7.92 – 7.83 (m, 4H), 7.69 – 7.56 (m, 4H), 7.44 – 7.20 (m, 8H), 4.42 (d, J = 9.4 Hz, 4H), 4.27 – 4.14 (m, 2H), 3.69 (s, 2H),

3.06 – 2.93 (m, 1H), 1.95 (s, 1H), 1.43 (s, 1H), 1.15 (s, 1H), 1.07 (s, 1H), 0.67 (s, 3H) ppm. **HRMS** (ESI$^+$), m/z calculated for $C_{35}H_{33}N_2O_4$, [M+H]$^+$ 545.2435, found 545.2431.

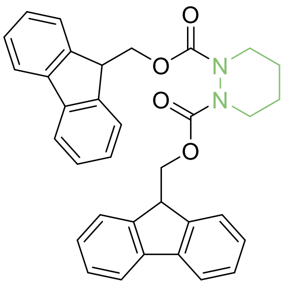

Bis((9H-fluoren-9-yl)methyl) tetrahydropyridazine-1,2-dicarboxylate (Bis-Fmoc-11d) was synthesized by following general procedure **A** to afford **Bis-Fmoc-11d** (334 mg, 63%) as a white solid. ^{1}**H NMR** (600 MHz, DMSO) δ 7.95 – 7.78 (m, 4H), 7.71 – 7.15 (m, 12H), 4.77 – 4.05 (m, 6H), 3.93 (d, J = 13.2 Hz, 1H), 3.69 – 3.47 (m, 1H), 2.86 – 2.56 (m, 2H), 1.62 – 1.32 (m, 3H), 1.13 (s br, 1H) ppm. ^{13}**C NMR** (151 MHz, DMSO-*d*$_6$) δ 154.5, 143.4, 140.9, 127.7, 127.7, 127.0, 125.1, 124.9, 120.2, 67.3, 46.7, 46.5, 44.7, 22.8 ppm. **HRMS** (ESI$^+$), m/z calculated for $C_{34}H_{31}N_2O_4$, [M+H]$^+$ 531.2278, found 531.2292.

***enz*-Bis((9H-fluoren-9-yl)methyl) tetrahydropyridazine-1,2-dicarboxylate (Bis-Fmoc-11d)** was synthesized by following general procedure **D** to afford **Bis-Fmoc-11d** (2.6 mg, 13%) as a white solid. **HRMS** (ESI$^+$), m/z calculated for $C_{34}H_{31}N_2O_4$, [M+H]$^+$ 531.2278, found 531.2294. Note: The presence of impurities in the sample interferes with the assignment of the NMR spectrum.

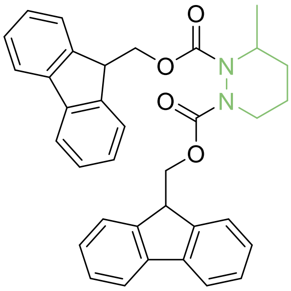

Bis((9H-fluoren-9-yl)methyl) 3-methyltetrahydropyridazine-1,2-dicarboxylate (bis-Fmoc-11e) was synthesized by following general procedure **C** to afford **Bis-Fmoc-11e** (220 mg, 27% overall) as a white solid. ^{1}**H NMR** (600 MHz, DMSO-*d*$_6$) δ 7.97 – 7.49 (m, 7H), 7.46 – 7.05 (m, 9H), 4.75 – 4.52 (m, 1H), 4.46 – 4.20 (m, 4H), 4.16 – 3.85 (m, 3H), 3.50 (d, J = 5.7 Hz, 1H), 2.79 – 2.55 (m, 1H), 1.74 – 1.37 (m, 2H), 1.32 (d, J = 12.3 Hz, 1H), 0.99 – 0.74 (m, 3H) ppm. ^{13}**C NMR** (151 MHz, DMSO-*d*$_6$) δ 154.3, 143.5, 140.8, 127.7, 127.7, 127.5, 127.5, 127.2, 126.9, 126.8, 125.1, 124.9, 124.8, 120.1, 120.0, 67.1, 46.3, 27.3, 18.0 ppm. **HRMS** (ESI$^+$), m/z calculated for $C_{35}H_{33}N_2O_4$, [M+H]$^+$ 545.2435, found 545.2436.

***enz*-Bis((9H-fluoren-9-yl)methyl) 3-ethylpyrazolidine-1,2-dicarboxylate (Bis-Fmoc-11e):** was synthesized by following general procedure **D** to afford **Bis-Fmoc-11e** (1.1 mg, 9%) as a white solid. ^{1}**H NMR** (600 MHz, DMSO-*d*$_6$) δ 7.98 – 7.49 (m, 7H), 7.46 – 7.05 (m, 9H), 4.76 – 4.53 (m, 1H), 4.45 – 4.21 (m, 4H), 4.17 – 3.84 (m, 3H), 3.55 – 3.45 (m, 1H), 2.76 – 2.56 (m, 1H), 1.74 – 1.37 (m, 2H), 1.32 (d, J = 12.7 Hz, 1H), 0.95 – 0.75 (m, 3H) ppm. **HRMS** (ESI$^+$), m/z calculated for $C_{35}H_{33}N_2O_4$, [M+H]$^+$ 545.2435, found 545.2437.

^{1}H and ^{13}C NMR spectra of chemically synthesized compound **Bis-Fmoc-11a**

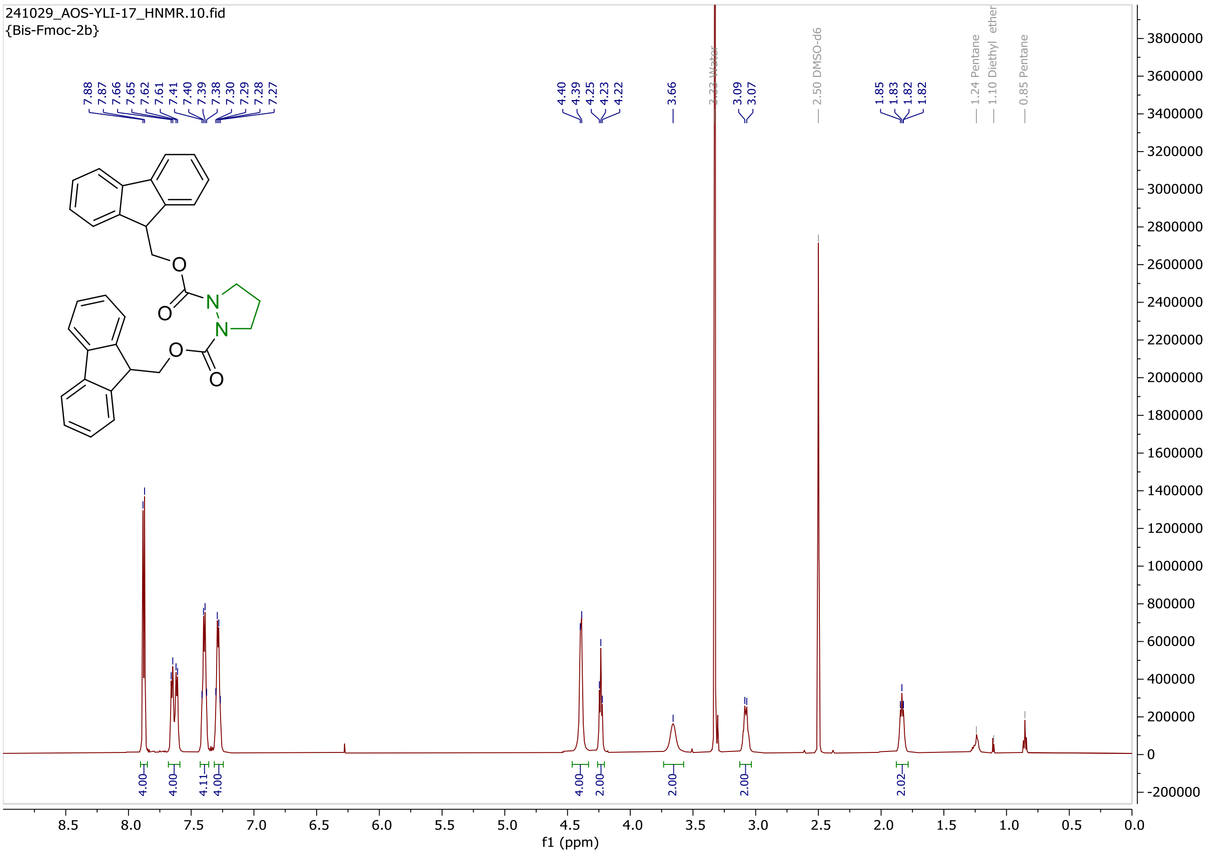

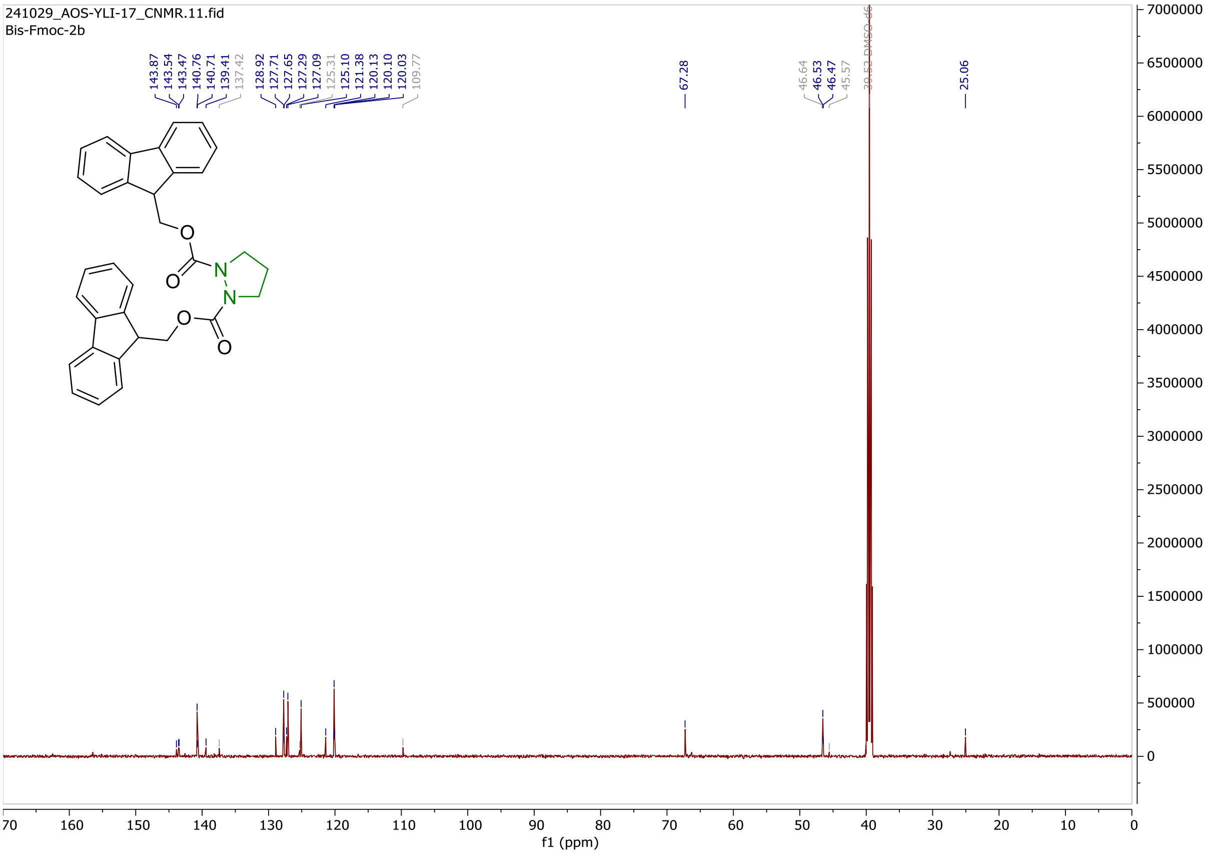

Comparison of ^{1}H spectra of ***enz*-Bis-Fmoc-11a** with the reference

Enzymatic reaction

Chemical reference

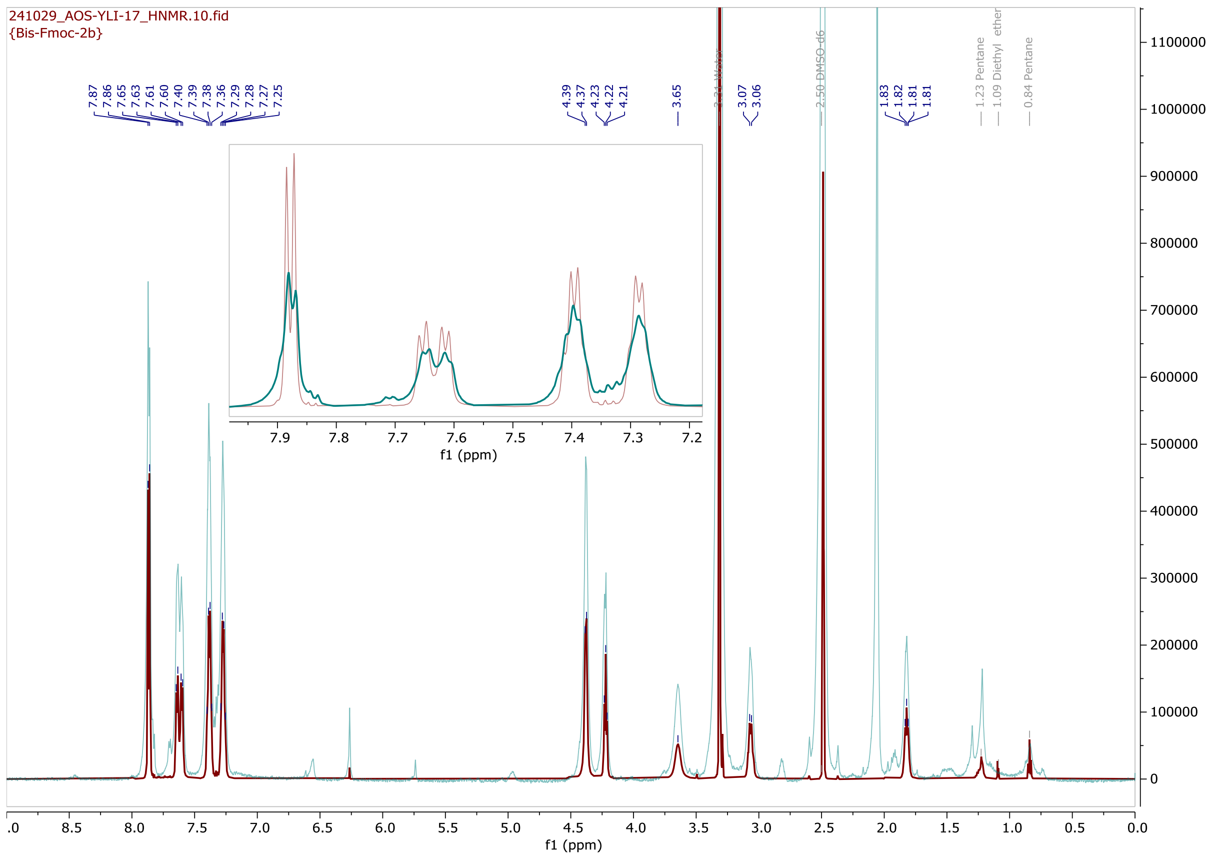

^{1}H and ^{13}C NMR spectra of chemically synthesized compound **Bis-Fmoc-11b**

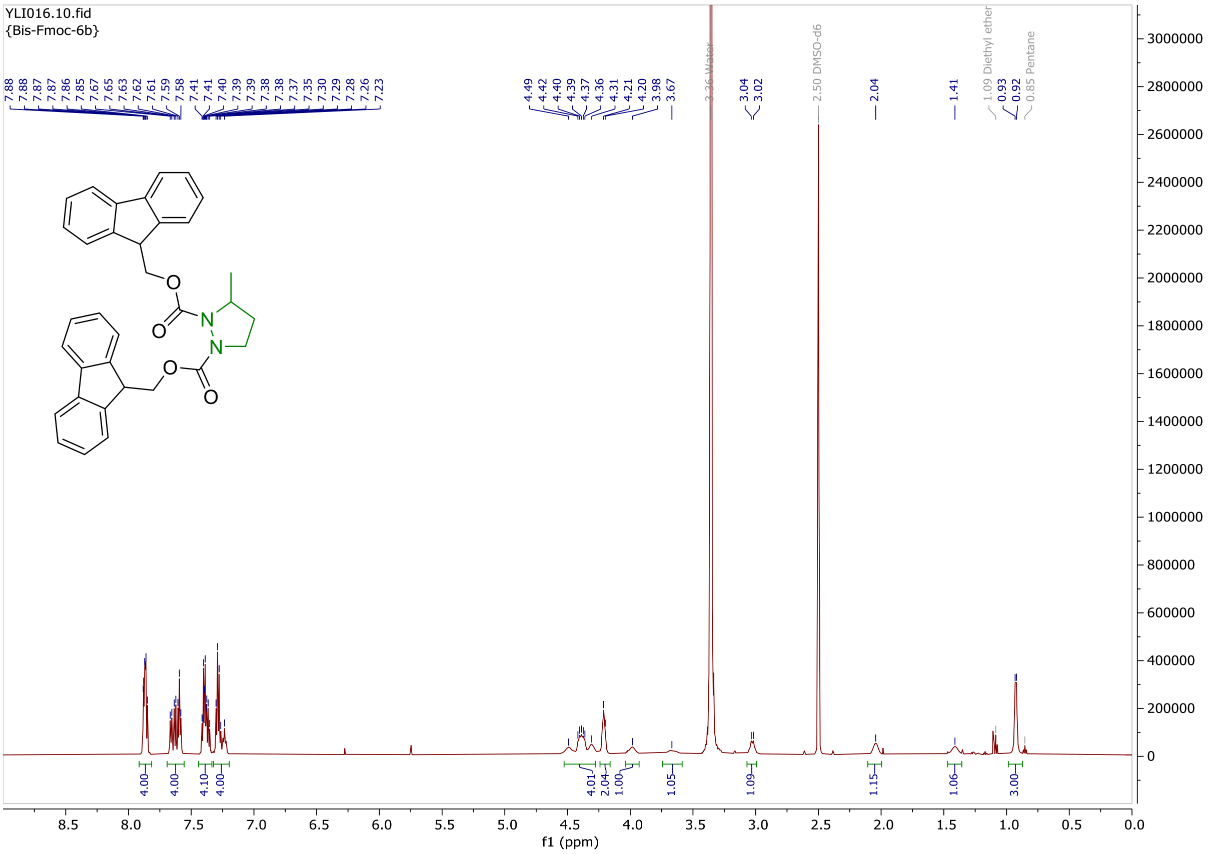

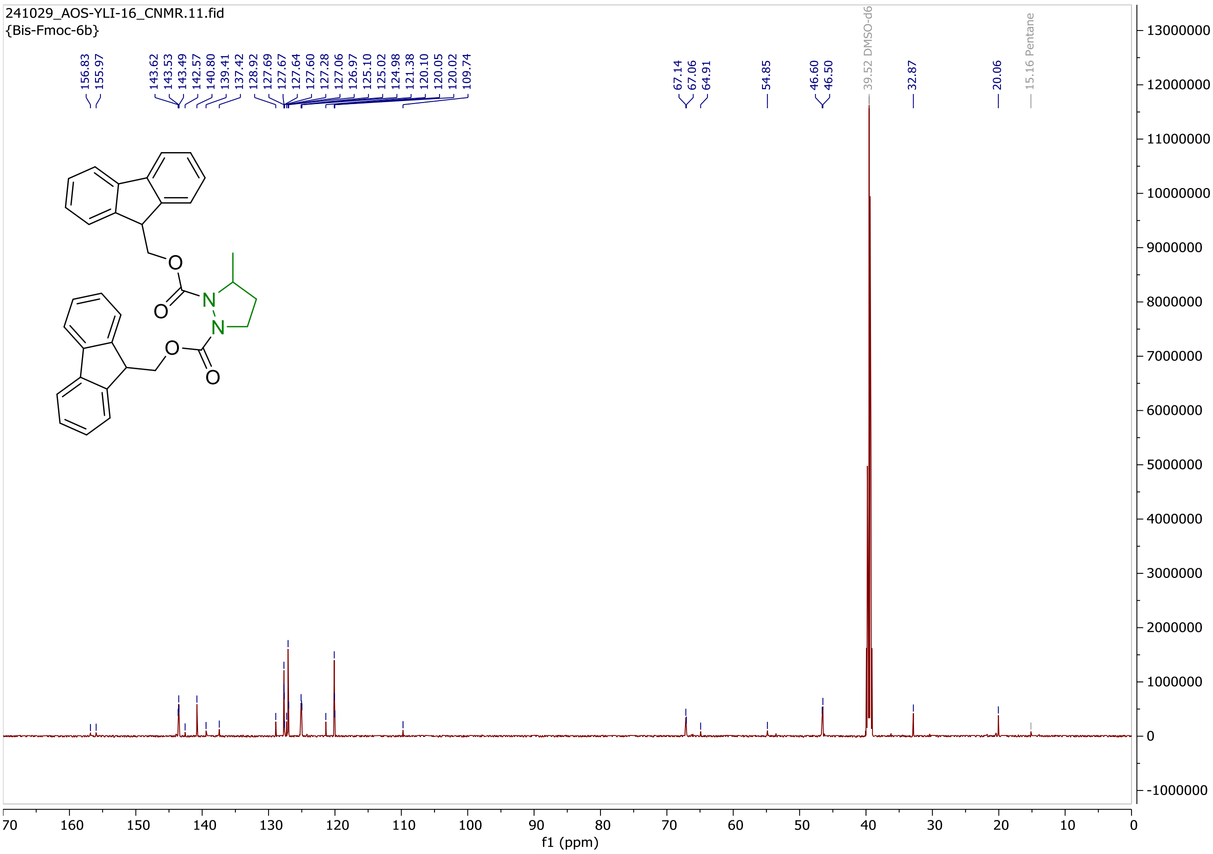

Comparison of ^{1}H spectra of ***enz*-Bis-Fmoc-11b** with the reference

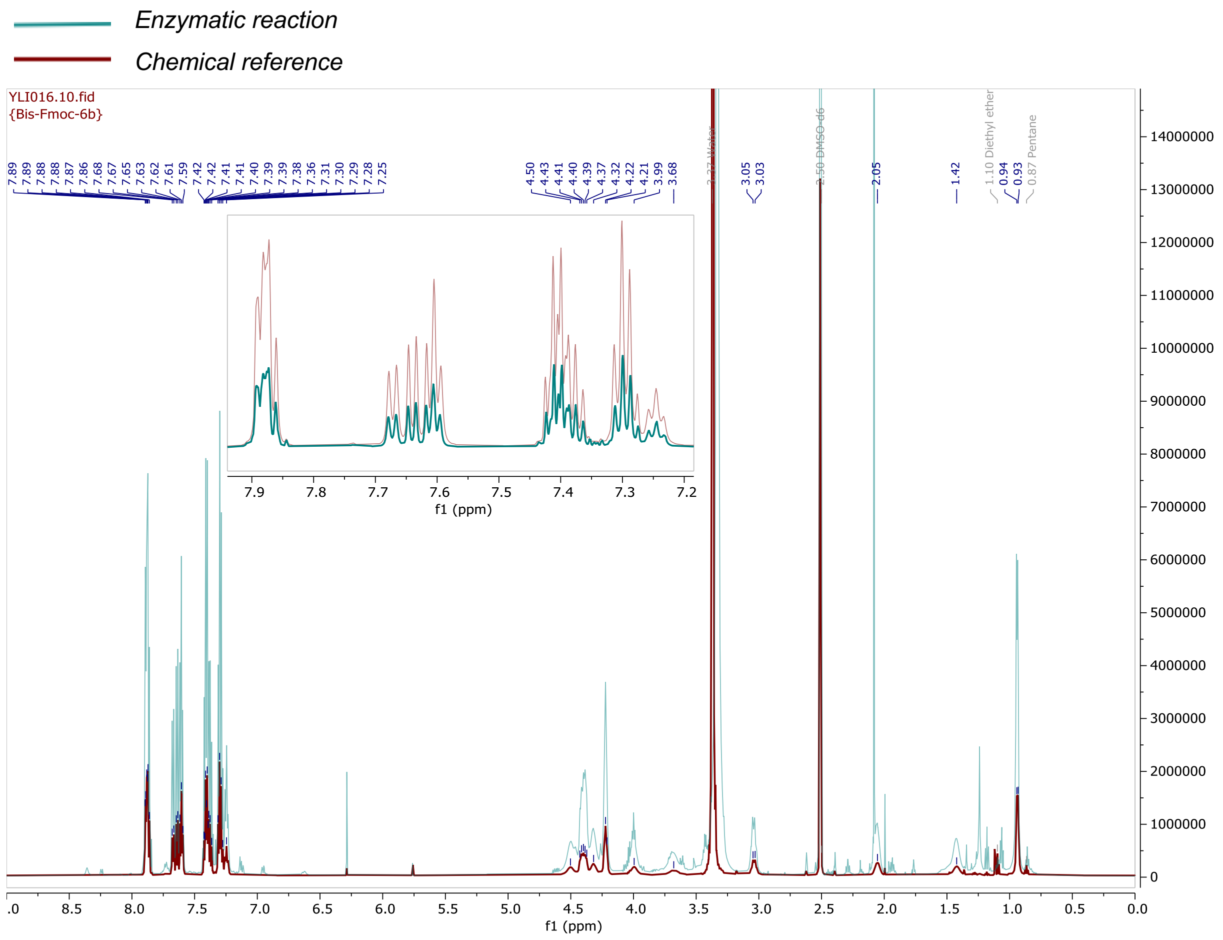

^{1}H and ^{13}C NMR spectra of chemically synthesized compound **Bis-Fmoc-11c**

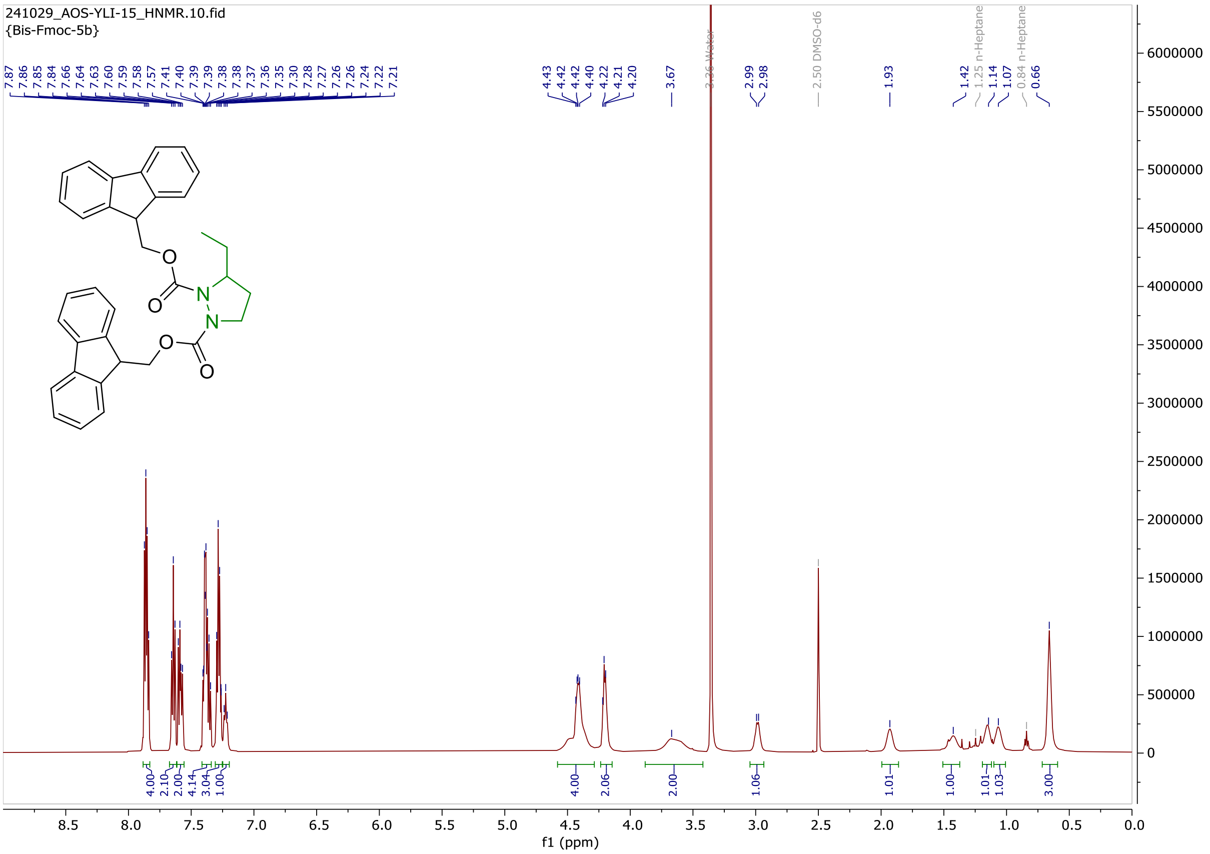

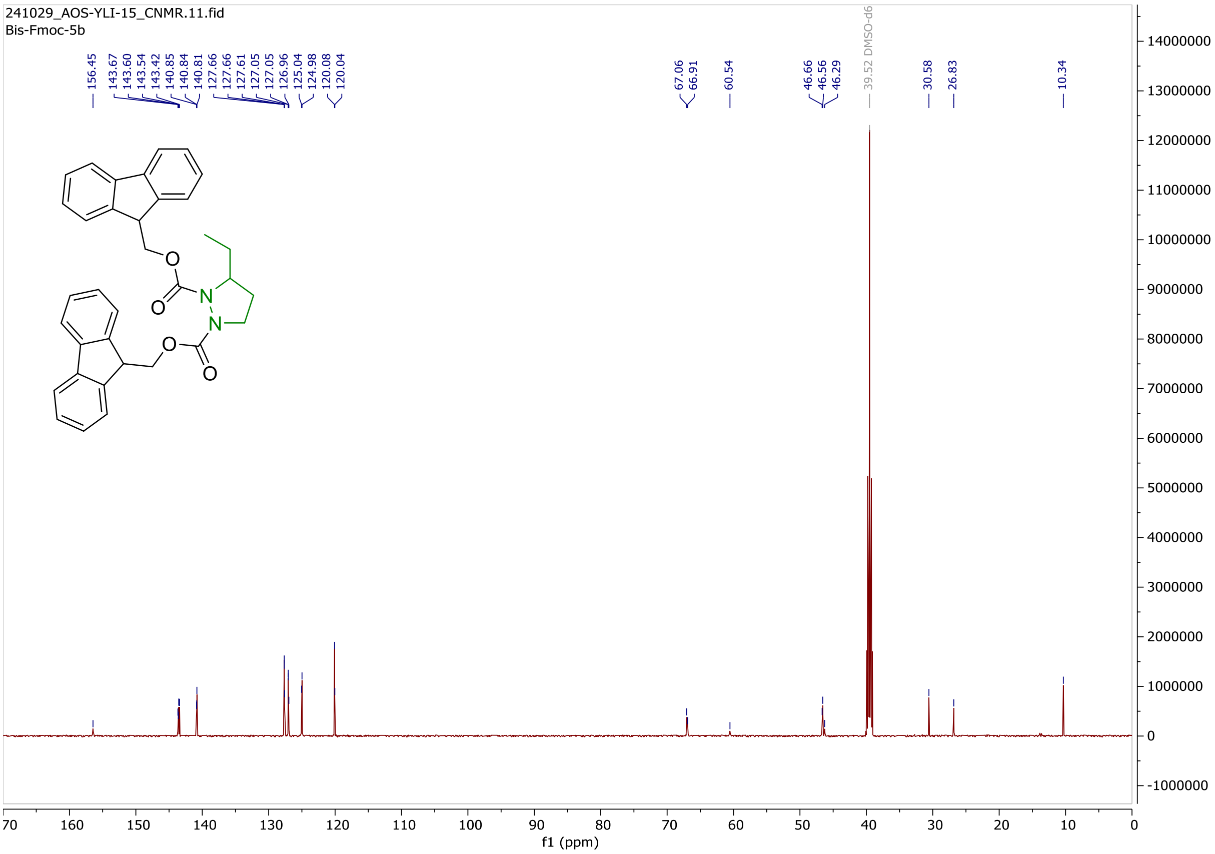

Comparison of ^{1}H spectra of ***enz*-Bis-Fmoc-11c** with the reference

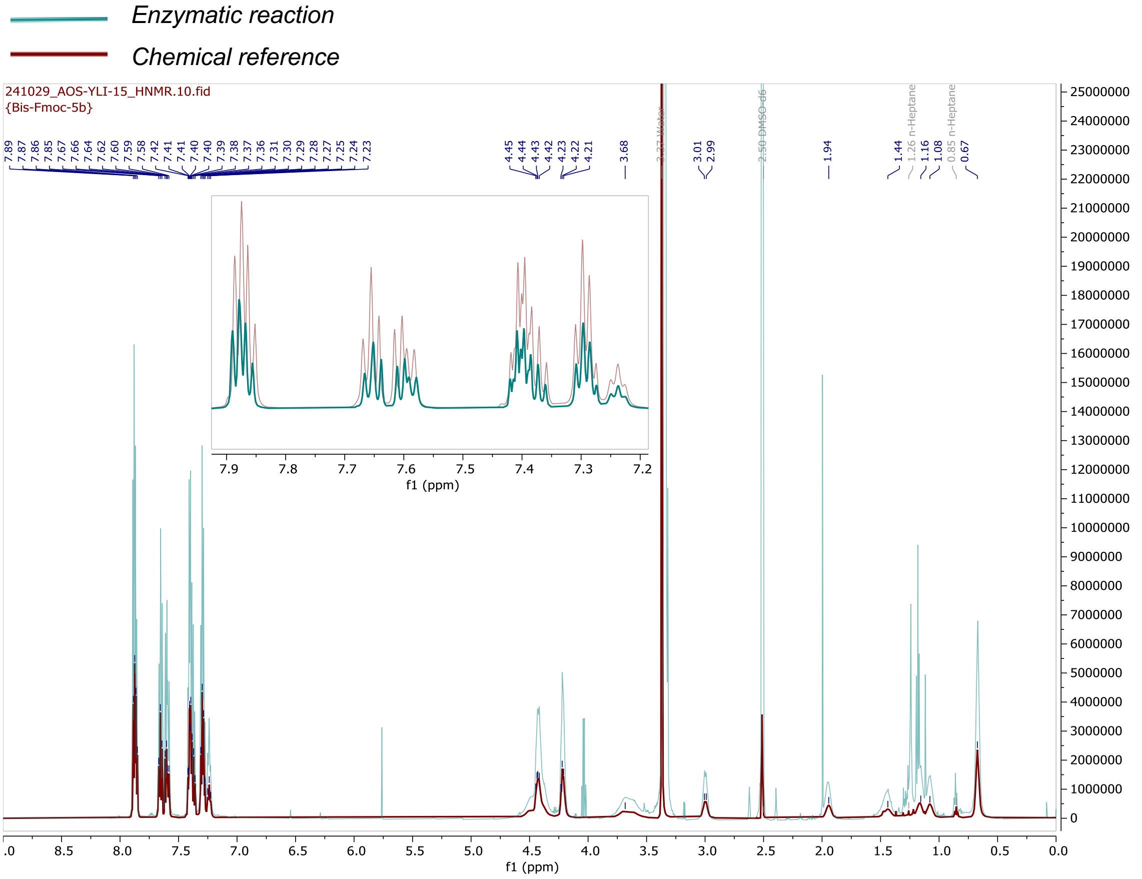

HMBC spectrum of chemically synthesized compound **Bis-Fmoc-11c**

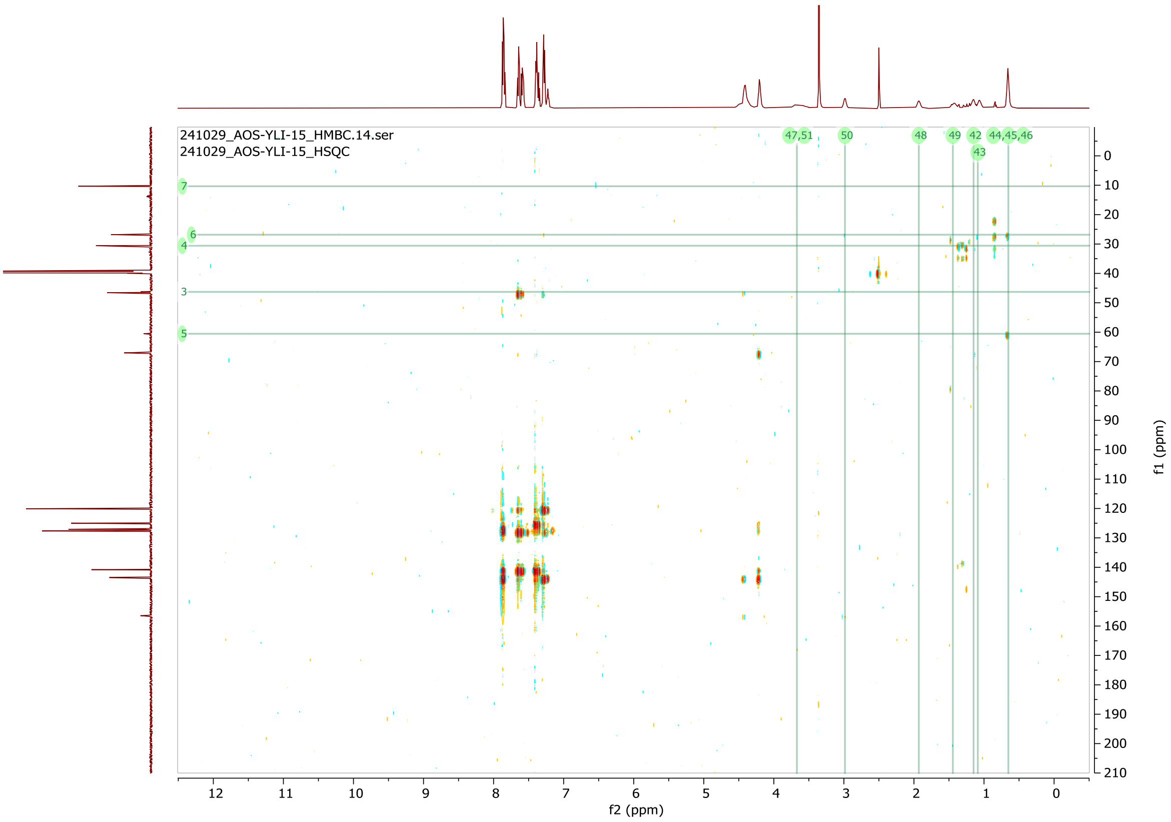

HSQC spectrum of chemically synthesized compound **Bis-Fmoc-11c**

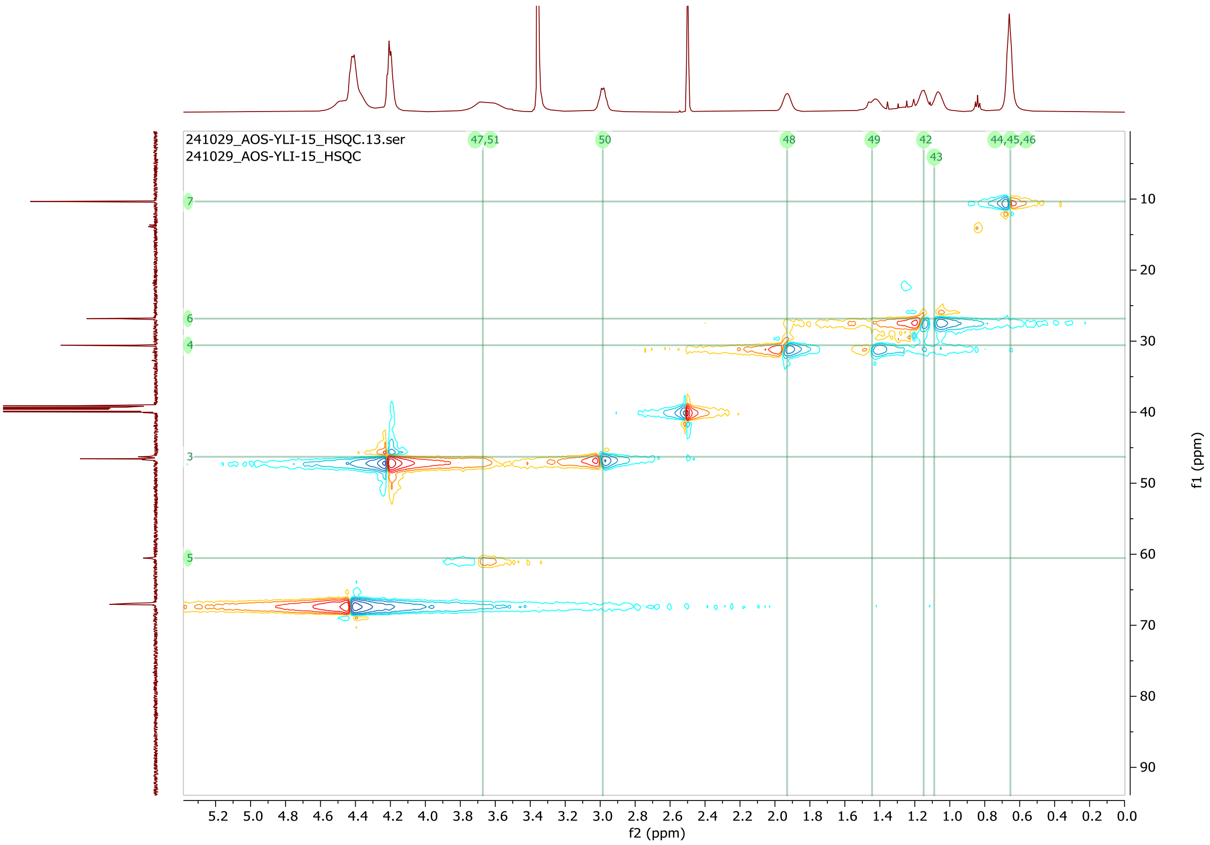

COSY spectrum of chemically synthesized compound **Bis-Fmoc-11c**

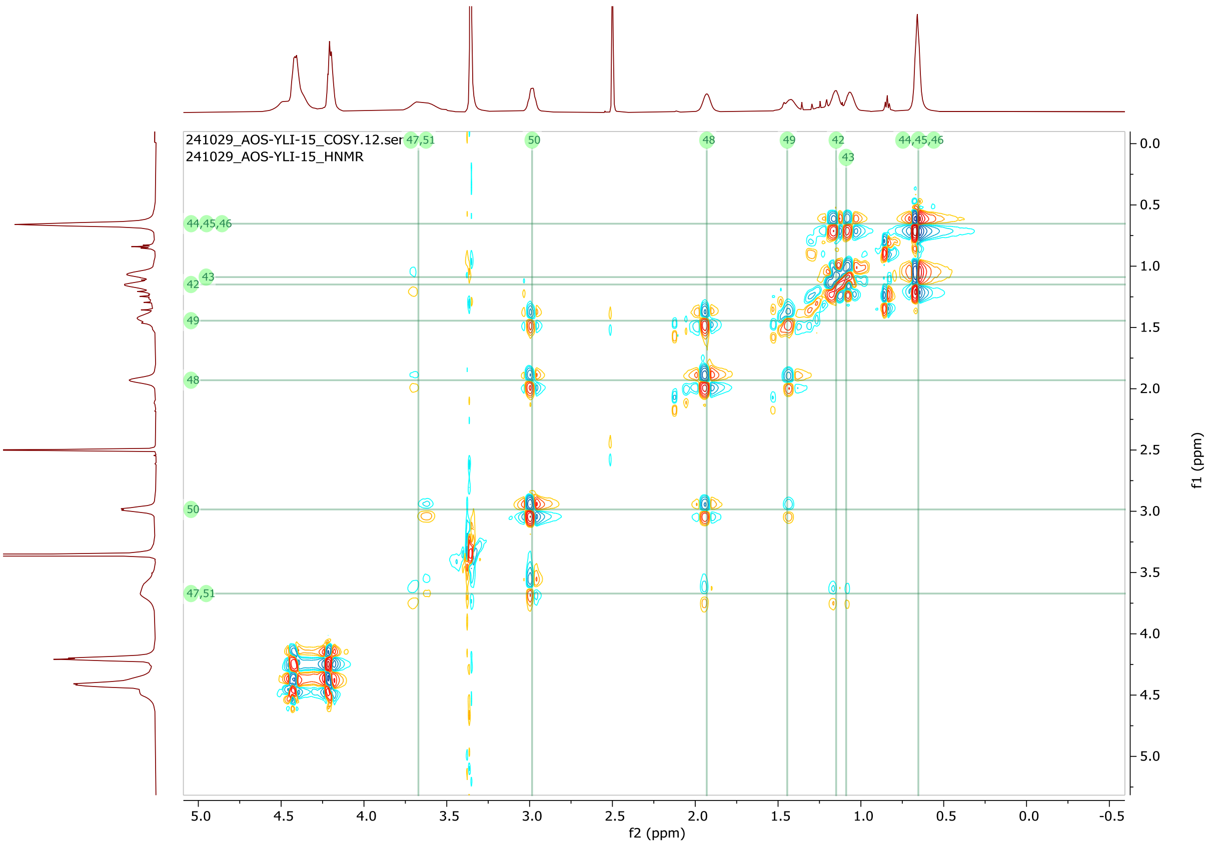

^{1}H and ^{13}C NMR spectra of chemically synthesized compound **Bis-Fmoc-11d**

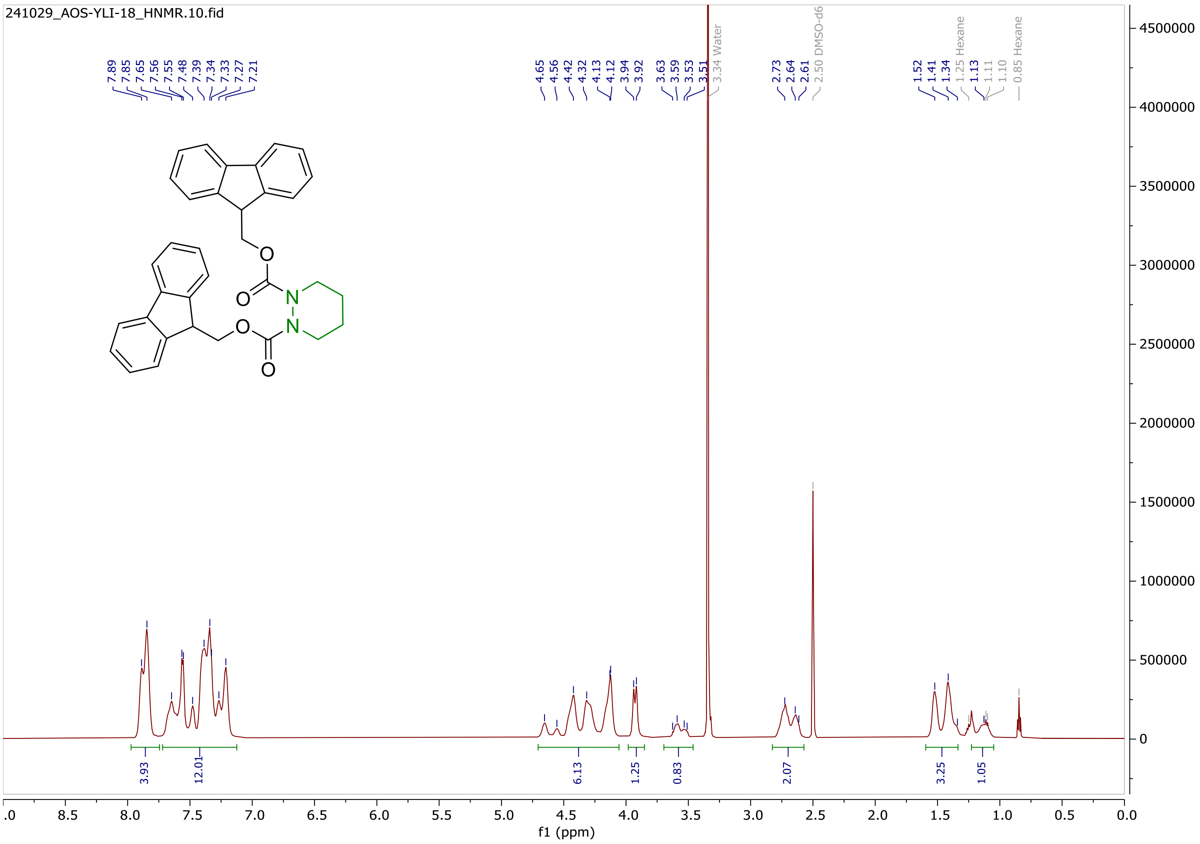

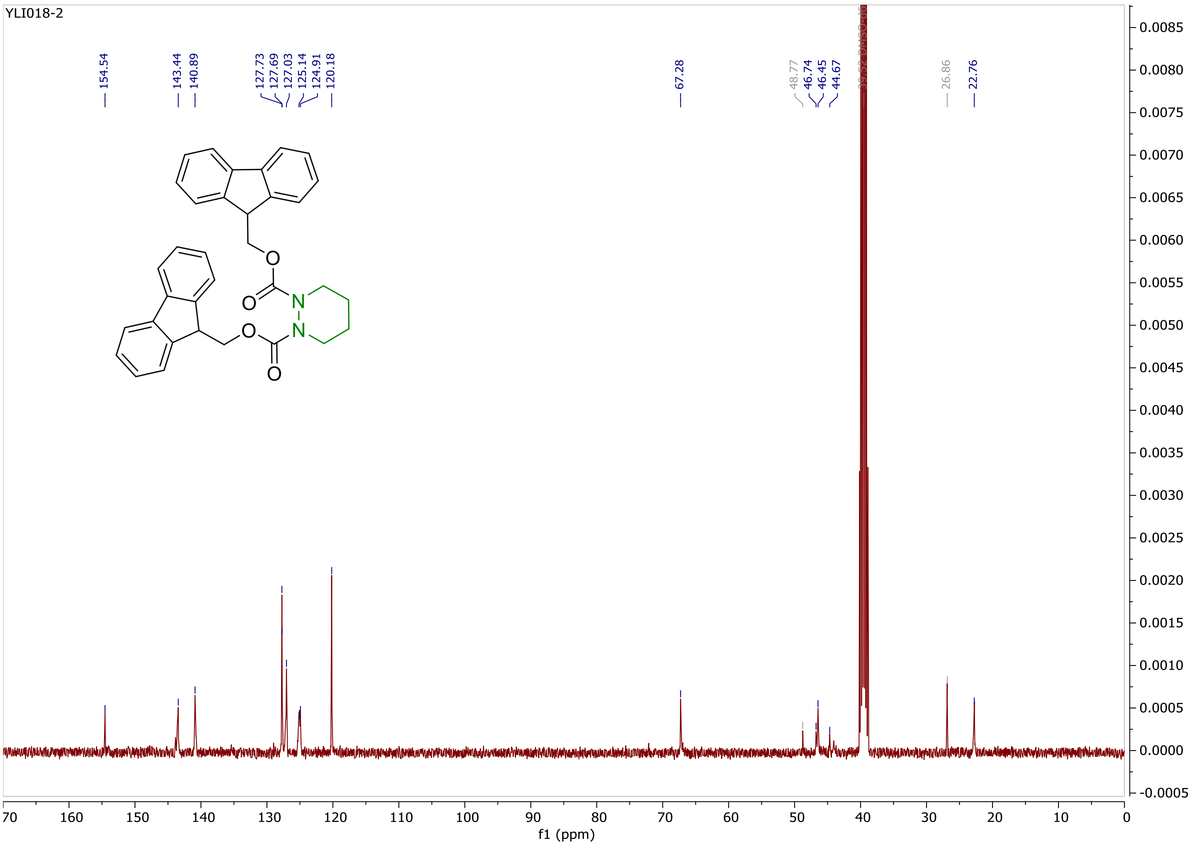

HSQC spectrum of chemically synthesized compound **Bis-Fmoc-11d**

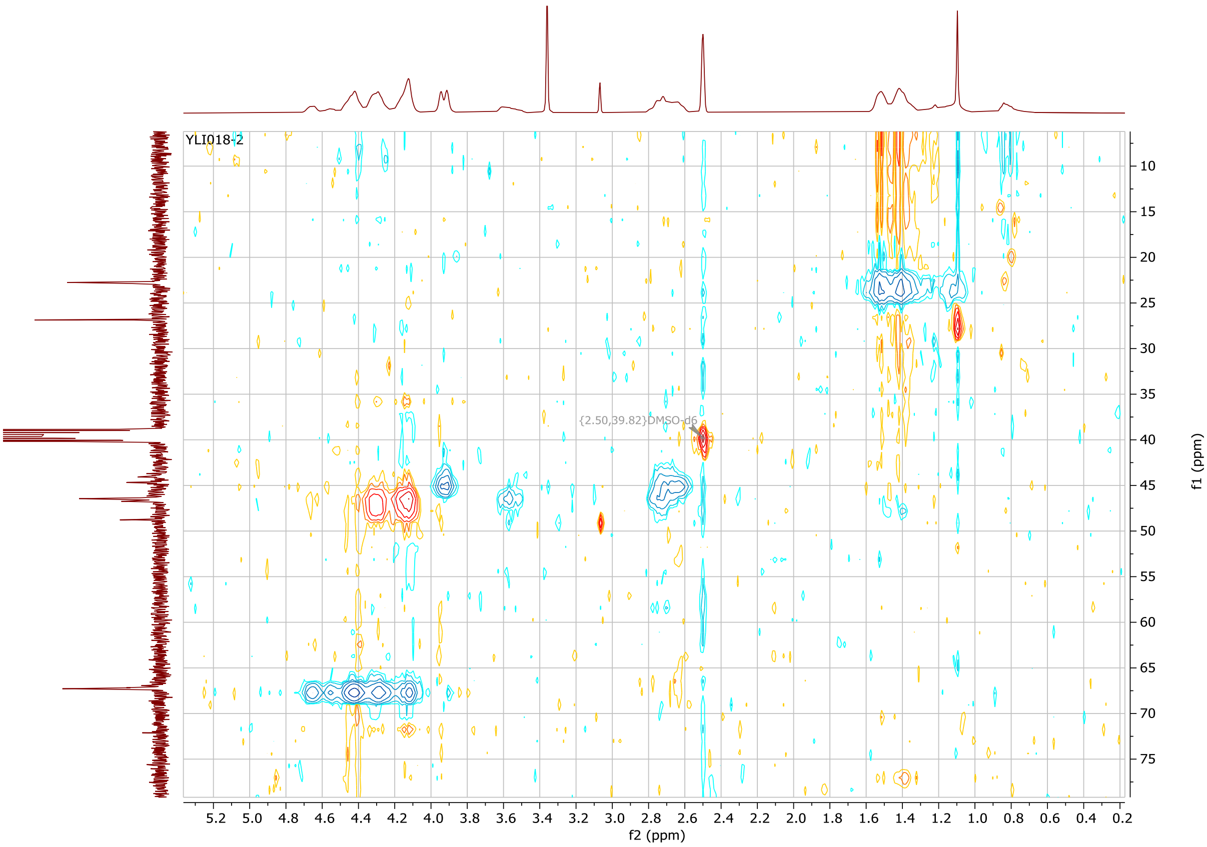

Comparison of ^{1}H spectra of ***enz*-Bis-Fmoc-11d** with the reference

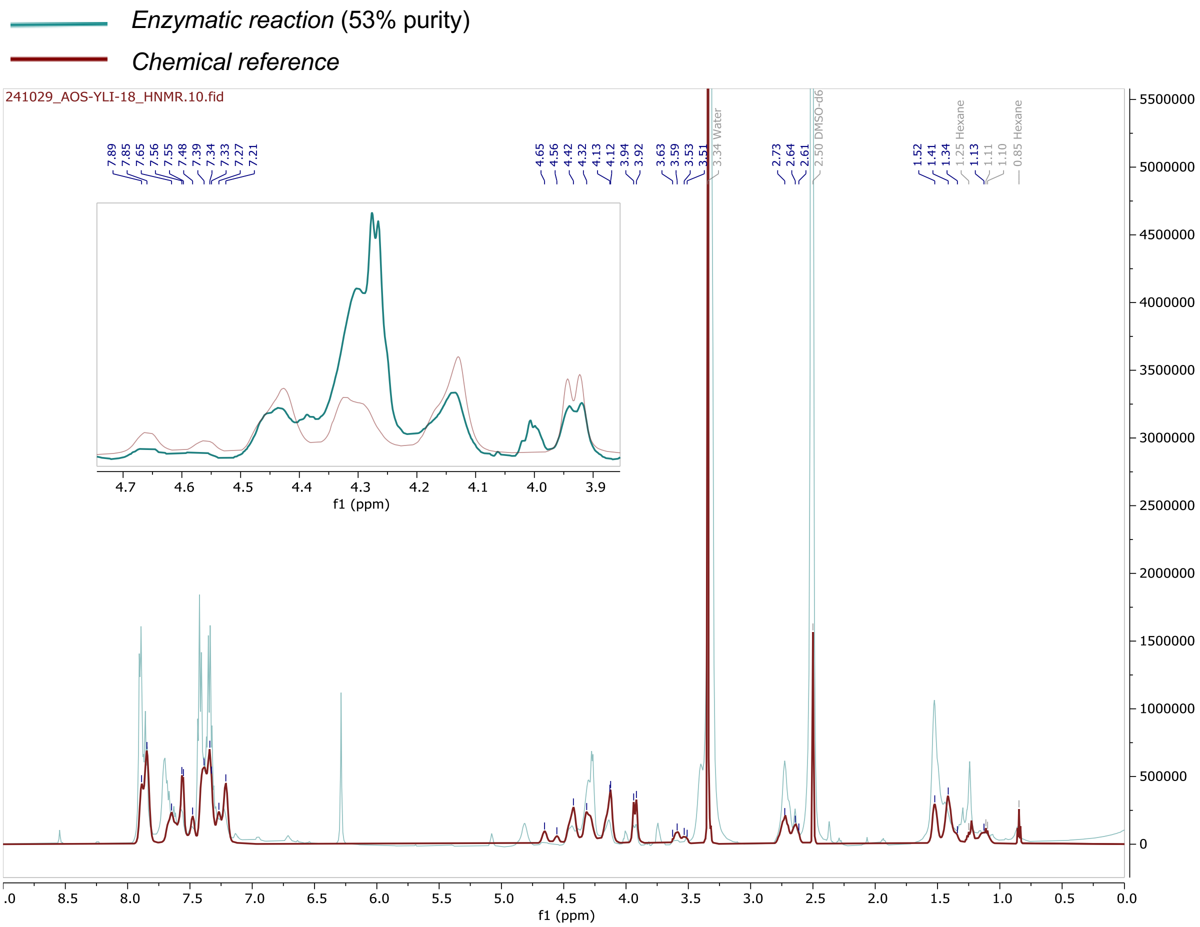

^{1}H and ^{13}C NMR spectra of chemically synthesized compound **Bis-Fmoc-11e**

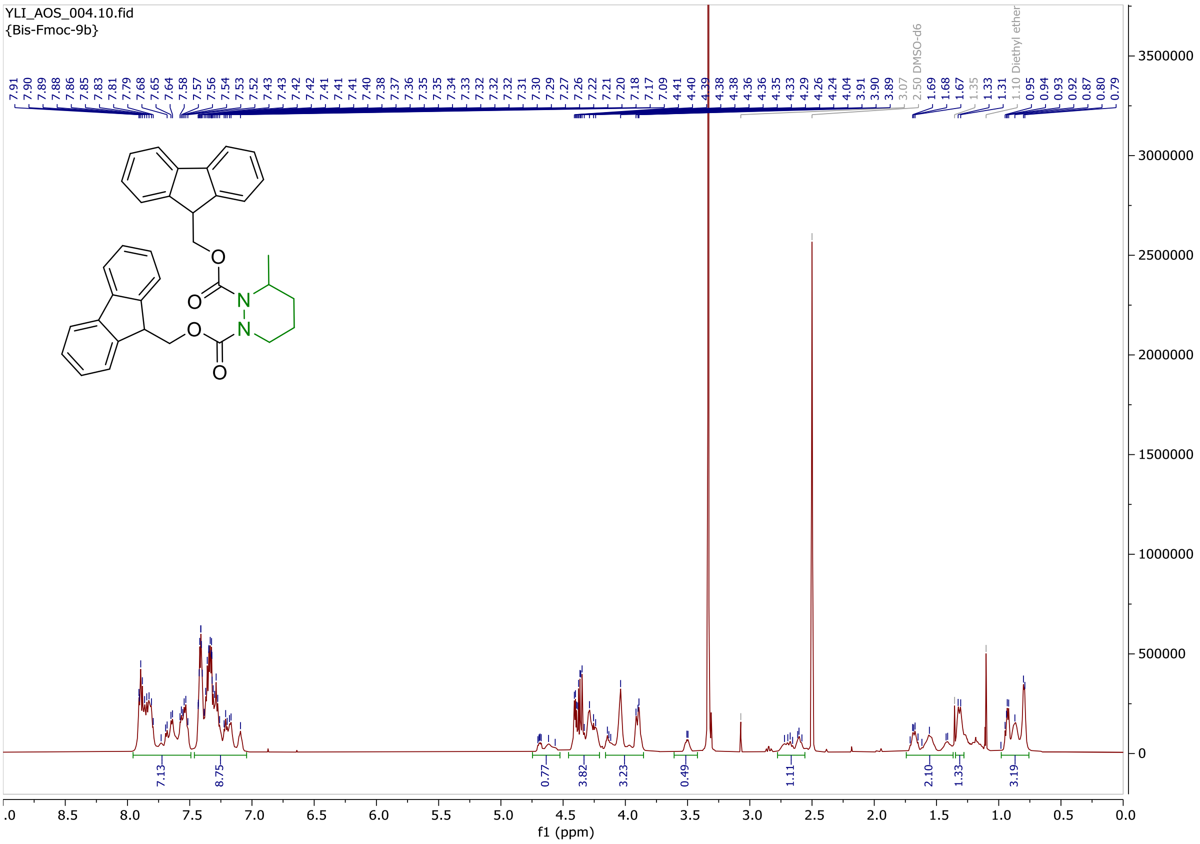

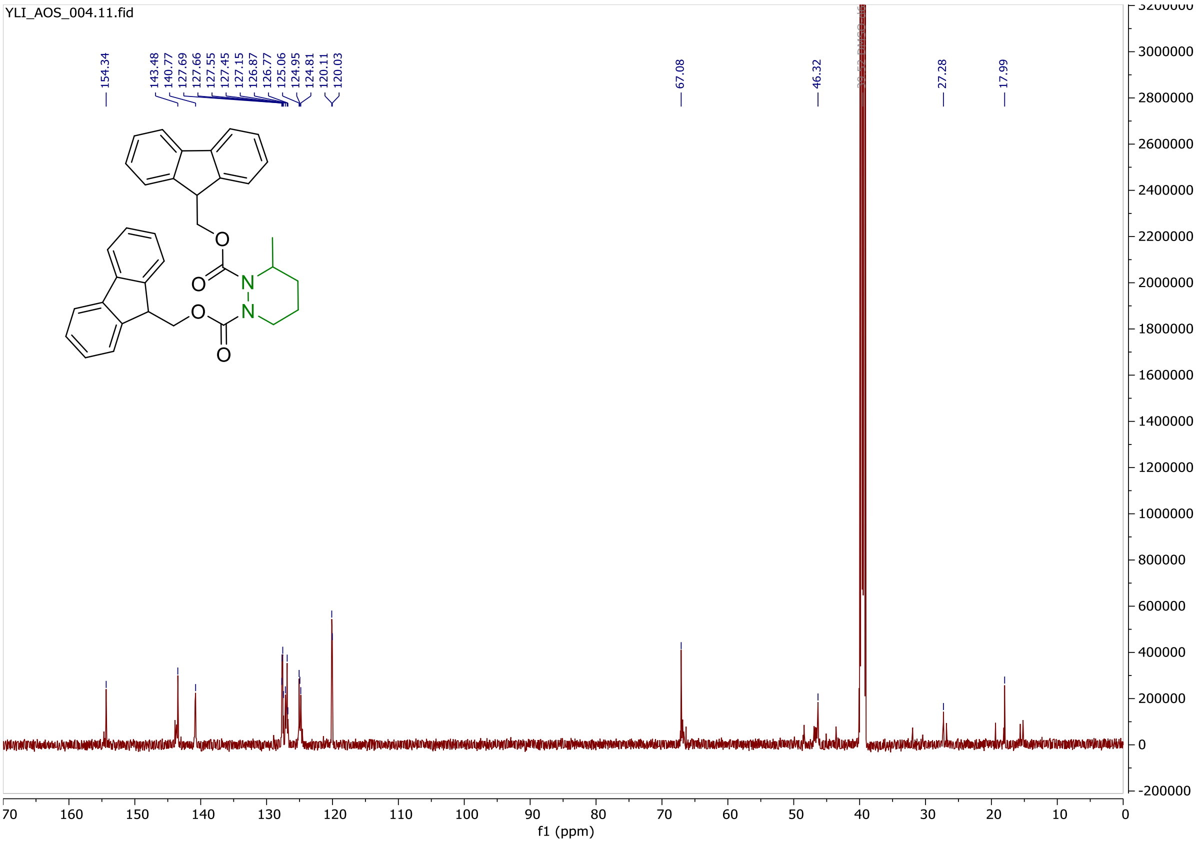

Comparison of ^{1}H spectra of ***enz*-Bis-Fmoc-11e** with the reference

Enzymatic reaction

Chemical reference

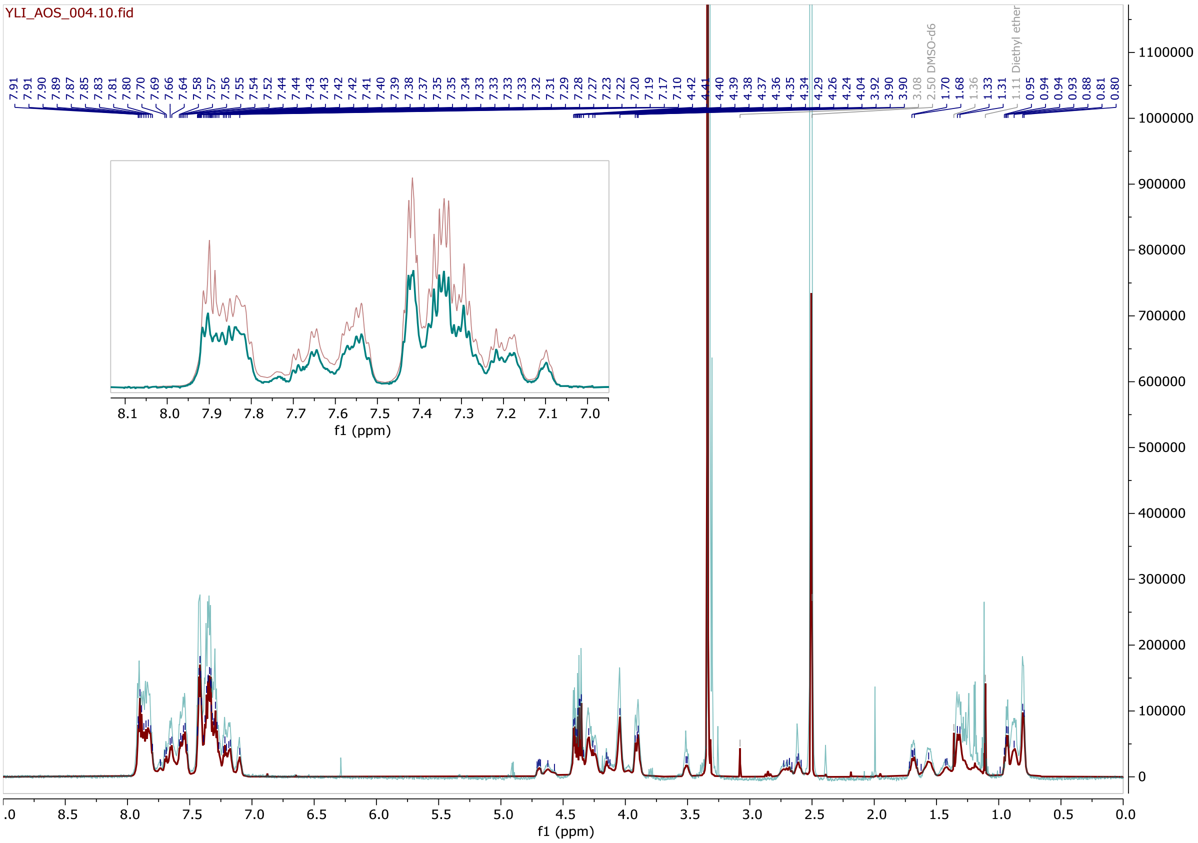

8. HRMS data of all compounds

1. **Bis((9*H*-fluoren-9-yl)methyl) pyrazolidine-1,2-dicarboxylate (Bis-Fmoc-11a)**

   Left: chemical reference; right: enzymatic synthesis

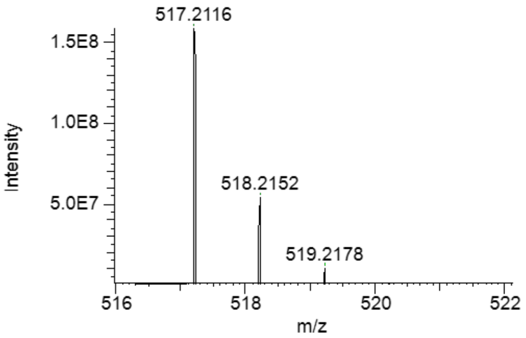

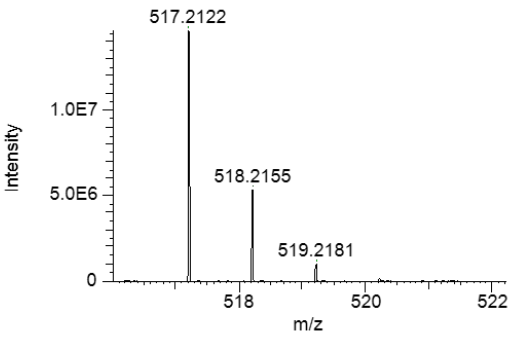

2. **Bis((9*H*-fluoren-9-yl)methyl) 3-methylpyrazolidine-1,2-dicarboxylate (Bis-Fmoc-11b)**

   Left: chemical reference; right: enzymatic synthesis

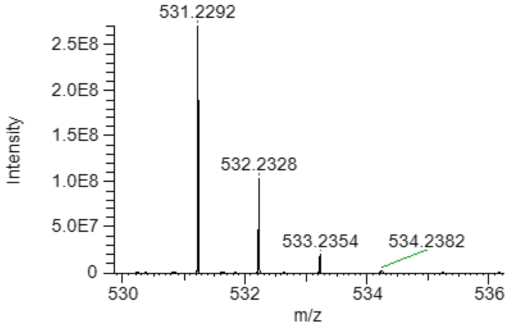

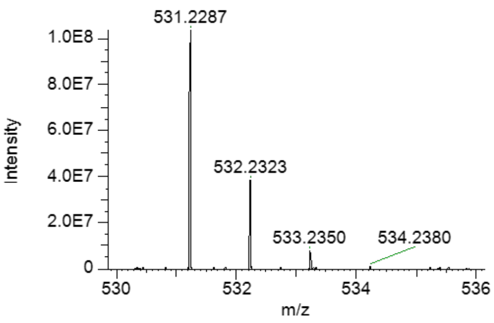

3. **Bis((9*H*-fluoren-9-yl)methyl) 3-ethylpyrazolidine-1,2-dicarboxylate (Bis-Fmoc-11c)**

   Left: chemical reference; right: enzymatic synthesis

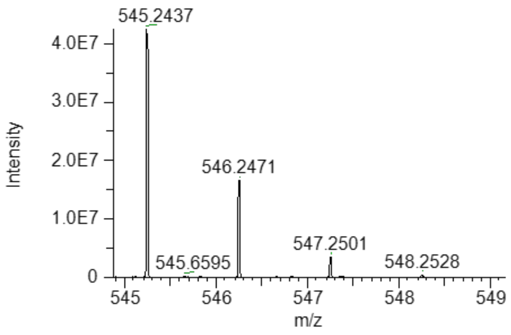

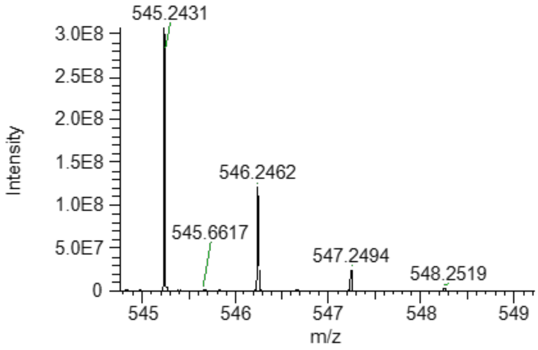

4. Bis((9*H*-fluoren-9-yl)methyl) tetrahydropyridazine-1,2-dicarboxylate (Bis-Fmoc-11d)

Left: chemical reference; right: enzymatic synthesis

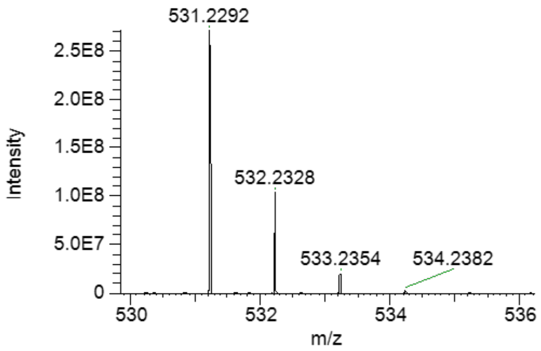

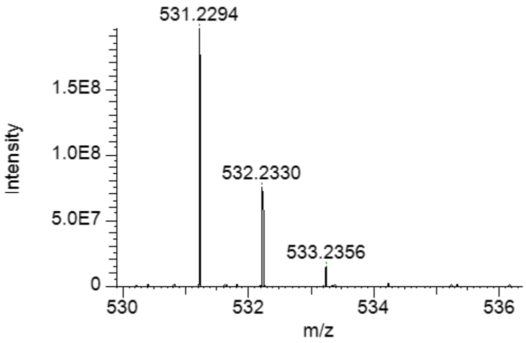

5. Bis((9*H*-fluoren-9-yl)methyl) 3-methyltetrahydropyridazine-1,2-dicarboxylate (Bis-Fmoc-11e)

Left: chemical reference; right: enzymatic synthesis

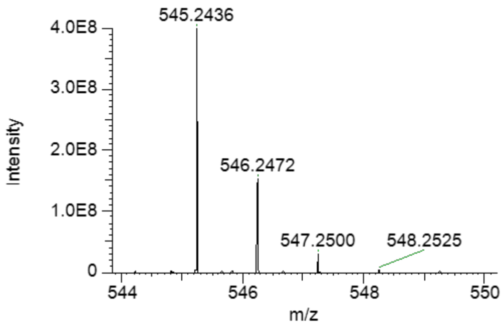

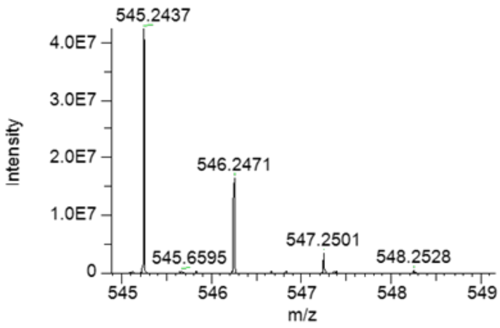

9. References

(1) Zallot, R.; Oberg, N.; Gerlt, J. A. The EFI Web Resource for Genomic Enzymology Tools: Leveraging Protein, Genome, and Metagenome Databases to Discover Novel Enzymes and Metabolic Pathways. *Biochem.* **2019**, *58* (41), 4169-4182.

(2) Oberg, N.; Zallot, R.; Gerlt, J. A. EFI-EST, EFI-GNT, and EFI-CGFP: enzyme function initiative (EFI) web resource for genomic enzymology tools. *J. Mol. Biol.* **2023**, *435* (14), 168018.

(3) Esuola, C. O.; Babalola, O. O.; Heine, T.; Schwabe, R.; Schlömann, M.; Tischler, D. Identification and characterization of a FAD-dependent putrescine *N*-hydroxylase (GorA) from Gordonia rubripertincta CWB2. *J. Mol. Catal. - B Enzym.* **2016**, *134*, 378-389.

(4) Blair, L. M.; Sperry, J. Natural Products Containing a Nitrogen–Nitrogen Bond. *J. Nat. Prod.* **2013**, *76* (4), 794.

(5) Hosaka, T.; Ohnishi-Kameyama, M.; Muramatsu, H.; Murakami, K.; Tsurumi, Y.; Kodani, S.; Yoshida, M.; Fujie, A.; Ochi, K. Antibacterial discovery in actinomycetes strains with mutations in RNA polymerase or ribosomal protein S12. *Nat. Biotechnol.* **2009**, *27* (5), 462-464.

(6) Yoshida, M.; Sekioka, N.; Izumikawa, M.; Kozone, I.; Takagi, M.; Shin-ya, K.; Doi, T. Total Synthesis and Structure Elucidation of JBIR-39: A Linear Hexapeptide Possessing Piperazic Acid and γ-Hydroxypiperazic Acid Residues. *Chem. Eur. J.* **2015**, *21* (7), 3031-3041.

(7) Williams, D. E.; Dalisay, D. S.; Patrick, B. O.; Matainaho, T.; Andrusiak, K.; Deshpande, R.; Myers, C. L.; Piotrowski, J. S.; Boone, C.; Yoshida, M.; et al. Padanamides A and B, Highly Modified Linear Tetrapeptides Produced in Culture by a Streptomyces sp. Isolated from a Marine Sediment. *Org. Lett.* **2011**, *13* (15), 3936-3939.

(8) Wyche, T. P.; Ruzzini, A. C.; Beemelmanns, C.; Kim, K. H.; Klassen, J. L.; Cao, S.; Poulsen, M.; Bugni, T. S.; Currie, C. R.; Clardy, J. Linear peptides are the major products of a biosynthetic pathway that encodes for cyclic depsipeptides. *Org. Lett.* **2017**, *19* (7), 1772-1775.

(9) Chandra, T.; Zebrowski, J. P.; Safety. A Safety Guidance Document for Lithium Aluminum Hydride (LAH) Reduction: A Resource for Developing Specific SOPs on LAH Manipulations. *J. Chem. Health Saf.* **2024**, *31* (2), 162-171.

(10) Saitou, N.; Nei, M. The neighbor-joining method: A new method for reconstructing phylogenetic trees. *Mol. Biol. Evol.* **1987**, *4*, 406-425.

(11) Dopazo, J. Estimating errors and confidence intervals for branch lengths in phylogenetic trees by a bootstrap approach. *J. Mol. Evol*. **1994**, *38*, 300-304.

(12) Rzhetsky, A.; Nei, M. A simple method for estimating and testing minimum evolution trees. *Mol. Biol. Evol*. **1992**, *9*, 945-967.

(13) Jones, D. T.; Taylor, W. R., Thornton, J. M. The rapid generation of mutation data matrices from protein sequences. *Comput. Appl. Biosci*. **1992**, *8*, 275-282.

(14) Kumar, S.; Stecher, G.; Suleski, M.; Sanderford, M.; Sharma, S.; Tamura, K. Molecular Evolutionary Genetics Analysis Version 12 for adaptive and green computing. *Mol. Biol. Evol*. **2024**, *41*, 1-9.
